# Influence of Lyophilization and Cryoprotection on the Stability and Morphology of Drug-Loaded Poly(ethylene glycol-*b*-ε-caprolactone) Micelles

**DOI:** 10.3390/polym15081974

**Published:** 2023-04-21

**Authors:** Md. Saddam Hussain, Khandokar Sadique Faisal, Andrew J. Clulow, Hugo Albrecht, Marta Krasowska, Anton Blencowe

**Affiliations:** 1 Applied Chemistry and Translational Biomaterials (ACTB) Group, Centre for Pharmaceutical Innovation (CPI), UniSA Clinical and Health Sciences, University of South Australia, Adelaide, SA 5000, Australia; 2 Australian Synchrotron, Australian Nuclear Science and Technology Organisation (ANSTO), 800 Blackburn Road, Clayton, Melbourne, VIC 3168, Australia; 3 Drug Delivery, Disposition & Dynamics, Monash Institute of Pharmaceutical Sciences, 381 Royal Parade, Parkville, Melbourne, VIC 3052, Australia; 4 Drug Discovery and Development Group, UniSA Clinical and Health Sciences, University of South Australia, Adelaide, SA 5000, Australia; 5 Future Industries Institute, UniSA STEM, University of South Australia, Mawson Lakes, Adelaide, SA 5095, Australia

**Keywords:** micelles, lyophilization, cryoprotection, reconstitution, redispersibility, β-CD

## Abstract

Polymeric micelles are promising carriers for the delivery of poorly water-soluble drugs, providing enhanced drug solubility, blood circulation times, and bioavailability. Nevertheless, the storage and long-term stability of micelles in solution present challenges requiring the lyophilization and storage of formulations in the solid state, with reconstitution immediately prior to application. Therefore, it is important to understand the effects of lyophilization/reconstitution on micelles, particularly their drug-loaded counterparts. Herein, we investigated the use of β-cyclodextrin (β-CD) as a cryoprotectant for the lyophilization/reconstitution of a library of poly(ethylene glycol-*b*-ε-caprolactone) (PEG-*b*-PCL) copolymer micelles and their drug-loaded counterparts, as well as the effect of the physiochemical properties of different drugs (phloretin and gossypol). The critical aggregation concentration (CAC) of the copolymers decreased with increasing weight fraction of the PCL block (*f_PCL_*), plateauing at ~1 mg/L when the *f_PCL_* was >0.45. The blank (empty) and drug-loaded micelles were lyophilized/reconstituted in the absence and presence of β-CD (9% *w*/*w*) and analyzed via dynamic light scattering (DLS) and synchrotron small-angle X-ray scattering (SAXS) to assess for changes in aggregate size (hydrodynamic diameter, *D_h_*) and morphology, respectively. Regardless of the PEG-*b*-PCL copolymer or the use of β-CD, the blank micelles displayed poor redispersibility (<10% relative to the initial concentration), while the fraction that redispersed displayed similar *D_h_* to the as-prepared micelles, increasing in *D_h_* as the *f_PCL_* of the PEG-*b*-PCL copolymer increased. While most blank micelles displayed discrete morphologies, the addition of β-CD or lyophilization/reconstitution generally resulted in the formation of poorly defined aggregates. Similar results were also obtained for drug-loaded micelles, with the exception of several that retained their primary morphology following lyophilization/reconstitution, although no obvious trends were noted between the microstructure of the copolymers or the physicochemical properties of the drugs and their successful redispersion.

## 1. Introduction

Polymeric micelles are nanosized aggregates that have been extensively studied for drug delivery [1,2]. In contrast to the static structure of solid nanoparticles, the core-shell dynamic structure of polymer micelles originates from the self-assembly of amphiphilic copolymer constituents. Therefore, solubilization dependence and physical instability (particulate aggregation, fusion, hydrolysis, and therapeutic leakage) during storage in solution remain major challenges for the widespread adoption of polymer micelles in drug delivery applications [3,4]. While lyophilization of polymer micelle solutions has been studied to address some of these issues and extend their shelf-life [5] as well as provide convenient handling, storage, and transport [6], reconstitution without irreversible aggregation remains challenging for many micelle-based systems. The success of lyophilization and reconstitution can be highly dependent on the micelle composition and preparation strategy, polymer composition and encapsulation properties, and lyophilization process parameters [7,8]. In the majority of cases, the addition of a cryoprotectant is necessary to protect micelles from stresses caused during lyophilization that can lead to irreversible secondary aggregation and fusion [9,10].

Typically reported cryoprotectants for the lyophilization of micelles to include saccharides, polymers (e.g., PEG), and surfactants. In particular, cyclodextrin (CD) derivatives including hydroxypropyl-α-CD (HP-α-CD), hydroxypropyl-β-CD (HP-β-CD), sulfobutylether-β-CD (SBE-β-CD), methyl-β-CD (M-β-CD), and hydroxypropyl-γ-CD (HP-γ-CD) have been widely used, as well as saccharides such as maltose, mannitol, sucrose, and trehalose. While the choice of cryoprotectant can depend on many different factors, it is often necessary to screen cryoprotectants for optimal performance with a chosen micelle formulation (i.e., copolymer and payload). Tommaso et al. report that 10% *w*/*v* sucrose was the most effective cryoprotectant for the lyophilization of cyclosporin-loaded PEG-*b*-poly(hexyl-lactide) (PEG-*b*-hexPLA) micelles [11], while 5% *w*/*v* mannitol was found to be more effective for bovine serum albumin (BSA)-loaded PEG-*b*-poly(lactic acid) (PEG-*b*-PLA) micelles [12].

PEG-*b*-PCL copolymer micelles have emerged as efficient and safe drug delivery platforms in preclinical studies due to their cytocompatibility, prolonged blood circulation, accumulation in tumors, low protein adsorption, and immunogenicity [13]. However, during storage in solution, blank and drug-loaded PEG-*b*-PCL micelles display relatively low physical stability regardless of the overall molecular weight, the weight fraction of the blocks, *f_PCL_*, or concentration [6,14]. The physical instability of PEG-*b*-PCL micelles during long-term storage has been associated with secondary aggregation, phase separation, copolymerization, and drug precipitation [6,10,14]. Therefore, lyophilization with appropriate cryoprotectants (alone or in combination) is a necessary step to maximize the physical stability of PEG-*b*-PCL micelles during mid- to long-term storage [14,15,16,17,18].

Moretton et al. studied the lyophilization of amphotericin-loaded PCL-*b*-PEG-*b*-PCL triblock copolymer micelles and found that lyophilization without a cryoprotectant resulted in an indispersible cake even under strong ultrasonication [6]. In comparison, the application of saccharides (maltose, glucose, or sorbitol), various molecular weight PEGs, and HP-β-CD as cryoprotectants improved redispersibility, with the latter providing a porous cake upon lyophilization that could easily be redisperse with a minimal increase (~10%) in the *D_h_* of the micelles [6]. Miller et al. compared the lyophilization of dexamethasone-loaded PEG-*b*-poly(4-vinylpyridine) (PEG-PVP) and blank PEG-*b*-PCL, PEG-*b*-PLA, and PEG-*b*-poly(lactide-co-glycolide) (PEG-*b*-PLGA) micelles with various cryoprotectants, including saccharides, Kolidon (poly(vinylpyrrolidone)), and β-CD derivatives (SBE-β-CD and HP-β-CD), and concluded that 1–2.5% *w*/*v* of HP-β-CD was most effective for redispersion of micelles, with similar *D_h_* and particle size distributions (PSDs) as the micelles before lyophilization [10]. The effectiveness of HP-β-CD over saccharides and Kolidon was also demonstrated by Richter et al. for the lyophilization and reconstitution of sagopilone-loaded PEG-*b*-PCL micelles [19]. Hu et al. further confirmed the ineffectiveness of simple saccharides (i.e., glucose) as cryoprotectants for PCL-*b*-PEG-*b*-PCL micelles [14]. 

The effectiveness of β-CD derivatives for the cryoprotection of PEG-*b*-PCL micelles has been attributed to host-guest complexation between the β-CD motif and PEG to form PEG-β-CD inclusion complexes [6,10]. The amorphous characteristics and relatively high collapse and glass transition temperatures (*T_c_* and *T_g_*, respectively) of CD derivatives have also been emphasized as important considerations [20], broadening the polymeric transition of the solid to rubbery states and providing the PEG component with more flexibility to endure the stresses imposed during lyophilization [6,10]. These investigations clearly emphasize the importance of cryoprotectants for the lyophilization and redispersion of PEG-*b*-PCL micelles; however, no studies have investigated a large and systematic library of PEG-*b*-PCL copolymers and whether a single cryoprotectant is suitable for polymers with different microstructures or micelles loaded with drugs that have different physicochemical properties. While β-CD derivatives have been widely employed for the cryoprotection of PEG-*b*-PCL micelles, the use of non-functionalized β-CD has not been investigated despite some favorable results with related systems. For example, Lee et al. reported that β-CD was effective as a cryoprotectant for the redispersion of eugenol-loaded Pluronic-based nanocapsules [21]. 

Thus, the purpose of this study was to investigate if the *f_PCL_* and PEG molecular weights influence the redispersion of PEG-*b*-PCL micelles following lyophilization in the absence and presence of hydrophobic drugs and β-CD as a cryoprotectant. The PEG-*b*-PCL micelles were analyzed via ultraviolet–visible (UV–vis) spectrophotometry, dynamic light scattering (DLS), and synchrotron small-angle X-ray scattering (SAXS) to determine and compare their ability to redisperse, self-assembly size distributions, and morphologies, respectively, following lyophilization and reconstitution.

## 2. Experimental

### 2.1. Materials

α-Methoxy-ω-hydroxy PEG (number-average molecular weight (*M_n_*) = 2 and 5 kDa), ε-caprolactone (ε-CL; ≥97%), deuterated chloroform (CDCl_3_; ≥99.8% D), deuterium oxide (D_2_O; ≥99.9% D), anhydrous toluene (≥99.8%), β-cyclodextrin (β-CD, ≥99%), stannous octoate (Sn(Oct)_2_; 92.5–100%), pyrene (≥98%), and lithium chloride (LiCl; ≥99%) were purchased from Sigma Aldrich (St. Louis, MO, USA). α-Methoxy-ω-hydroxy PEG (*M_n_* = 10 kDa) was purchased from Creative PEG Works (Chapel Hill, NC, USA). Phloretin (PH; 99%) and gossypol (GP; 99%) were purchased from Shaanxi Ciyuan Biotech (Xi’an, China). Analytical reagent grade acetone, diethyl ether (DEE), chloroform, toluene, ethyl acetate, tetrahydrofuran (THF), and *N*,*N*-dimethylformamide (DMF) were purchased from ChemSupply (Gilman, Australia). All chemicals were used as received unless otherwise stated. High-purity water was obtained from a Sartorius Arium^®^ Pro Ultrapure Water Systems UV-T-TOC (DEU) and had a resistivity of ≥18.2 MΩ·cm. Ultrahigh purity argon (99.99%) was purchased from BOC and passed through Drierite (W. A Hammond, Xenia, OH, USA) prior to use.

### 2.2. Characterization

Proton nuclear magnetic resonance (^1^H NMR) spectroscopy was performed at 25 ± 1 °C on a Bruker NMR AVANCE III HD 500 spectrometer (Bruker BioSpin, DEU) operating at 500 MHz. Polymers were analyzed in CDCl_3_ using the residual solvent peak (CHCl_3_; δ_H_ 7.26 ppm) as an internal reference. 

Gel permeation chromatography (GPC) was performed at 40 °C on a Prominence liquid chromatography system (Shimadzu, Japan) fitted with a Shimadzu RID-10A refractive index detector and two Shimadzu Shim-pack columns in series (GPC-8025D and GPC-805D). THF was used as the mobile phase at a flow rate of 1 mL/min. Samples were prepared at ~10 mg/mL and filtrated through 0.22 μm nylon syringe filters (Millipore, Burlington, MA, USA) prior to analysis. The number-average molecular weight (*M_n_*), weight-average molecular weight (*M_W_*), and dispersity (*Đ*)) of the polymers were determined with reference to a conventional column calibration with narrow *Đ* PEG standards (Polymer Standards Service GmbH; molar mass at peak maximum (*M_p_*) = 194 to 969 kDa). 

Critical aggregation concentrations (CACs) were determined using a Shimadzu RF-6000 fluorescence spectrometer at 25 ± 1 °C and pyrene as a fluorescent probe [22]. UV-vis spectrophotometry was conducted on a NanoDrop OneC (ThermoFischer, Waltham, MA, USA) to determine the extent of lyophilizate redispersion: % redispersibility = (absorbance of the lyophilized sample at peak maxima/absorbance of the non-lyophilized sample at peak maxima) × 100. pH measurements were performed with an Oakton pH 700 bench top pH meter with an Oakton all-in-one pH/ATC electrode, single junction, 12 mm probe (USA). 

The PSDs and the polydispersity index (PDI) of micelles were determined from correlograms using the Zetasizer 7.11 software and recorded using a Malvern Zetasizer NANO ZS (Malvern Panalytical, Malvern, UK) fitted with a 4 mW He–Ne laser source operating at a wavelength of 633 nm. 

SAXS measurements were performed at 37 °C using the SAXS/WAXS beamline at the Australian Synchrotron (Australia’s Nuclear Science and Technology Organisation (ANSTO), Melbourne, Australia) [23]. Samples were prepared in water, filtrated through 0.22 μM nylon syringe filters, and loaded into thin-walled glass capillaries (Charles Supper, Natick, MA, USA). The capillaries were placed inside a custom-made thermostatted capillary holder. Scattering patterns were recorded using a Dectris Pilatus 2M detector at sample-detector distances of 1350 mm (15.1 keV photon energy, 0.821 Å wavelength) and 7398 mm (11.0 keV photon energy, λ = 1.127 Å wavelength) [24]. 2D scattering patterns were reduced to profiles of scattered X-ray intensity [(I(Q)] versus the scattering vector Q [=(4π/λ)sinθ, 2θ = scattering angle] using ScatterBrain software (Australian Synchrotron). Data from the two detector configurations were combined into a single continuous scattering profile with a Q range from 1.9 × 10^−3^ to 2.0 × 10^−1^ Å^−1^ using the IRENA small-angle scattering macros (release 2.68) within the IgorPro software package (version 7.0.8.1) [25]. Multiplication of the background file was required for some samples because of the variable nature of the capillary thicknesses and the potential to take exposures closer to the edge of some capillaries than others in the moving stage. Thus, the sample path length of the X-ray beam was not the same for all samples, which in some cases led to an over-subtraction of the background signal. This was corrected by rescaling the background measurement.

### 2.3. Procedures

#### 2.3.1. Synthesis of PEG_x_-b-PCL_y_ Copolymers

PEG_x_PCL_y_ diblock copolymers (PEG_2_PCL_Y_, PEG_5_PCL_Y,_ and PEG_10_PCL_Y_ series) were synthesized as previously reported [24], whereby the subscript x and y refer to the *M_n_* (kDa) of the PEG and PCL blocks.

#### 2.3.2. Determination of CAC

Stock solutions of pyrene (6 × 10^−5^ M) and copolymers (2 mg/mL) were prepared in acetone. Specific volumes of the pyrene and copolymer solutions were combined in vials, and then high-purity water (2 mL) was added dropwise under gentle agitation to generate a series of solutions with copolymer concentrations ranging from 0.25 to 500 mg/L and a pyrene concentration of 6 × 10^−7^ M (Supplementary Information (SI), Table S1). The vials were heated at 60 °C on a heating block (Ratek Instruments Pty Ltd., Boronia, VIC, Australia) for 3 h and then placed on a vacuum line (0.1 mbar) for 1 h. The volume of the solutions was adjusted to 2 mL with high-purity water to account for any water evaporation during the removal of the acetone, and the solutions were filtered through 0.22 μm nylone syringe filters. Pyrene fluorescence emission spectra were recorded between 350 and 470 nm at 2000 nm/s and 3 nm bandwidth following excitation at λ_ex_ = 334 nm (SI, Figures S1–S3). The intensity ratio of emission peaks at λ_em_ = 372 and 384 nm (I_384_/I_372_) was plotted against the copolymer concentration, and the CAC was determined from the low concentration plateau.

#### 2.3.3. Preparation of Blank and Drug-Loaded Micelle Solutions

Blank and drug-loaded (GP and PH) micelles were prepared by the solvent evaporation technique (SI, Figure S4). For blank micelles, the copolymers (PEG_2_PCL_Y_, PEG_5_PCL_Y_, and PEG_10_PCL_Y_) were dissolved in acetone (1 mg/mL, 1 mL) in a glass vial, and high-purity water (1 mL) was added dropwise under gentle agitation. The vials were heated at 60 °C for 3 h in a heating block and subsequently placed under a vacuum (0.1 mbar) for 1 h to remove any residual acetone. High-purity water was added as required to adjust the concentration to 1 mg/mL. For drug-loaded micelles, stock solutions (1 mg/mL) of the copolymers (PEG_2_PCL_1.1_, PEG_2_PCL_1.8_, PEG_5_PCL_1.3_, PEG_5_PCL_2.4_, PEG_5_PCL_4.2_, PEG_5_PCL_9.5_, PEG_10_PCL_7.9_, and PEG_10_PCL_10.7_) and drugs (GP and PH) were prepared in acetone. The copolymer (0.9 mL) and drug (0.1 mL) stock solutions were combined in glass vials, and drug-loaded micelles (1 mg/mL, 10% *w*/*w* drug loading) were prepared as described previously for the blank micelles. All micelle solutions were obtained as transparent, homogenous solutions without any observable precipitation or sedimentation. The solutions were filtered through 0.22 µm nylon syringe filters prior to analysis. 

#### 2.3.4. Lyophilization and Reconstitution of Micelles

The micelle solutions were frozen at −80 °C for 1 h and then lyophilized (Alpha 1–2 LDplus, Martin Christ GmbH, DEU) at −57 °C (0.350 mbar) for 72 h, as previously reported [24]. The lyophilizates were stored in sealed vials at 23 ± 1 °C in the dark prior to analysis. For micelle lyophilizates containing β-CD as a cryoprotectant, a stock solution of β-CD (1 mg/mL) was prepared in ultrapure water, and 100 µL was added to each micelle solution prior to freezing and lyophilization to give 9% *w*/*w* of β-CD relative to the copolymer/drug. The lyophilized blank and drug-loaded (GP and PH) micelles were reconstituted with 1 mL of high-purity water under sonication (Soniclean Pty Ltd., SA, Australia) for 5 min, filtered through 0.22 µm nylon syringe filters, and then analyzed via DLS, SAXS, and UV-vis spectrophotometry.

## 3. Results and Discussion

Three copolymer series (PEG_2_PCL_Y_, PEG_5_PCL_Y_, and PEG_10_PCL_Y_), with *f_PCL_* between 0.1 and 0.7, were studied to determine if the PEG molecular weight or block size ratio influences the redispersibility of their micelles following lyophilization with and without cryoprotection. Phloretin (PH) (logP = 2.2 [26], logS = −3.32 [27]) and gossypol (GP) (logP = 4.5 [28], logS = −5.12 [27]) were selected as model drugs due to differences in hydrophobicity, allowing the influence of this parameter on micelle reconstitution to be studied. Micelles were prepared via the solvent evaporation approach, whereby the copolymers were dissolved in acetone and high-purity water was added, followed by the evaporation of the acetone to afford aqueous micelle solutions (1 mg/mL). For drug-loaded micelles, acetone solutions of the copolymer and drug were combined, and the same procedure was followed to produce micelle solutions (1 mg/mL, 10% *w*/*w* drug). All blank and drug-loaded micelle solutions were transparent to the naked eye, and the addition of β-CD (9% *w*/*w* relative to the micelles) resulted in no visible change. Following lyophilization and reconstitution in high-purity water with sonication, all micelle solutions were opaque and/or presented with visibly large aggregates. To allow characterization of the dispersed fraction of micelles, all solutions were filtered through 0.22 µm nylon syringe filters prior to analysis.

### 3.1. Critical Aggregation Concentration of PEG-b-PCL Copolymers

**Figure 1:**
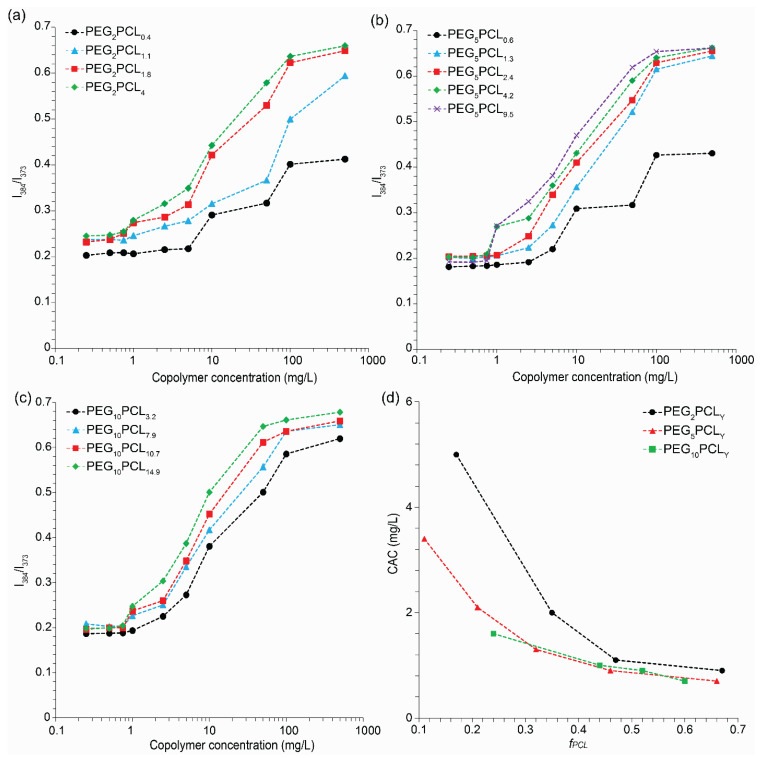
Ratiometric intensity (I_384_/I_372_) of the pyrene emission as a function of copolymer concentration in high-purity water for the (**a**) PEG_2_PCL_y_, (**b**) PEG_5_PCL_y_, and (**c**) PEG_10_PCL_y_ copolymer series. (**d**) CAC of the copolymers as a function of *f_PCL_*.

Initially, the CAC of the copolymers was determined using the pyrene probe method in conjunction with fluorescence spectroscopy. The pyrene emission spectra were measured as a function of copolymer concentration from 0.25 to 500 mg/L (SI, Figures S1–S3), and the emission intensity ratio (I_384_/I_372_) was plotted against the logarithmic copolymer concentration to estimate the CAC from the crossover of linear fits for the low concentration plateau and slope at intermediate concentrations (excluding the high concentration plateau) (Figure 1a–c). 

The emission spectra of most copolymer solutions displayed a narrowing of the first vibronic band (I_372_) of pyrene and the appearance of the third vibronic band (I_384_) with increasing copolymer concentration, indicating a decrease in the polarity of the pyrene microenvironment [29,30] associated with aggregation of the PCL block and the formation of micelles (SI, Figures S1–S3). Interestingly, the pyrene emission spectra for PEG_2_PCL_0.4_ and PEG_5_PCL_0.6_ did not reveal any changes in fine structure despite an apparent increase in the I_384_/I_372_ ratio, suggesting that these polymers do form defined aggregates with isolated hydrophobic regions, consistent with previous observations that they form metastable clusters [24]. The CAC was found to decrease with increasing *f_PCL_* for all copolymer series, beginning to plateau between 0.9 and 1.1 mg/L when the *f_PCL_* > 0.45 (Figure 1d). These results are consistent with previously reported CACs for PEG-*b*-PCL diblock copolymers [22,31,32,33,34,35,36,37,38,39]. For instance, Choi et al. reported that the CAC of PEG_2_PCL_0.5_, PEG_2_PCL_1_, and PEG_2_PCL_1.5_ copolymers decreased from 5.95 to 0.83 mg/L as the *f_PCL_* was increased [22]. 

The micelles were analyzed under isothermal conditions, so temperature effects could be eliminated. While low-molecular-weight PEG-PCL copolymers (and more specifically PCL-PEG-PCL triblock copolymers) with high *f_PCL_* can display lower critical solution temperature (LCST) values in the relevant range for biological applications (ambient to 37 °C) [40,41,42], none of the diblock copolymers studied in this manuscript have similar molecular weights and *f_PCL_*. For higher molecular weight PEG-PCL copolymers, it is possible for them to display LCSTs, but these are much higher than physiological temperatures and not relevant for drug-loaded micelles. For these reasons, the thermal properties of the micelles in the solution were not studied.

### 3.2. Effects of Lyophilization and Reconstitution on Blank Micelles

The percentage of blank micelles redispersed was determined by measuring the absorbance at the peak maxima (λ_max_ ≈ 264 nm) of the micelles before and after reconstitution via UV-vis spectrophotometry. In the absence of β-CD, the redispersibility of the blank micelles was found to be very poor (typically < 10%), and there were no obvious trends between the redispersibility and the PEG molecular weight or *f_PCL_*. The addition of β-CD did not provide any significant improvement in the redispersibility. Evidently, the majority of the blank micelles are present as larger aggregates after reconstitution and are removed following filtration through 0.22 µm syringe filters. 

During this study, we intentionally avoided the use of electron microscopy (e.g., SEM and TEM), as this requires sample preparation (e.g., adsorption of the micelles to substrates, dehydration, staining, and sputter coating) that can distort the micelle morphology, cause disassembly and aggregation, and lead to erroneous results that are not truly representative of the micelles in solution [43]. Furthermore, SEM lacks the resolution to clearly observe small micelles [44,45]. In comparison, non-interfering techniques (such as the light scattering techniques reported herein) allow the characterization of micelles in their native and solvated states at ambient temperatures [24,43].

**Figure 2:**
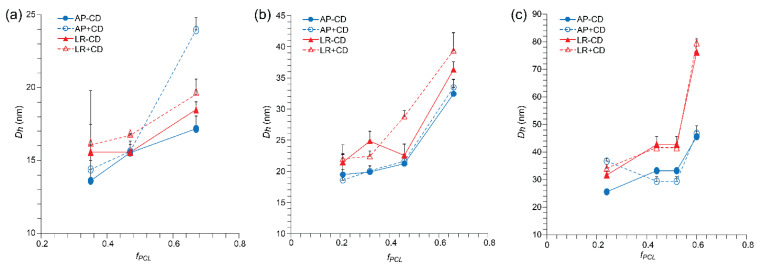
*D_h_* versus *f_PCL_* as determined from the number PSDs for the (**a**) PEG_2_PCL_Y_, (**b**) PEG_5_PCL_Y_, and (**c**) PEG_10_PCL_Y_ micelles as prepared (AP), after lyophilization/reconstitution (LR), and in the absence and presence of β-CD (−/+CD). All values are reported as the mean + std. dev. (*n* = 3).

The *D_h_* and PDI values (SI, Table S2) of the blank micelles were determined from DLS number PSDs before (as prepared (AP)) and after lyophilization/reconstitution (LR) in the absence and presence of β-CD (−/+ CD). While the number of PSDs was generally narrow, volume and intensity PSDs were typically broad and, in many cases, multimodal, indicative of various populations of micelles and secondary aggregates (SI, Figures S5–S7). For the as-prepared micelles, the *D_h_* was found to generally increase as the PEG molecular weight or *f_PCL_* was increased (Figure 2), with the exception of the PEG_2_PCL_0.4_ and PEG_5_PCL_0.6_ copolymers (SI, Figures S5 and S6), which have previously been shown to form metastable clusters rather than well-defined micelles [24]. In most cases, the addition of β-CD before lyophilization had a negligible effect on the micelle *D_h_*, which might indicate that the β-CD is solvated in solution and there is no significant inclusion complexation between the PEG corona of the micelles and the β-CD. While lyophilization and reconstitution in the absence or presence of β-CD did not significantly change the *D_h_* of the redisperse micelles for the PEG_2_PCL_y_ series when the *f_PCL_* was >0.2, a marked increase was noted for many of the PEG_5_PCL_y_ and PEG_10_PCL_y_ micelles (Figure 2). The PDI values of the as-prepared micelles ranged between 0.12 and 0.26, with polymers of higher PEG molecular weight and larger *f_PCL_* generally having lower values (SI, Table S2). The addition of β-CD before lyophilization resulted in slight changes in the PDI values of the micelles, although no consistent trends were observed. Similarly, following lyophilization/reconstitution in the absence or presence of β-CD, the majority of the PDI values of the redispersed fraction of micelles ranged between 0.12 and 0.25, with no obvious trends resulting from the use of cryoprotectant. In comparison, Richter et al. reported an increase in the PDI of PEG_2_PCL_2.6_ and PEG_5_PCL_5_ micelles following lyophilization and reconstitution with HP-β-CD as the cryoprotectant, even after filtration (0.22 μm syringe filters) [19]. 

**Figure 3:**
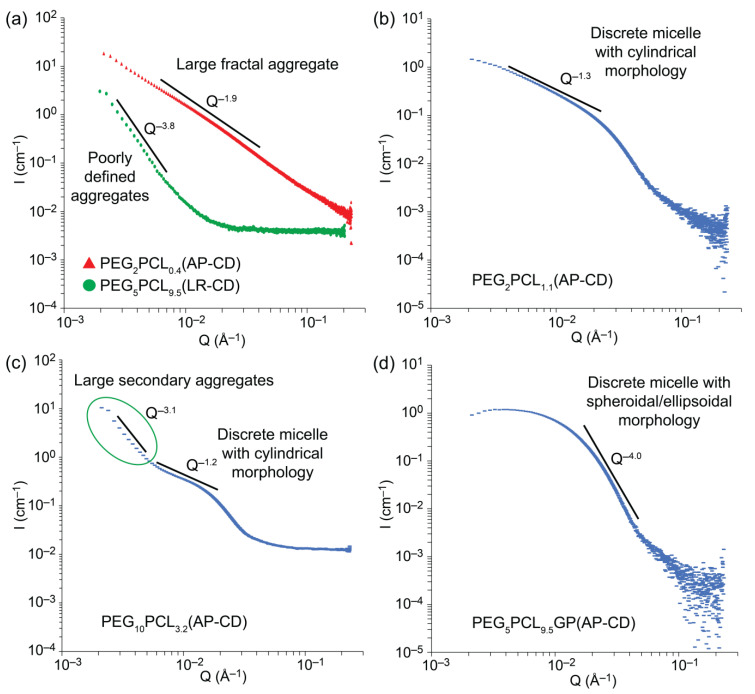
Representative SAXS scattering profiles complete with power laws to indicate the morphology of the aggregates or micelles: (**a**) PEG_2_PCL_0.4_ (red profile), (**b**) PEG_2_PCL_1.1,_ and (**c**) PEG_10_PCL_3.2_ as prepared in the absence of β-CD, (**a**) PEG_5_PCL_9.5_ (green profile) following lyophilization/reconstitution in the absence of β-CD, and (**d**) gossypol-loaded PEG_5_PCL_9.5_ as prepared in the absence of β-CD.

To assess if the morphology of the redispersed micelles was retained following lyophilization and reconstitution, synchrotron small-angle X-ray scattering (SAXS) was employed. Previously, we observed from SAXS experiments that the PEG-*b*-PCL assemblies transitioned from loosely associated unimers and metastable aggregates to well-defined micelles with cylindrical or ellipsoidal morphologies as the *f_PCL_* increased [24]. For example, PEG_5_PCL_Y_ copolymers with *f_PCL_* < 0.2 form metastable aggregates, while *f_PCL_* between 0.2 and 0.45 forms cylindrical micelles, and *f_PCL_* > 0.45 forms ellipsoidal micelles. These previous results and modeling [22] were used as a point of reference when considering morphological changes in the presence of β-CD or following lyophilization/reconstitution. With the exception of PEG_2_PCL_0.4_ solutions (Figure 3a), the SAXS profiles of the as prepared PEG_2_PCL_y_ micelles prior to the addition of β-CD indicated the presence of primary cylindrical micelles as indicated by the ~Q^−1^ power law in the intermediate Q region (Figure 3b and SI, Figure S8), with some profiles also displaying an upturn at low Q consistent with scattering from larger secondary aggregates (as indicated on Figure 3c). Following the addition of β-CD or after lyophilization/reconstitution, the form factors generally remained similar, suggesting that the morphology of the primary micelles was conserved, although the upturn at low Q became more pronounced, indicative of an increase in large secondary aggregates not removed by filtration. In contrast, the SAXS profiles of PEG_2_PCL_0.4_ revealed a power law increase in scattering with decreasing Q, and an exponent of Q^−1.9^ corresponds to large fractal aggregates, further confirming the absence of well-defined micelles in this system. The SAXS profile remained unchanged following lyophilization/reconstitution; however, the addition of β-CD led to an increase in background scattering at high Q, likely from β-CD. Similar behavior was observed for the PEG_5_PCL_0.6_ copolymer (SI, Figure S9). 

For the as prepared PEG_5_PCL_y_ micelles, when the *f_PCL_* > 0.11, the SAXS profiles indicated the presence of discrete primary micelles, accompanied by larger secondary aggregates (SI, Figure S9). While the addition of β-CD or lyophilization/reconstitution did not significantly change the morphology of the PEG_5_PCL_1.3_ micelles, the scattering profiles for PEG_5_PCL_2.4_, PEG_5_PCL_4.2_, and PEG_5_PCL_9.5_ revealed the disappearance of discrete form factors and presented solely with power laws at low Q consistent with large poorly defined aggregates (Figure 3b). These results suggest that the addition of β-CD disrupts the micelle morphology when the *f_PCL_* > 0.20 for the PEG_5_PCL_y_ micelles. Lyophilization/reconstitution of these micelles in the absence and presence of β-CD were also found to result in similar morphological changes. Similar trends were also noted for the PEG_10_PCL_y_ series (SI, Figure S10), indicating that even prior to lyophilization/reconstitution, the addition of β-CD promoted significant aggregation and morphological changes in the micelles when the PEG *M_n_* was >2 kDa. This behavior may be related to the formation of pseudo-rotaxanes between the β-CD and the PEG corona of the micelles, with subsequent hydrogen-bonding between the β-CD leading to aggregation of the micelles [46,47,48,49]. β-CD is also known to form inclusion complexes with PCL, which may also contribute to the disruption of the micelle morphology [50,51,52,53]. An outlier in the PEG_10_PCL_y_ series was the PEG_10_PCL_10.7_ micelle, which following lyophilization/reconstitution in the absence of β-CD presented with similar primary morphology.

Tommaso et al. have also reported changes in the primary morphology of PEG-*b*-hexPLA micelles after lyophilization/reconstitution in the absence of a cryoprotectant or the presence of certain saccharide cryoprotectants (i.e., glucose, mannitol, and trehalose). Specifically, a morphological change from spherical to worm-like aggregates was observed via transmission electron microscopy, TEM [11]. Miller et al. compared the morphologies of PEG_5_PLA_23_, PEG_5_PLGA_28_, and PEG_5_PCL_33_ micelles before and after lyophilization with or without SBE-β-CD and HP-β-CD cryoprotectants via cryo-TEM [10]. While HP-β-CD was effective in retaining the primary spherical morphology of all micelles, SBE-β-CD was only effective for PEG_5_PLGA_28_ micelles [10], highlighting the variability between β-CD derivatives for different micelle compositions. 

### 3.3. Effects of Lyophilization and Reconstitution on Drug-Loaded Micelles

Initially, the incorporation of drugs (10% *w*/*w*) into the micelles was investigated using the PEG_5_PCL_y_ series, with the exclusion of PEG_5_PCL_0.6_ as it did not form well-defined micelles. Following lyophilization and reconstitution, the percentage redispersibility of the drug-loaded PEG_5_PCL_y_ micelles was slightly improved as compared to their blank counterparts (<20%), with the addition of β-CD providing no obvious improvement. The slightly improved redispersibility of the drug-loaded micelles (cf. blank micelles) implies that the drugs either provide some stabilization to the micelles or promote the redispersion of aggregates, even in the absence of a cryoprotectant. While some prior literature supports this observation, in general, the redispersibility appears to be highly dependent on the combination of the micelle, cryoprotectant, and drug [10,19]. Miller et al. reported that the redispersibility of PEG_5_PCL_33_ micelles was improved after lyophilization/reconstitution when loaded with dexamethasone (cf. blank micelles) in the absence and presence of certain cryoprotectants [10]. In contrast, Richter et al. reported that sagopilone-loaded PEG_2_PCL_2.6_ and PEG_5_PCL_5_ micelles were not readily dispersible following lyophilization/reconstitution, even in the presence of HP-β-CD as the cryoprotectant [19]. Tommaso et al. demonstrated that cyclosporin-A (CsA)-loaded PEG-*b*-hexPLA micelles displayed better redispersibility after lyophilization with sucrose as a cryoprotectant, whereas trehalose and glucose were less effective [11]. 

**Figure 4:**
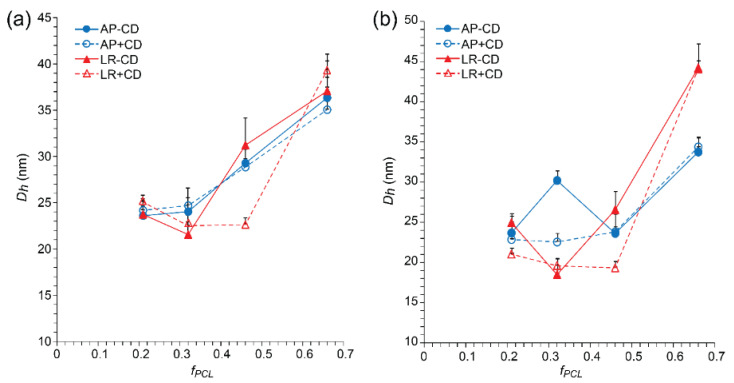
*D_h_* versus *f_PCL_* as determined from number PSDs for (**a**) gossypol (GP) and (**b**) phloretin (PH)-loaded PEG_5_PCL_1.3_, PEG_5_PCL_2.4,_ PEG_5_PCL_4.2_, and PEG_5_PCL_9.5_ micelles as prepared (AP), after lyophilization/reconstitution (LR), and in the absence and presence of β-CD (−/+CD). All values are reported as the mean + std. dev. (*n* = 3).

For the populations of the drug-loaded micelles that were redispersed after reconstitution, the *D_h_* values were generally similar to those of the micelles prior to lyophilization and followed the same trend of increasing *D_h_* with increasing *f_PCL_* observed for the blank micelles. Compared to the blank micelles (Figure 2), the addition of the drugs resulted in a slight increase in the *D_h_* of the micelles by 2–10 nm (Figure 4 and SI, Figures S11–S14), consistent with drug encapsulation. The addition of β-CD to the drug-loaded micelles did not significantly influence the *D_h_*. 

The SAXS profiles of the as-prepared drug-loaded PEG_5_PCL_y_ copolymers were consistent with discrete primary micelles with some larger secondary aggregates, except for PEG_5_PCL_9.5_ micelles, which were free of secondary aggregates (Figure 3d and SI, Figures S15–S18). The addition of β-CD had little influence on the scattering profiles as compared to their blank micelle counterparts, implying that drug loading inhibits the β-CD-promoted reorganization of the micelle morphology. Lyophilization/reconstitution of the micelles in the absence of β-CD led to disruption of the morphology and the formation of poorly defined aggregates regardless of the specific drug. Similar results were obtained for the lyophilization/reconstitution in the presence of β-CD, whereby it is conceivable that β-CD may also contribute to the disruption of the morphology through the formation of inclusion complexes with the drugs [54,55]. A notable exception was the GP-loaded PEG_5_PCL_9.5_ micelles that retained structural features despite increased secondary aggregates (SI, Figure S18). Thus, it appears that *f_PCL_* ≥ 0.66 in the presence of very lipophilic drugs and β-CD provides some benefits for the redispersion of micelles. 

While the influence of drug loading on the morphology of polymeric micelles has received limited attention, several studies have reported improved micelle stability following drug loading [56,57]. Akiba et al. investigated the structure of partially benzyl-esterified PEG-*b*-poly(aspartic acid) using SAXS before and after the loading of LE540 (a hydrophobic retinoid antagonist), whereby the polymer formed a hexagonal arrangement of α-helices that transitioned to spherical micelles upon drug loading [56]. Chang et al. reported that PEG-*b*-poly(styrene) micelles displayed enhanced stability after curcumin loading with a reduced tendency to aggregate [40]. Similarly, Hu et al. and Moretton et al. have noted that PEG-*b*-PCL micelles display improved stability upon the loading of hydrophobic drugs [14,58]. 

To investigate potential differences between copolymers with variable PEG block length and drug-loaded micelles, they were compared with copolymers with a fixed *f_PCL_* of ~0.5 (i.e., PEG_2_PCL_1.8_, PEG_5_PCL_4.2_, and PEG_10_PCL_10.7_). DLS analysis revealed that the population of redispersed micelles displayed similar *D_h_* values to the micelles prior to lyophilization (SI, Figures S19–S22). For the PEG_2_PCL_1.8_ and PEG_5_PCL_4.2_ drug-loaded micelles, the *D_h_* was ~20–30 nm regardless of the identity of the drug, with no effect observed from the addition of β-CD. After lyophilization/reconstitution of the PEG_10_PCL_10.7_ drug-loaded micelles, the *D_h_* increased to ~55–65 nm from ~45 nm, regardless of the drug identity, suggesting that the larger molecular weight PEG is prone to forming secondary aggregates that cannot be completely redispersed. 

The SAXS profiles of the as-prepared drug-loaded PEG_2_PCL_1.8_ micelles (SI, Figure S23) were very similar to their blank counterparts (SI, Figure S8) and indicated the presence of cylindrical/ellipsoidal primary aggregates with some larger secondary aggregates. The addition of β-CD had no effect. In contrast to the drug-loaded PEG_5_PCL_4.2_ micelles, the drug-loaded PEG_2_PCL_1.8_ micelles retained a discrete morphology after lyophilization/reconstitution, although an increase in secondary aggregates was observed for the PH-loaded PEG_2_PCL_1.8_ micelles in the presence of β-CD. The as-prepared drug-loaded PEG_10_PCL_10.7_ micelles presented with discrete primary morphologies, although larger secondary aggregates were only noted for the GP-loaded micelles (SI, Figure S24). The addition of β-CD increased the extent of secondary aggregation for the GP-loaded PEG_10_PCL_10.7_ micelles but had no effect on the PH-loaded micelles, which is significantly different from the behavior of the blank PEG_10_PCL_10.7_ micelles, where the primary morphology was lost (SI, Figure S10). Thus, the addition of lipophilic drugs helps to prevent β-CD mediated disruption of the morphology of the PEG_10_PCL_10.7_ micelles (cf. PEG_5_PCL_4.2_ micelles). Surprisingly, lyophilization/reconstitution of the GP-loaded PEG_10_PCL_10.7_ micelles resulted in discrete morphologies, whereas the PH-loaded PEG_10_PCL_10.7_ micelles formed poorly defined aggregates. The addition of β-CD had no influence on this behavior, indicating that the lipophilicity of the drug plays a role in retaining the primary morphology for this particular polymer. 

## 4. Conclusions

Micelles were prepared from a library of PEG-*b*-PCL copolymers with various PEG molecular weights and *f_PCL_*, and the effect of lyophilization and reconstitution on their redispersibility, *D_h_*, and morphology were studied in the absence and presence of β-CD as a cryoprotectant. The *D_h_* of the blank micelles was found to generally increase with increasing PEG molecular weights and *f_PCL_*, and when the *f_PCL_* > 0.2, the micelles presented with discrete morphologies. While the loading of model drugs (gossypol or phloretin) led to a slight increase in the micelle *D_h_*, the morphology remained unaffected. Following lyophilization and reconstitution, the blank and drug-loaded micelles were poorly dispersible, although drug loading did lead to a slight improvement (<10% vs. <20%). DLS of the redispersible fraction revealed slight changes in the micelle *D_h_*, although there were no obvious trends related to the PEG molecular weight or *f_PCL_*. For blank micelles, the primary morphology of the PEG_2_PCL_y_ series was retained after lyophilization and reconstitution, while the higher molecular weight PEG series predominately formed poorly defined aggregates. The addition of β-CD to the blank micelles provided no improvement in the retention of the morphology and, in several cases, even promoted reorganization of the micelle structure prior to lyophilization and reconstitution. Loading of the micelles with drugs helped to protect against β-CD promoted reorganization, although subsequent lyophilization and reconstitution generally resulted in the formation of poorly defined aggregates, with the exception of gossypol-loaded PEG_2_PCL_1.8_ and PEG_10_PCL_10.7_ micelles. These results highlight the ineffectiveness of β-CD as a cryoprotectant under the conditions studied, and further studies with a range of cryoprotectants and loadings are required to fully interpret the contribution of any polymer microstructure-behavior relationships to the success of lyophilization and reconstitution.

## Data Availability

The data presented in this study are available on request from the corresponding author.

## Supplementary Materials

The following supporting information can be downloaded at: https://www.mdpi.com/article/10.3390/polym15081974/s1, Figure S1: Pyrene fluorescent emission spectra for the PEG_2_PCL_y_ copolymer series; Figure S2: Pyrene fluorescent emission spectra for the PEG_5_PCL_y_ copolymer series.; Figure S3: Pyrene fluorescent emission spectra for the PEG_10_PCL_y_ copolymer series; Figure S4: Illustration of preparation, lyophilization and characterisation of PEG_X_-*b*-PCL_Y_ micelles; Figure S5: Dynamic light scattering (DLS) particle size distributions (PSDs) for blank PEG_2_PCL_y_ micelles; Figure S6: DLS PSDs for blank PEG_5_PCL_y_ micelles; Figure S7: DLS PSDs for blank PEG_10_PCL_y_ micelles; Figure S8: Small-angle X-ray scattering (SAXS) profiles for blank PEG_2_PCL_y_ micelles; Figure S9: SAXS profiles for blank PEG_5_PCL_y_ micelles; Figure S10: SAXS profiles for blank PEG_10_PCL_y_ micelles; Figure S11: DLS PSDs for drug-loaded PEG_5_PCL_1.3_ micelles; Figure S12: DLS PSDs for drug-loaded PEG_5_PCL_2.4_ micelles; Figure S13: DLS PSDs for drug-loaded PEG_5_PCL_4.2_ micelles; Figure S14: DLS DSDs for drug-loaded PEG_5_PCL_9.5_ micelles; Figure S15: SAXS profiles for drug-loaded PEG_5_PCL_1.3_ micelles; Figure S16: SAXS profiles for drug-loaded PEG_5_PCL_2.4_ micelles; Figure S17: SAXS profiles for drug-loaded PEG_5_PCL_4.2_ micelles; Figure S18: SAXS profiles for drug-loaded PEG_5_PCL_9.5_ micelles; Figure S19: DLS PSDs for drug-loaded PEG_2_PCL_1.8_ micelles; Figure S20: DLS PSDs for drug-loaded PEG_10_PCL_10.7_ micelles; Figure S21: Hydrodynamic diameter (*D_h_*) versus weight fraction of PCL (*f_PCL_*) as determined from number PSDs for drug-loaded PEG_2_PCL_1.8_ micelles; Figure S22: *D_h_* versus *f_PCL_* as determined from number PSDs for drug-loaded PEG_10_PCL_10.7_ micelles; Figure S23: SAXS profiles for drug-loaded PEG_2_PCL_1.8_ micelles; Figure S24: SAXS profiles for drug-loaded PEG_10_PCL_10.7_ micelles; Table S1: Determination of the CAC of PEG-*b*-PCL block copolymer micelles; Table S2: Summary of the PDI values of the blank and drug-loaded PEG-*b*-PCL micelles.
