# Supplementary material for: Influence of Lyophilization and Cryoprotection on the Stability and Morphology of Drug-Loaded Poly(ethylene glycol-b-ε-caprolactone) Micelles
Source: Polymers (Basel). 2023 Apr 21;15(8):1974. doi: 10.3390/polym15081974 (PMC10146133; doi:10.3390/polym15081974)
Supplement: Supplementary file 1 [file polymers-15-01974-s001.zip › polymers-2306443-supplementary.docx]

Supplementary Information

Influence of Lyophilization and Cryoprotection on the Stability and Morphology of Drug-Loaded Poly(Ethylene Glycol-*b*-ε-caprolactone) Micelles

**Table S1.** Concentration and volume of stock solutions combined to prepare copolymer solutions in ultrapure water for the determination of the CAC of PEG-*b*-PCL block copolymer micelles.

| Conc. of copolymer in PBS (mg/L)^a^ | Volume of pyrene stock solution (µL)^b^ | Conc. of copolymer stock solution (mg/L)^c^ | Volume of copolymer stock solution (µL) | Volume of ultrapure water added (µL) |
| --- | --- | --- | --- | --- |
| 500 | 20 | 2000 | 500 | 2000 |
|  |  |  |  |  |
|  |  |  |  |  |
| 100 | 20 | 2000 | 100 | 2000 |
|  |  |  |  |  |
|  |  |  |  |  |
| 50.0 | 20 | 1000 | 100 | 2000 |
|  |  |  |  |  |
|  |  |  |  |  |
| 10.0 | 20 | 1000 | 20 | 2000 |
|  |  |  |  |  |
|  |  |  |  |  |
| 5.00 | 20 | 1000 | 10 | 2000 |
|  |  |  |  |  |
|  |  |  |  |  |
| 2.50 | 20 | 100 | 50 | 2000 |
|  |  |  |  |  |
|  |  |  |  |  |
| 1.00 | 20 | 100 | 20 | 2000 |
|  |  |  |  |  |
|  |  |  |  |  |
| 0.75 | 20 | 100 | 15 | 2000 |
|  |  |  |  |  |
|  |  |  |  |  |
| 0.50 | 20 | 100 | 10 | 2000 |
|  |  |  |  |  |
|  |  |  |  |  |
| 0.25 | 20 | 10 | 50 | 2000 |
|  |  |  |  |  |
|  |  |  |  |  |

^a^ Copolymer solutions were prepared in high purity water *via* the solvent evaporation approach with a constant pyrene concentration of 6 × 10^-7^ M. ^b^ A 6 × 10^-5^ M pyrene stock solution in acetone was used to prepare all solutions. ^c^ All stock solutions were prepared in acetone.


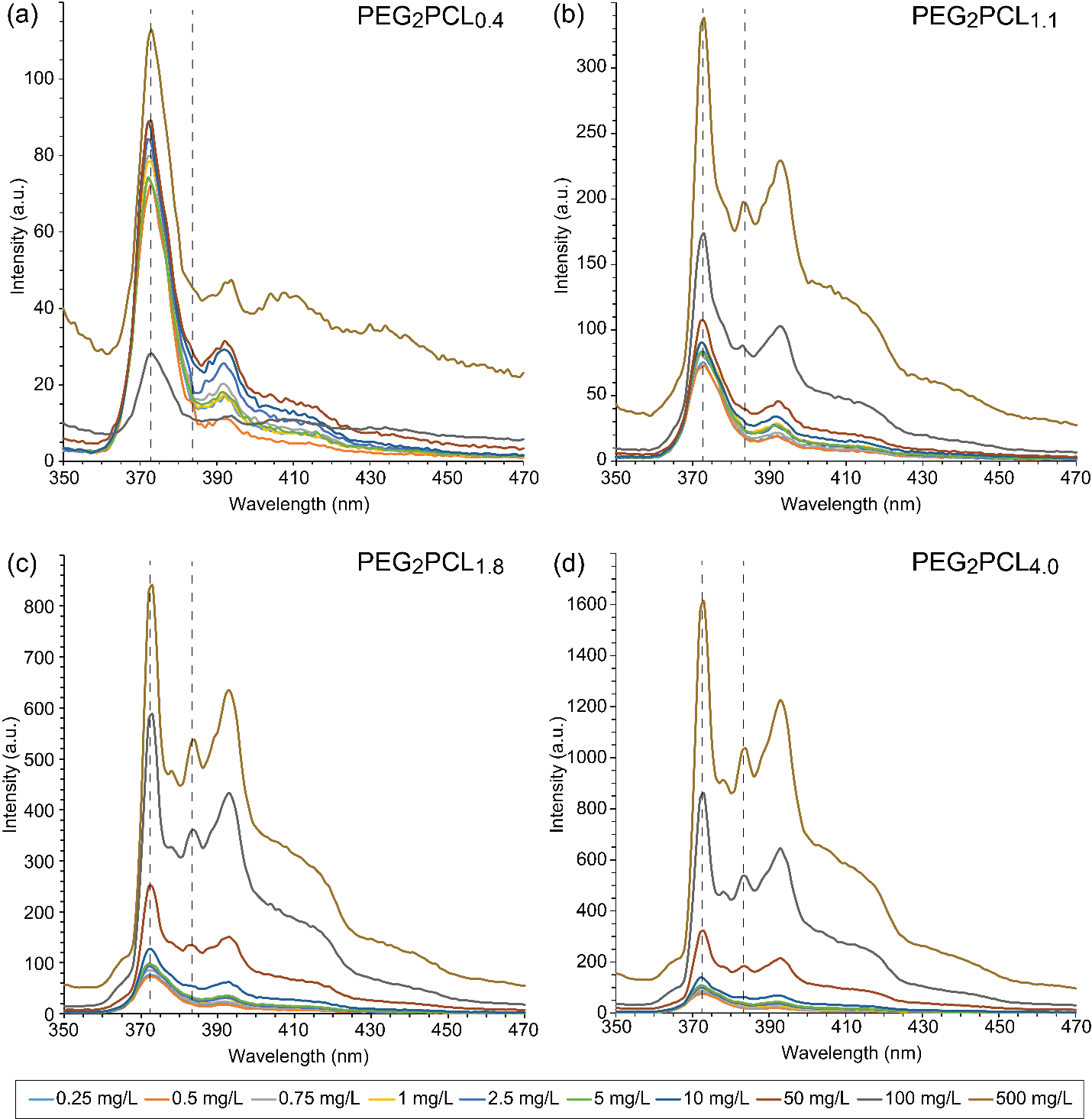


**Figure S1.** Pyrene (6 x 10^-7^ M) fluorescent emission spectra (λ_ex_ = 334 nm) for (a) PEG_2_PCL_0.4_, (b) PEG_2_PCL_1.1_, (c) PEG_2_PCL_1.8_ and (d) PEG_2_PCL_4.0_ copolymers at various copolymer concentrations.

**
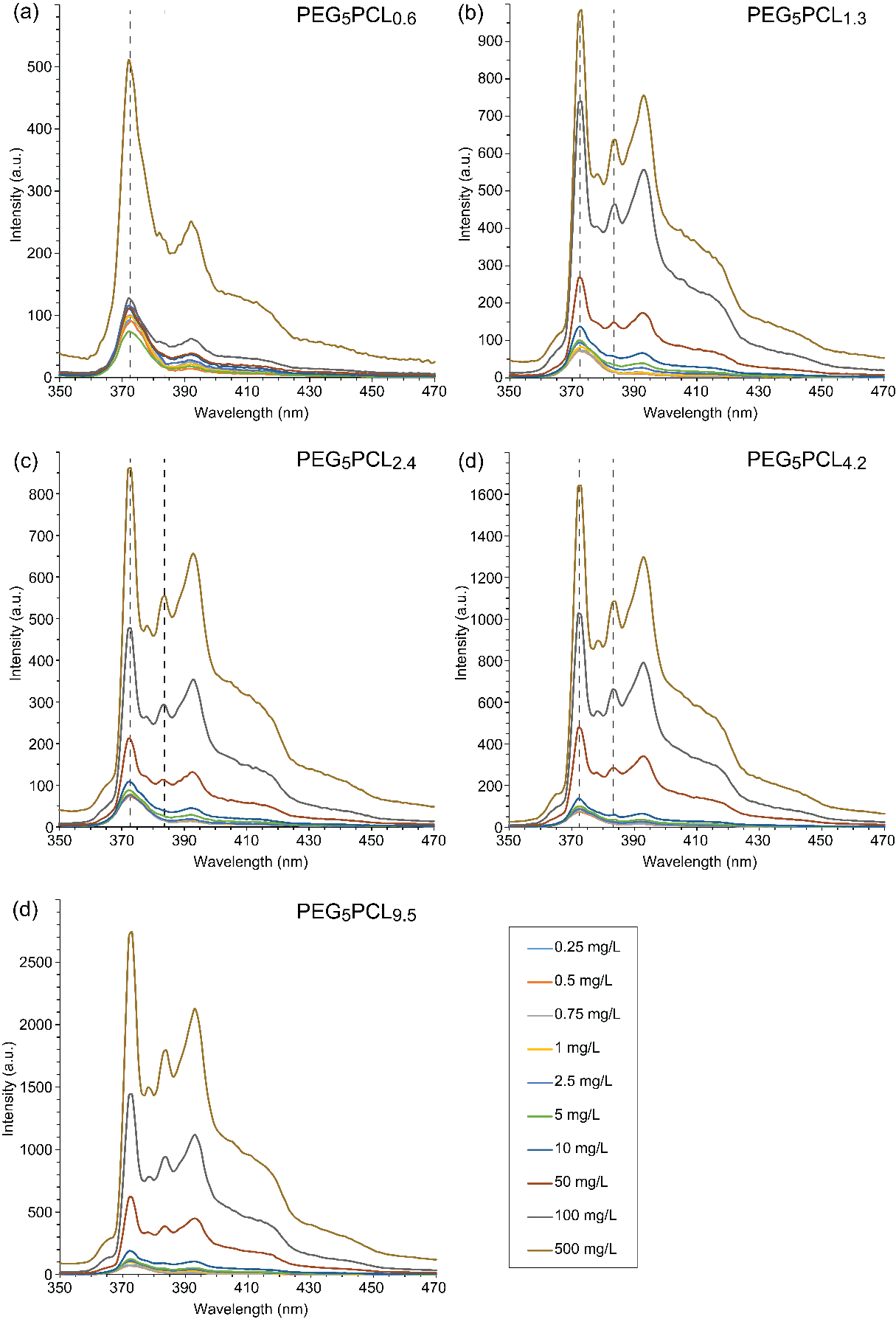
**

**Figure S2.** Pyrene (6 x 10^-7^ M) fluorescent emission spectra (λ_ex_ = 334 nm) for (a) PEG_5_PCL_0.6_, (b) PEG_5_PCL_1.3_, (c) PEG_5_PCL_2.4_, (d) PEG_5_PCL_4.2_ and (e) PEG_5_PCL_9.5_ copolymers at various copolymer concentrations.


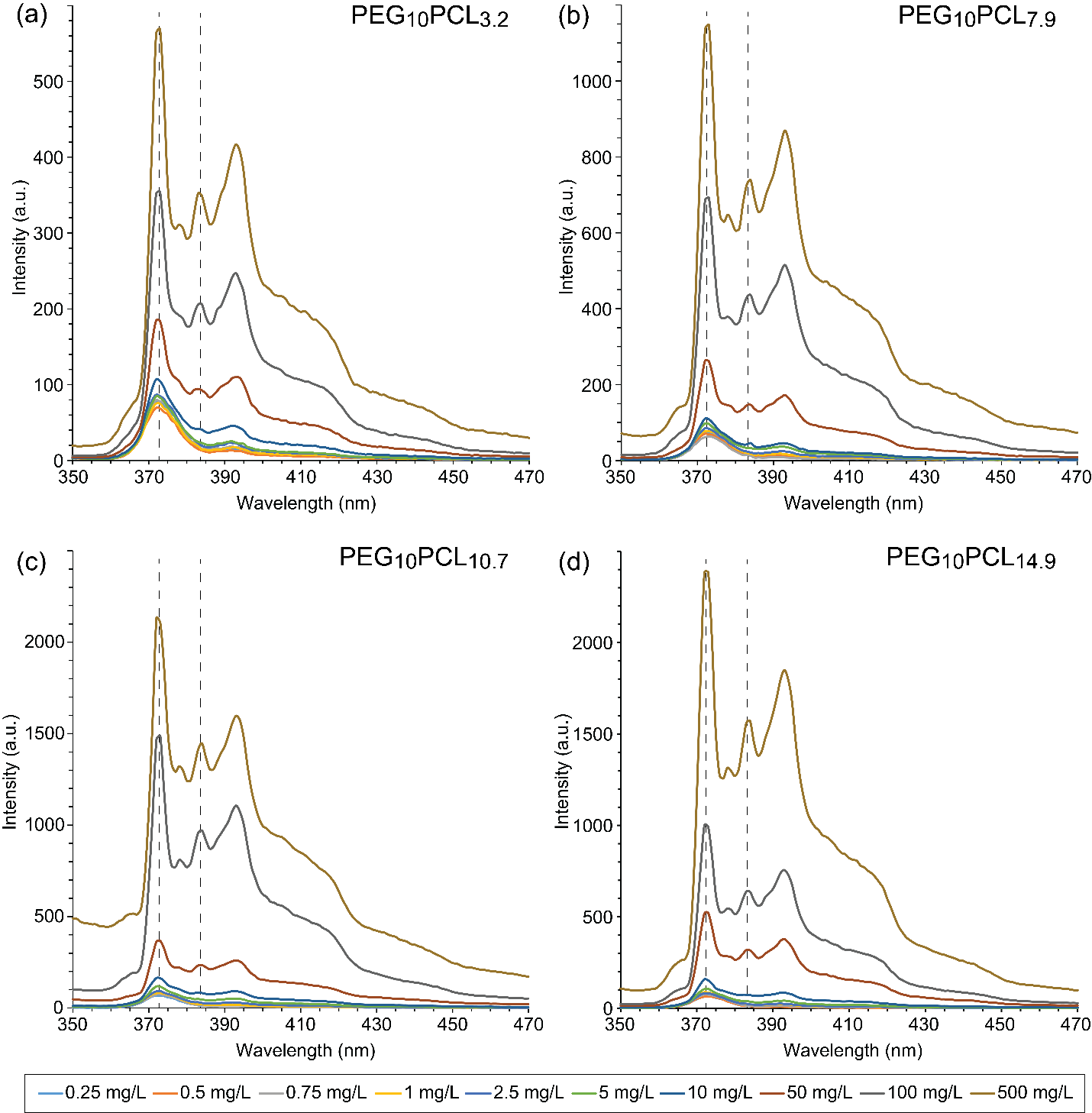


**Figure S3.** Pyrene (6 x 10^-7^ M) fluorescent emission spectra (λ_ex_ = 334 nm) for (a) PEG_10_PCL_3.2_, (b) PEG_10_PCL_7.9_, (c) PEG_10_PCL_10.7_ and (d) PEG_10_PCL_14.9_ copolymers at various copolymer concentrations.


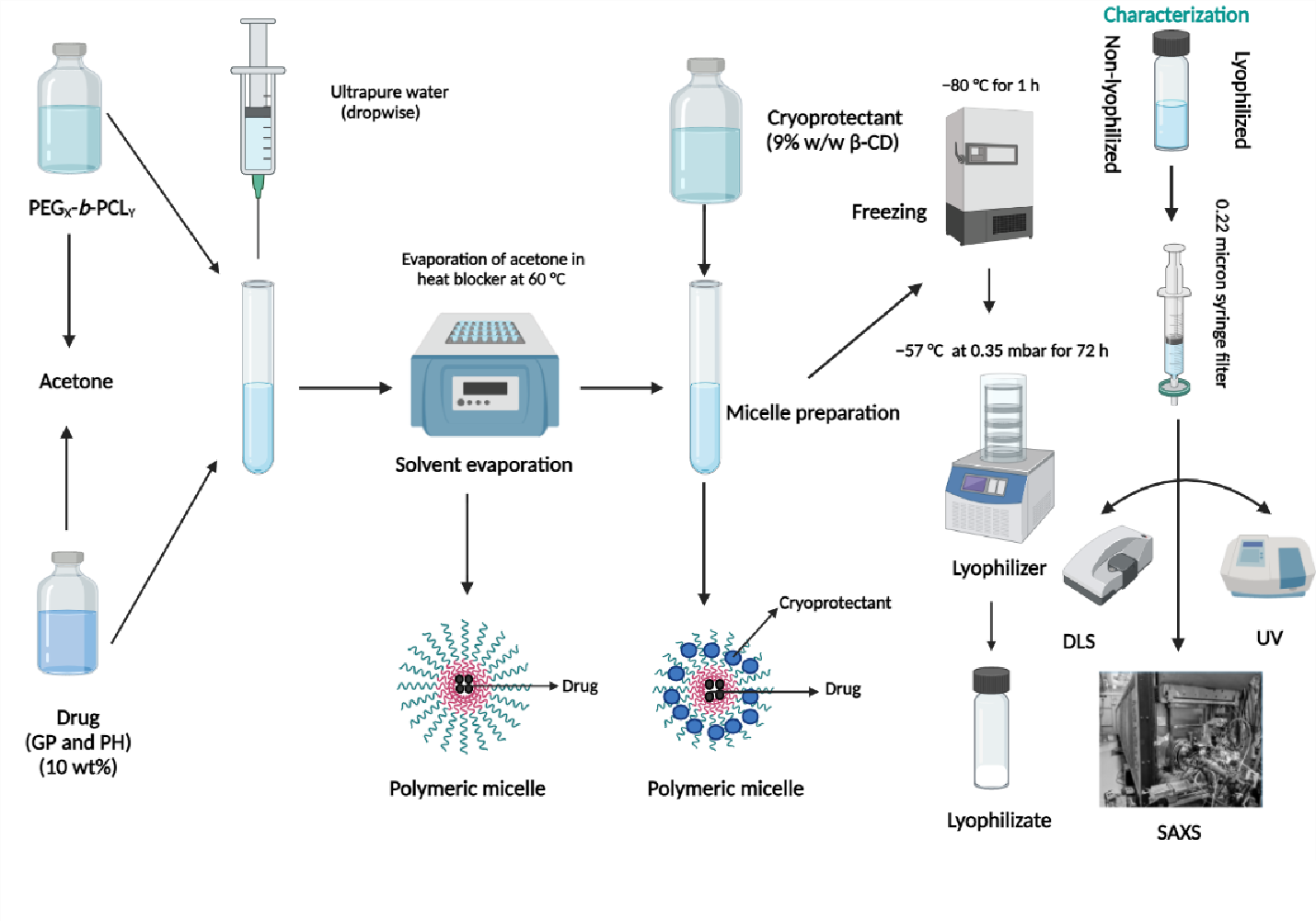


**Figure S4.** Illustration of preparation, lyophilization and characterisation of PEG_X_-*b*-PCL_Y_ micelles.

**Table S2.** Polydispersity index (PDI) of micelles before and after lyophilization/reconstitution in the absence and presence of β-CD (-/+CD) as cryoprotectant, as determined by dynamic light scattering DLS; values are expressed as mean ± standard deviation (n=3).

| Copolymer | Blank | | Gossypol (GP) | Phloretin (PH) | Before  freeze-drying  (-CD)  (PDI ± SD) | Before  freeze-drying  (+CD)  (PDI ± SD) | After  freeze-drying  (-CD)  (PDI ± SD) | After  freeze-drying  (+CD)  (PDI ± SD) |
| --- | --- | --- | --- | --- | --- | --- | --- | --- |
| PEG_2_PCL_0.4_ | √ | | - | - | 0.149 ± 0.010 | 0.156 ± 0.009 | 0.196 ± 0.010 | 0.185 ± 0.013 |
| PEG_2_PCL_1.1_ | √ | | - | - | 0.189 ± 0.011 | 0.245 ± 0.014 | 0.145 ± 0.048 | 0.149 ± 0.008 |
| PEG_2_PCL_1.1_ | - | | √ | - | 0.140 ± 0.004 | 0.179 ± 0.052 | 0.162 ± 0.025 | 0.145 ± 0.005 |
| PEG_2_PCL_1.1_ | | - | - | √ | 0.135 ± 0.023 | 0.158 ± 0.003 | 0.157 ± 0.009 | 0.126 ± 0.002 |
| PEG_2_PCL_1.8_ | | √ | - | - | 0.225 ± 0.006 | 0.252 ± 0.007 | 0.141 ± 0.001 | 0.228 ± 0.002 |
| PEG_2_PCL_1.8_ | | - | √ | - | 0.160 ± 0.025 | 0.187 ± 0.034 | 0.136 ± 0.008 | 0.132 ± 0.005 |
| PEG_2_PCL_1.8_ | | - | - | √ | 0.147 ± 0.011 | 0.178 ± 0.030 | 0.158 ± 0.008 | 0.184 ± 0.005 |
| PEG_2_PCL_4.0_ | | √ | - | - | 0.245 ± 0.030 | 0.195 ± 0.003 | 0.210 ± 0.011 | 0.186 ± 0.039 |
| PEG_5_PCL_0.6_ | | √ | - | - | 0.262 ± 0.007 | 0.242 ± 0.003 | 0.210 ± 0.016 | 0.177 ± 0.050 |
| PEG_5_PCL_1.3_ | | √ | - | - | 0.241 ± 0.008 | 0.245 ± 0.038 | 0.185 ± 0.016 | 0.188 ± 0.006 |
| PEG_5_PCL_1.3_ | | - | √ | - | 0.126 ± 0.010 | 0.185 ± 0.016 | 0.148 ± 0.006 | 0.147 ± 0.007 |
| PEG_5_PCL_1.3_ | | - | - | √ | 0.205 ± 0.003 | 0.257 ± 0.050 | 0.205 ± 0.003 | 0.246 ± 0.056 |
| PEG_5_PCL_2.4_ | | √ | - | - | 0.192 ± 0.008 | 0.145 ± 0.030 | 0.176 ± 0.035 | 0.163 ± 0.011 |
| PEG_5_PCL_2.4_ | | - | √ | - | 0.220 ± 0.060 | 0.201 ± 0.004 | 0.236 ± 00.17 | 0.158 ± 0.008 |
| PEG_5_PCL_2.4_ | | - | - | √ | 0.197 ± 0.002 | 0.139 ± 0.005 | 0.131 ± 0.002 | 0.159 ± 0.014 |
| PEG_5_PCL_4.2_ | | √ | - | - | 0.123 ± 0.006 | 0.132 ± 0.046 | 0.176 ± 0.006 | 0.138 ± 0.005 |
| PEG_5_PCL_4.2_ | | - | √ | - | 0.192 ± 0.004 | 0.175 ± 0.003 | 0.156 ± 0.019 | 0.158 ± 0.016 |
| PEG_5_PCL_4.2_ | | - | - | √ | 0.168 ± 0.028 | 0.085 ± 0.009 | 0.140 ± 0.040 | 0.137 ± 0.034 |
| PEG_5_PCL_9.5_ | | √ | - | - | 0.138 ± 0.004 | 0.145 ± 0.011 | 0.128 ± 0.011 | 0.116 ± 0.021 |
| PEG_5_PCL_9.5_ | | - | √ | - | 0.193 ± 0.002 | 0.197 ± 0.005 | 0.153 ± 0.011 | 0.156 ± 0.016 |
| PEG_5_PCL_9.5_ | | - | - | √ | 0.144 ± 0.013 | 0.127 ± 0.011 | 0.118 ± 0.019 | 0.168 ± 0.030 |
| PEG_10_PCL_3.2_ | | √ | - | - | 0.173 ± 0.004 | 0.186 ± 0.006 | 0.133 ± 0.002 | 0.186 ± 0.015 |
| PEG_10_PCL_7.9_ | | √ | - | - | 0.170 ± 0.009 | 0.176 ± 0.003 | 0.195 ± 0.020 | 0.172 ± 0.009 |
| PEG_10_PCL_7.9_ | | - | √ | - | 0.183 ± 0.013 | 0.172 ± 0.007 | 0.243 ± 0.011 | 0.232 ± 0.009 |
| PEG_10_PCL_7.9_ | | - | - | √ | 0.149 ± 0.014 | 0.164 ± 0.006 | 0.182 ± 0.006 | 0.216 ± 0.024 |
| PEG_10_PCL_10.7_ | | √ | - | - | 0.146 ± 0.017 | 0.149 ± 0.007 | 0.119 ±0.006 | 0.125 ± 0.002 |
| PEG_10_PCL_10.7_ | | - | √ | - | 0.138 ± 0.006 | 0.153 ± 0.008 | 0.186 ± 0.006 | 0.187 ± 0.013 |
| PEG_10_PCL_10.7_ | | - | - | √ | 0.116 ± 0.007 | 0.225 ± 0.042 | 0.230 ± 0.005 | 0.191 ± 0.017 |
| PEG_10_PCL_14.9_ | | √ | - | - | 0.160 ± 0.008 | 0.160 ± 0.011 | 0.128 ±0.013 | 0.187 ± 0.025 |


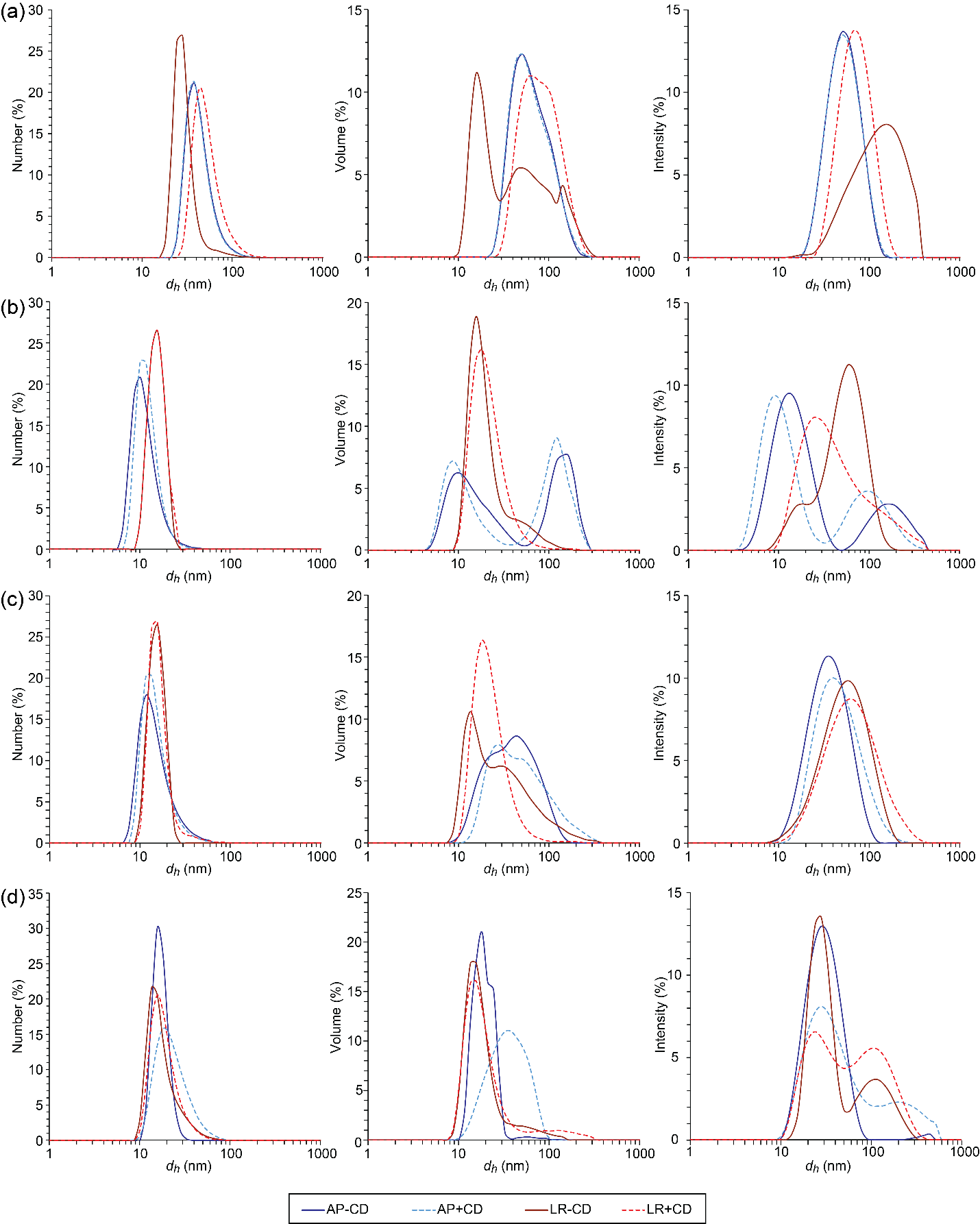


**Figure S5.** Number, volume and intensity particle size distributions for (a) PEG_2_PCL_0.4_, (b) PEG_2_PCL_1.1_, (c) PEG_2_PCL_1.8_ and (d) PEG_2_PCL_4.0_ micelles as prepared (AP) and after lyophilization/reconstitution (LR) in the absence and presence of β-CD (-/+CD).


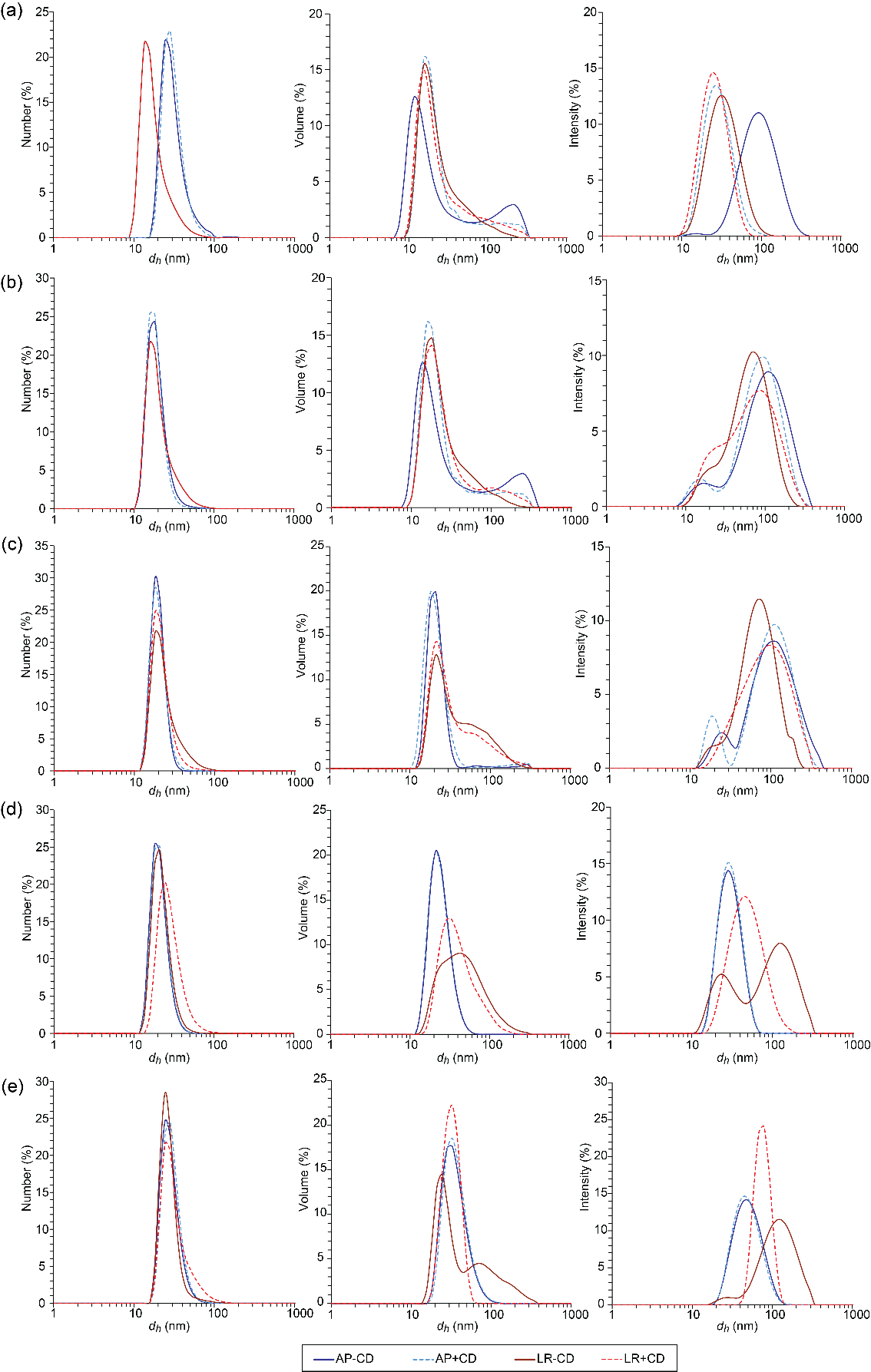


**Figure S6.** Number, volume and intensity particle size distributions for (a) PEG_5_PCL_0.6_, (b) PEG_5_PCL_1.3_, (c) PEG_5_PCL_2.4_, (d) PEG_5_PCL_4.2_ and (e) PEG_5_PCL_9.5_ micelles as prepared (AP) and after lyophilization/reconstitution (LR) in the absence and presence of β-CD (-/+CD).


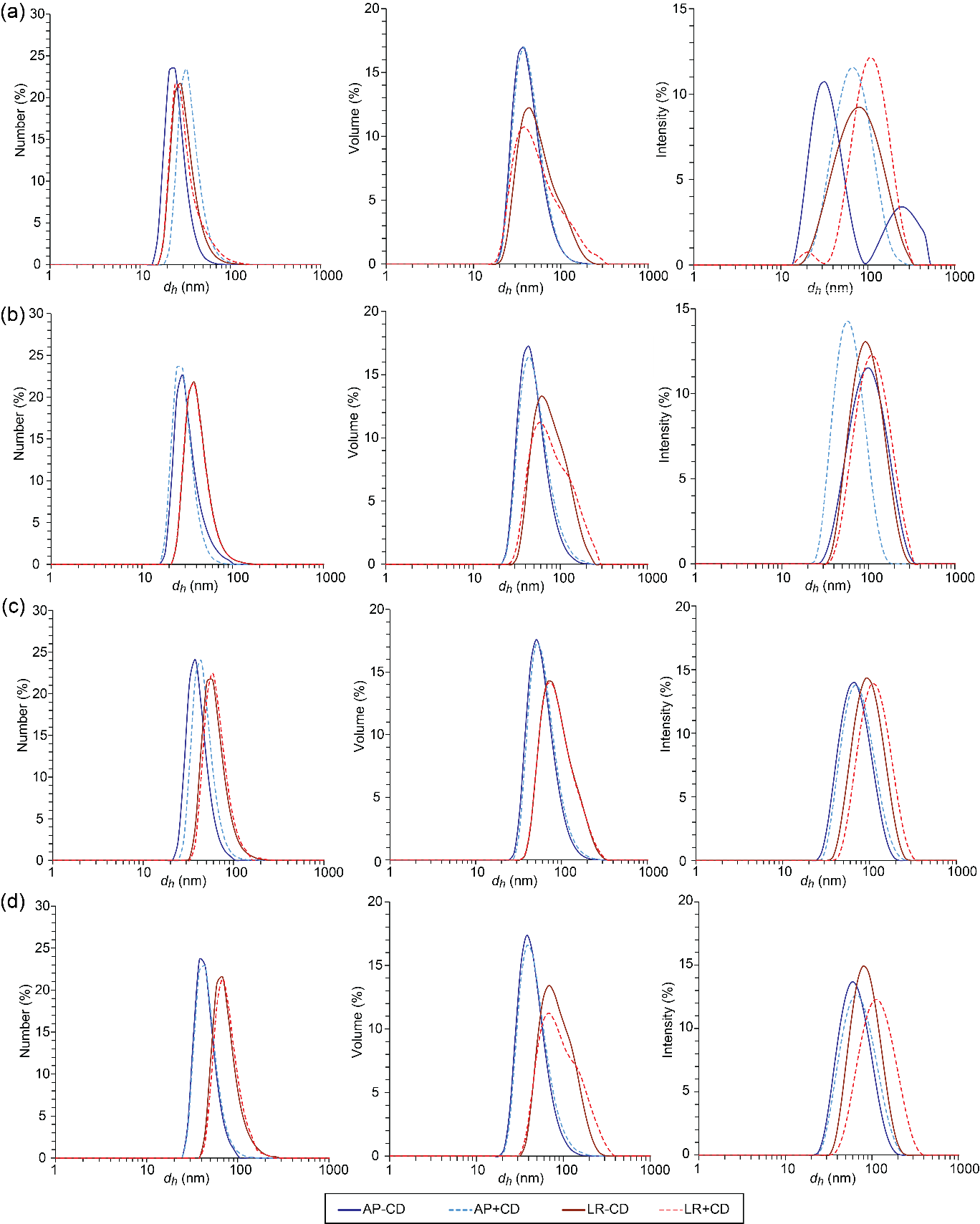


**Figure S7.** Number, volume and intensity particle size distributions for (a) PEG_10_PCL_3.2_, (b) PEG_10_PCL_7.9_, (c) PEG_10_PCL_10.7_ and (d) PEG_10_PCL_14.9_ micelles as prepared (AP) and after lyophilization/reconstitution (LR) in the absence and presence of β-CD (-/+CD).


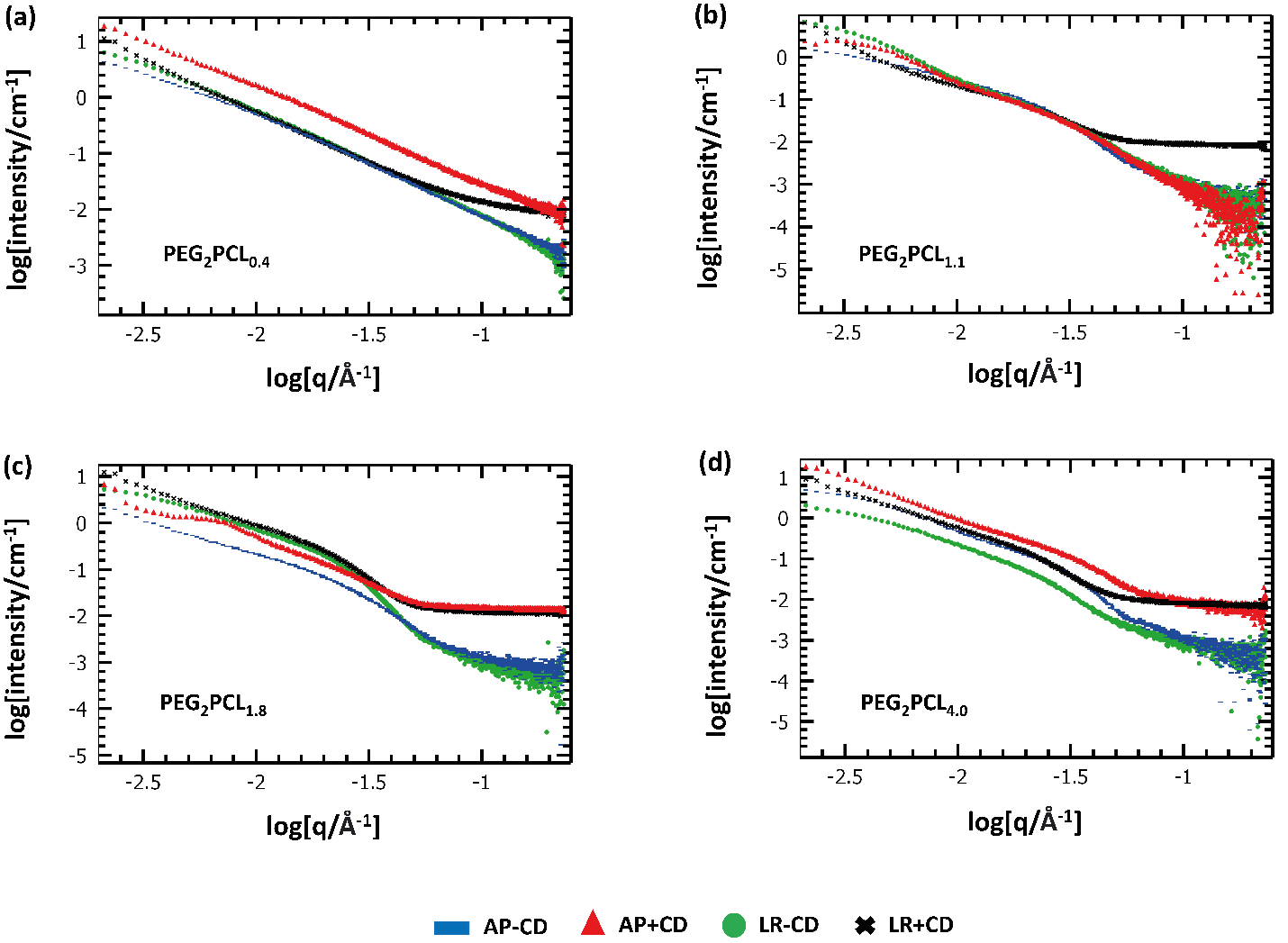


**Figure S8.** SAXS profiles for (a) PEG_2_PCL_0.4_, (b) PEG_2_PCL_1.1_, (c) PEG_2_PCL_1.8_ and (d) PEG_2_PCL_4.0_ micelles/aggregates as prepared (AP) and after lyophilization/reconstitution (LR) in the absence and presence of β-CD (-/+CD).


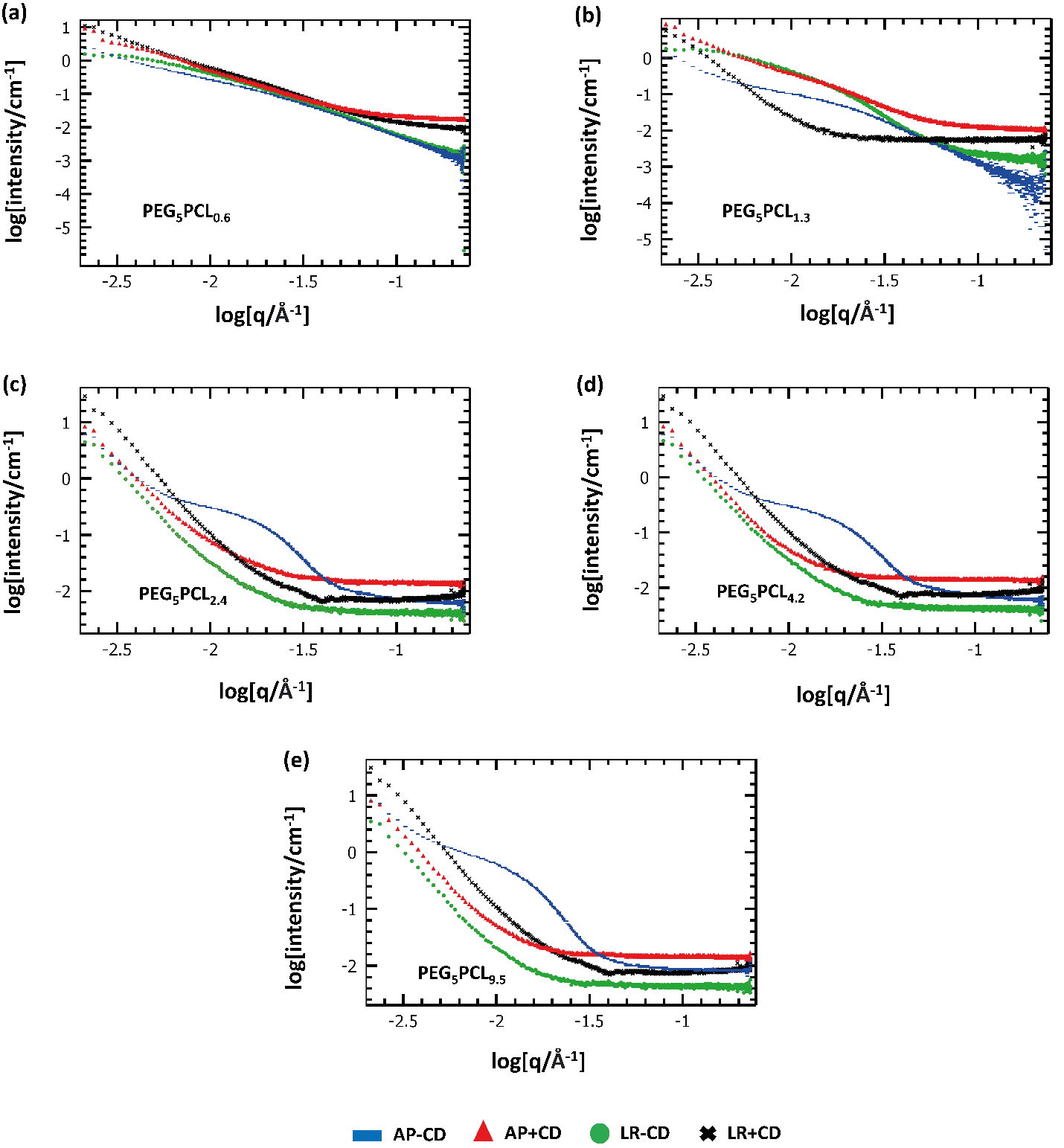


**Figure S9.** SAXS profiles for (a) PEG_5_PCL_0.6_, (b) PEG_5_PCL_1.3_, (c) PEG_5_PCL_2.4_, (d) PEG_5_PCL_4.2_ and (e) PEG_5_PCL_9.5_ micelles/aggregates as prepared (AP) and after lyophilization/reconstitution (LR) in the absence and presence of β-CD (-/+CD). Background multiplication factor (BMF) for PEG_5_PCL_1.3_ LR+CD, PEG_5_PCL_2.4_ LR+CD, PEG_5_PCL_4.2_ LR+CD and PEG_5_PCL_9.5_ LR+CD was 0.72, 0.55, 0.55 and 0.53, respectively.


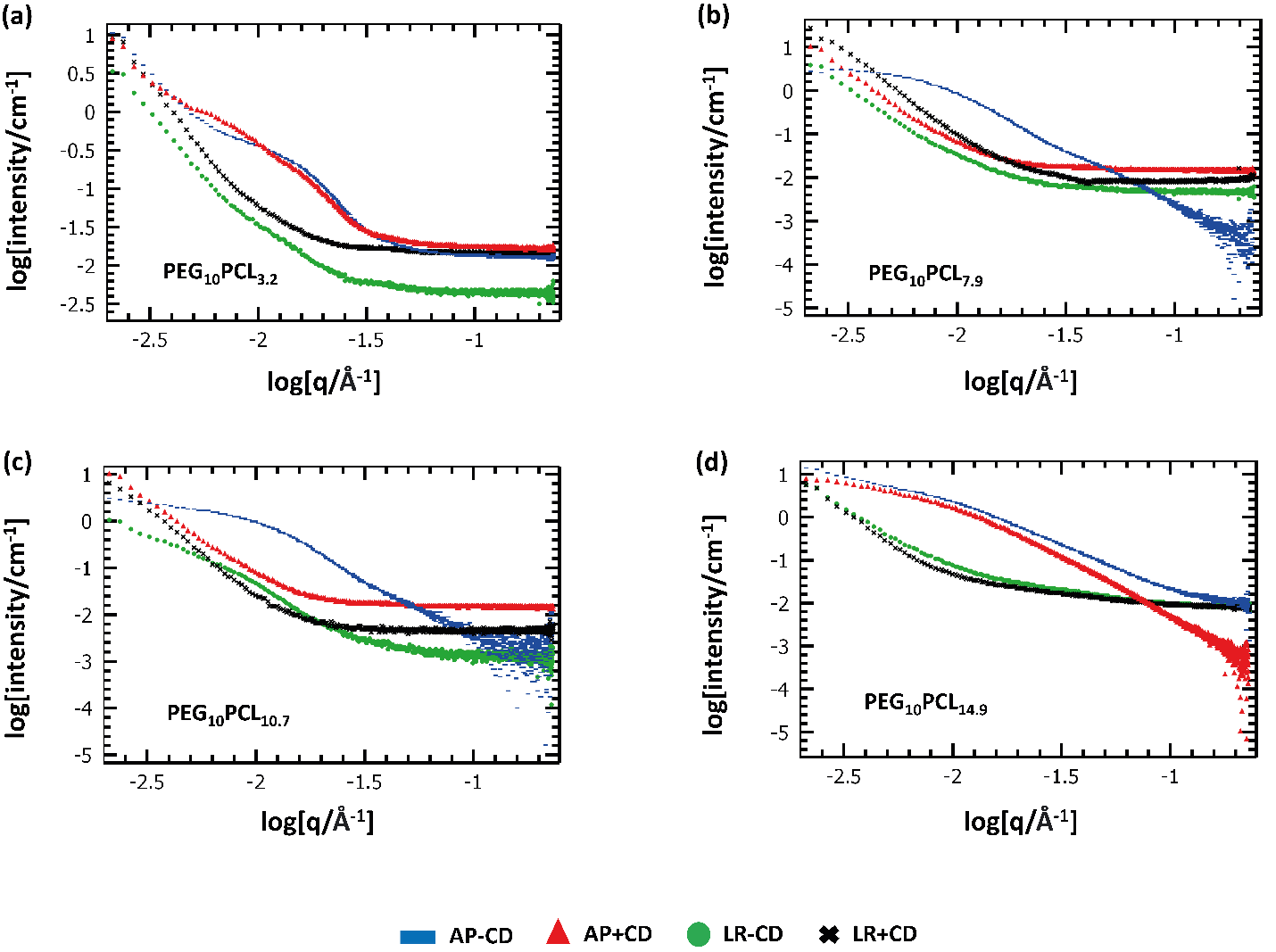


**Figure S10.** SAXS profiles for (a) PEG_10_PCL_3.2_, (b) PEG_10_PCL_7.9_, (c) PEG_10_PCL_10.7_ and (d) PEG_10_PCL_14.9_ micelles/aggregates as prepared (AP) and after lyophilization/reconstitution (LR) in the absence and presence of β-CD (-/+CD) (BMF for PEG_10_PCL_3.2_ LR+CD, PEG_10_PCL_7.9_ LR+CD and PEG_10_PCL_10.7_ LR+CD was 0.45, 0.55 and 0.53, respectively).


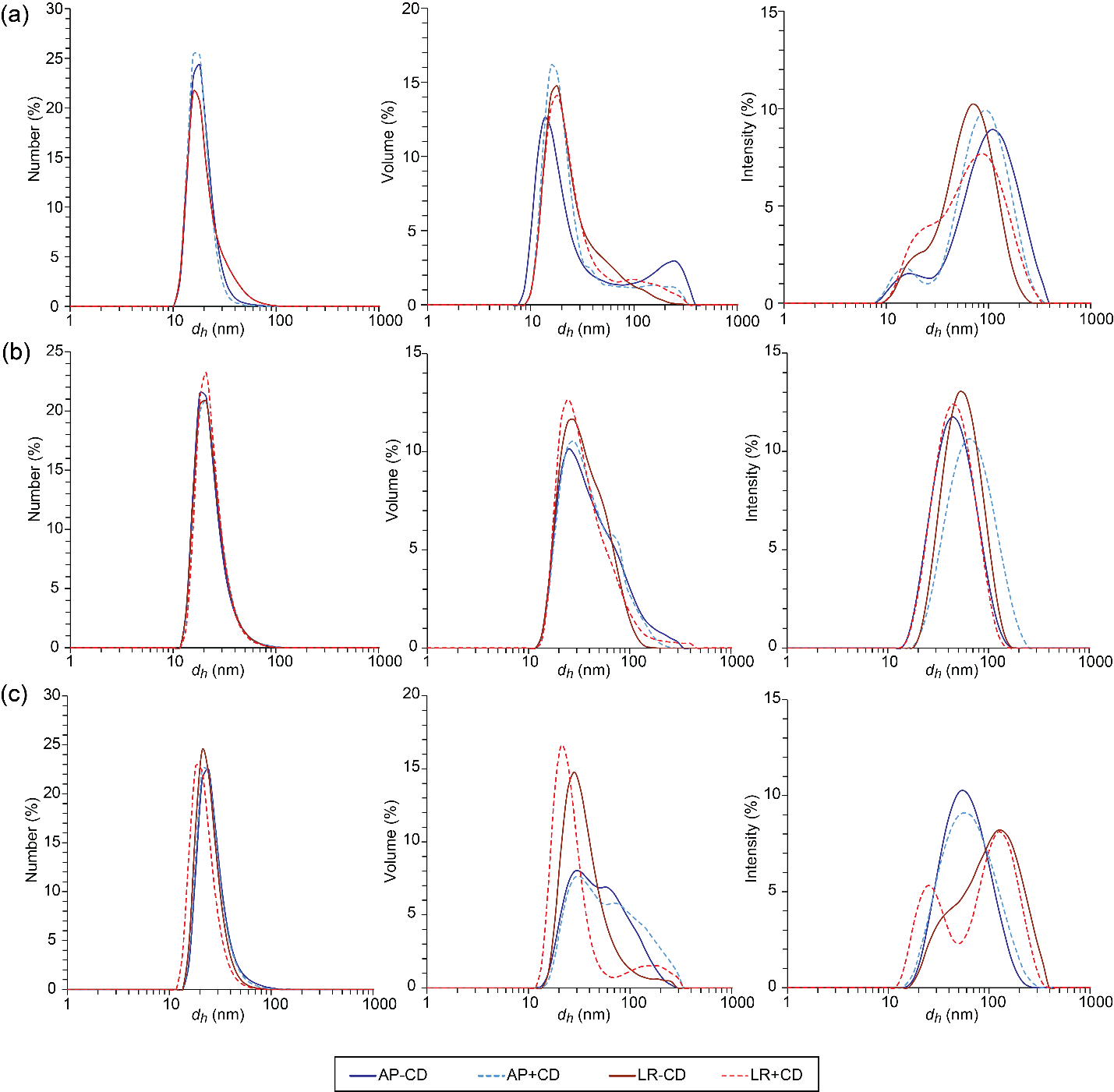


**Figure S11.** Number, volume and intensity particle size distributions for PEG_5_PCL_1.3_ (a) blank micelles and (b) gossypol (GP) and (c) phloretin (PH) loaded micelles as prepared (AP) and after lyophilization/reconstitution (LR) in the absence and presence of β-CD (-/+CD).


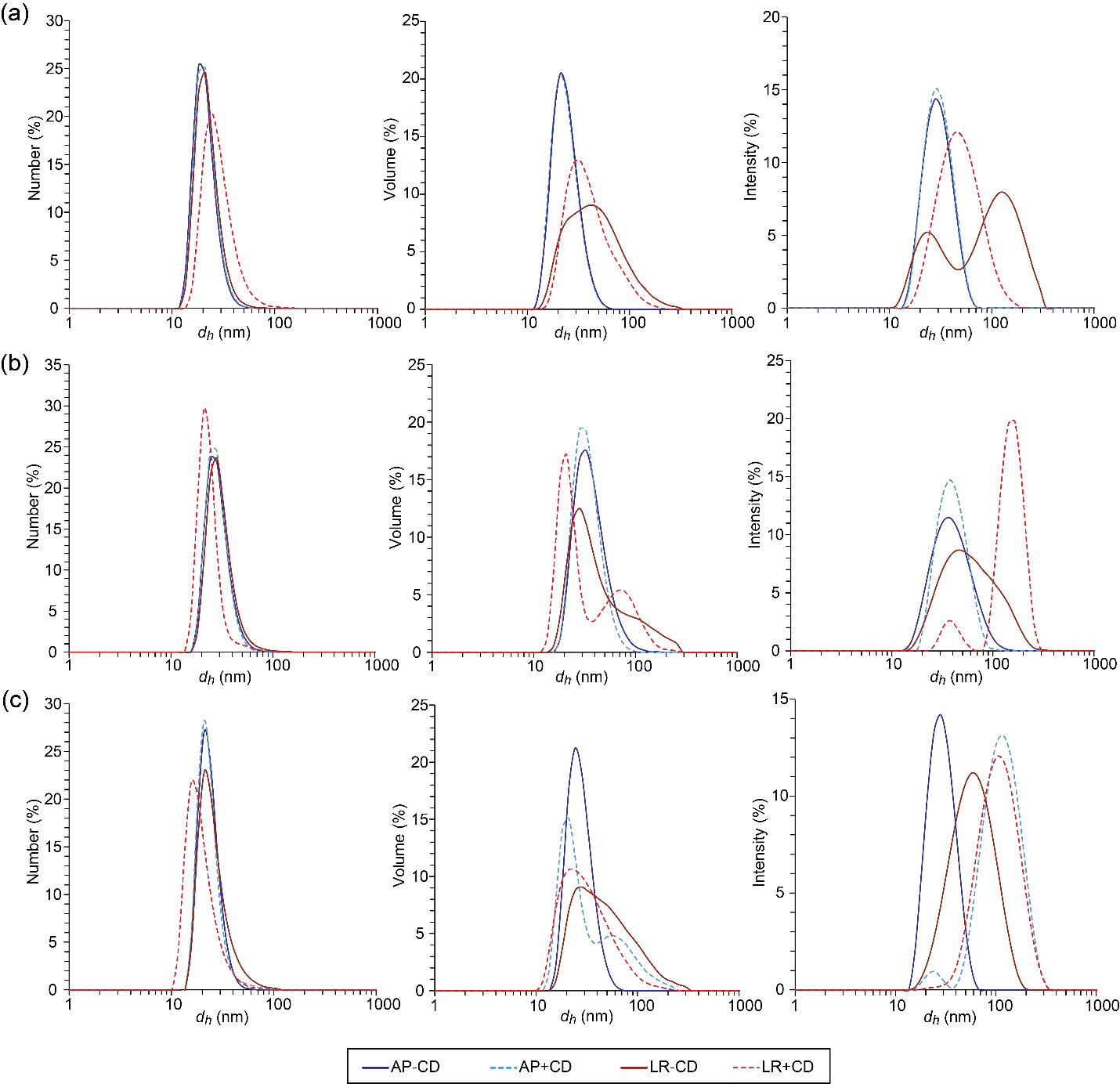


**Figure S12.** Number, volume and intensity particle size distributions for PEG_5_PCL_2.4_ (a) blank micelles and (b) gossypol (GP) and (c) phloretin (PH) loaded micelles as prepared (AP) and after lyophilization/reconstitution (LR) in the absence and presence of β-CD (-/+CD).


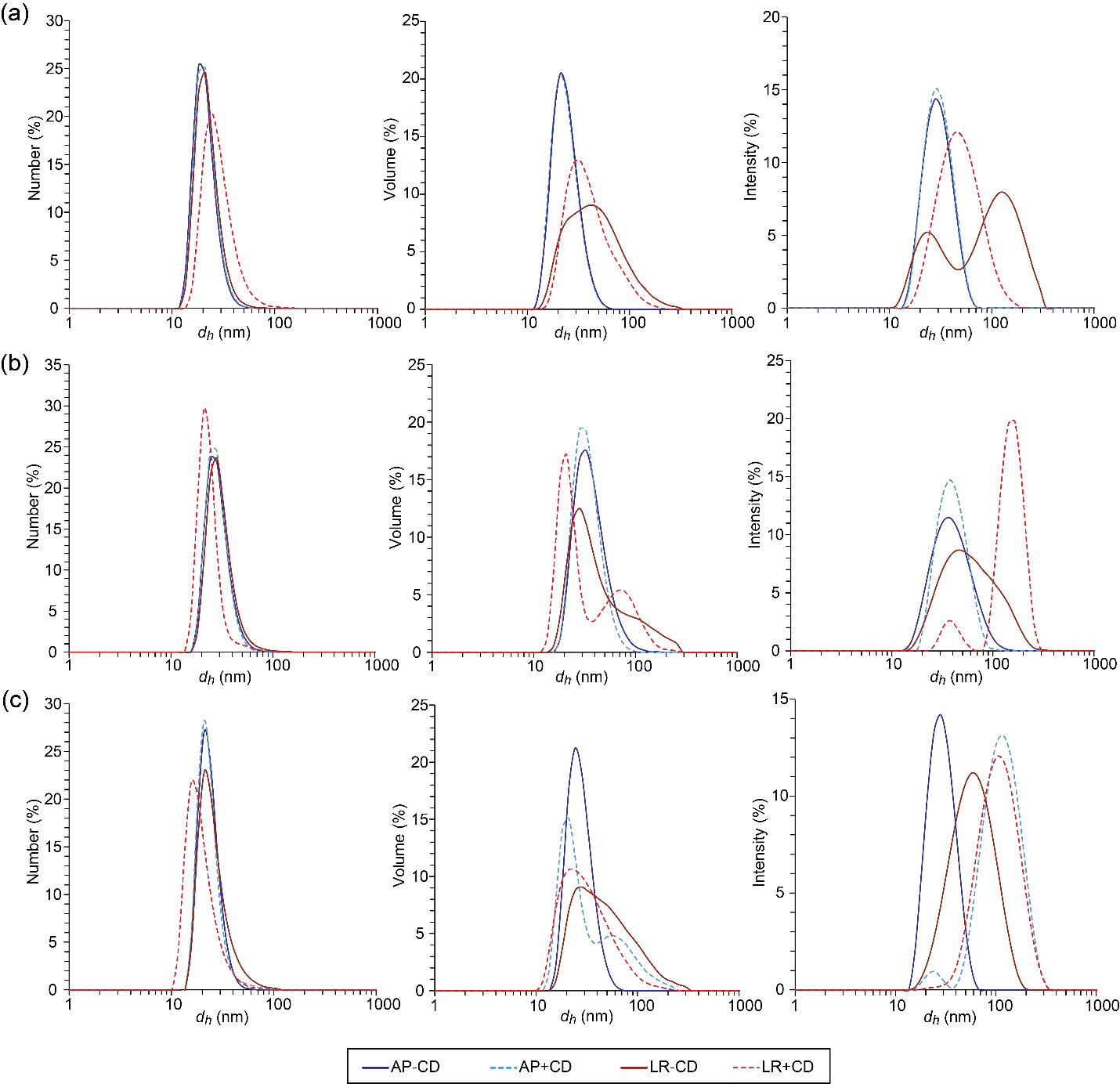


**Figure S13.** Number, volume and intensity particle size distributions for PEG_5_PCL_4.2_ (a) blank micelles and (b) gossypol (GP) and (c) phloretin (PH) loaded micelles as prepared (AP) and after lyophilization/reconstitution (LR) in the absence and presence of β-CD (-/+CD).


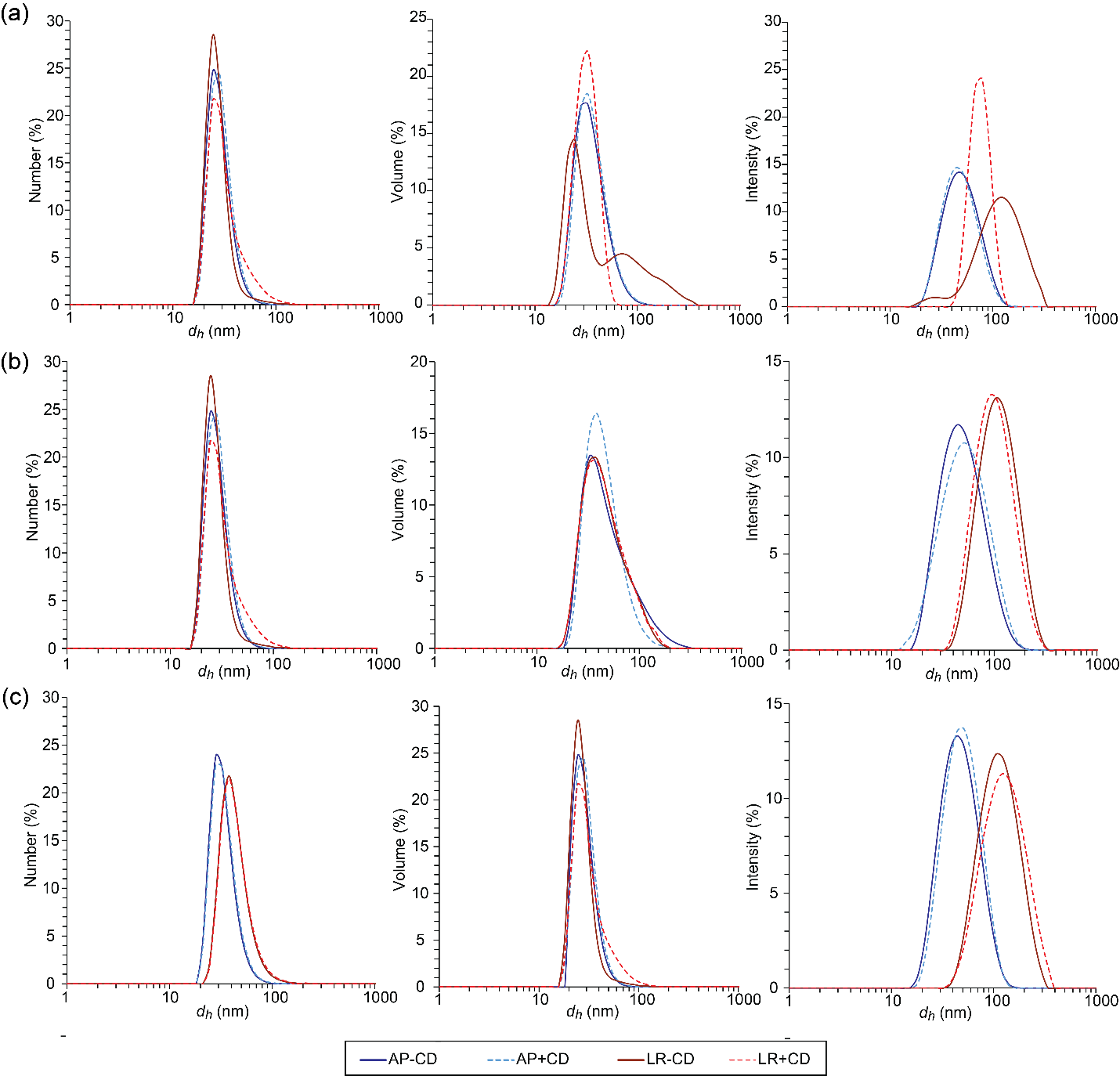


**Figure S14.** Number, volume and intensity particle size distributions for PEG_5_PCL_9.5_ (a) blank micelles and (b) gossypol (GP) and (c) phloretin (PH) loaded micelles as prepared (AP) and after lyophilization/reconstitution (LR) in the absence and presence of β-CD (-/+CD).


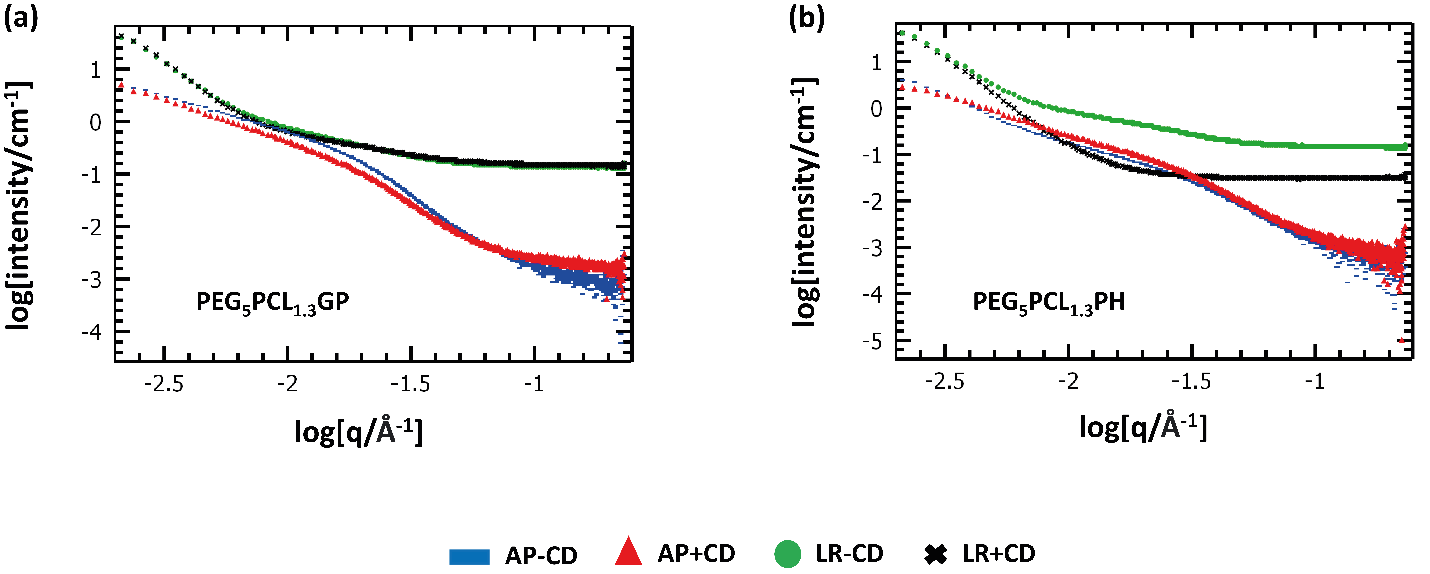


**Figure S15.** SAXS profiles for (a) gossypol and (b) phloretin loaded PEG_5_PCL_1.3_ micelles as prepared (AP) and after lyophilization/reconstitution (LR) in the absence and presence of β-CD (-/+CD).


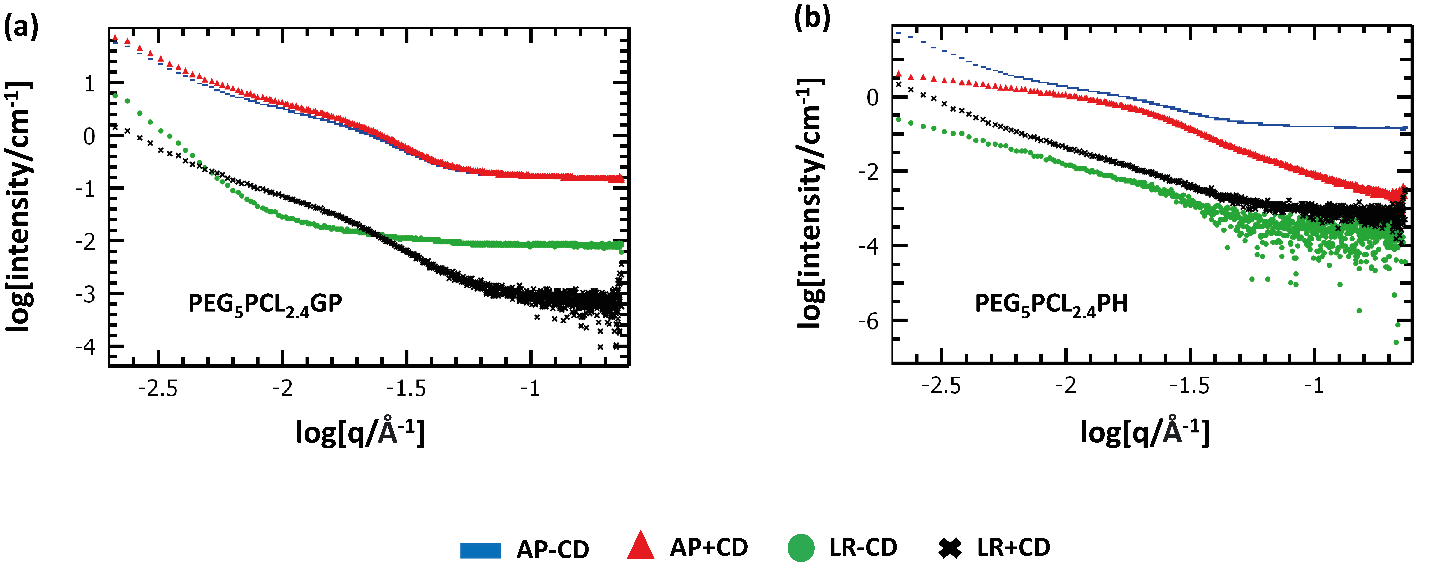


**Figure S16.** SAXS profiles for (a) gossypol and (b) phloretin loaded PEG_5_PCL_2.4_ micelles as prepared (AP) and after lyophilization/reconstitution (LR) in the absence and presence of β-CD (-/+CD).


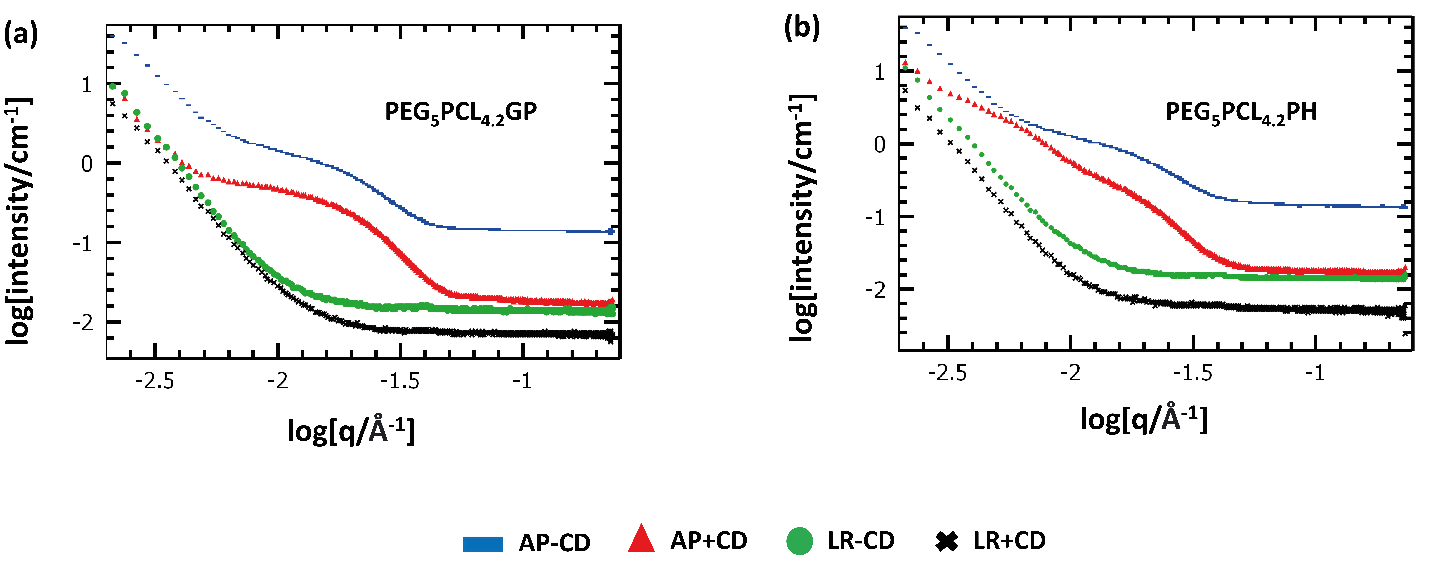


**Figure S17.** SAXS profiles for (a) gossypol and (b) phloretin loaded PEG_5_PCL_4.2_ micelles as prepared (AP) and after lyophilization/reconstitution (LR) in the absence and presence of β-CD (-/+CD) (BMF for PEG_5_PCL_4.2_ GP LR+CD and PEG_5_PCL_4.2_ PH LR+CD was 0.65 and 0.91, respectively).


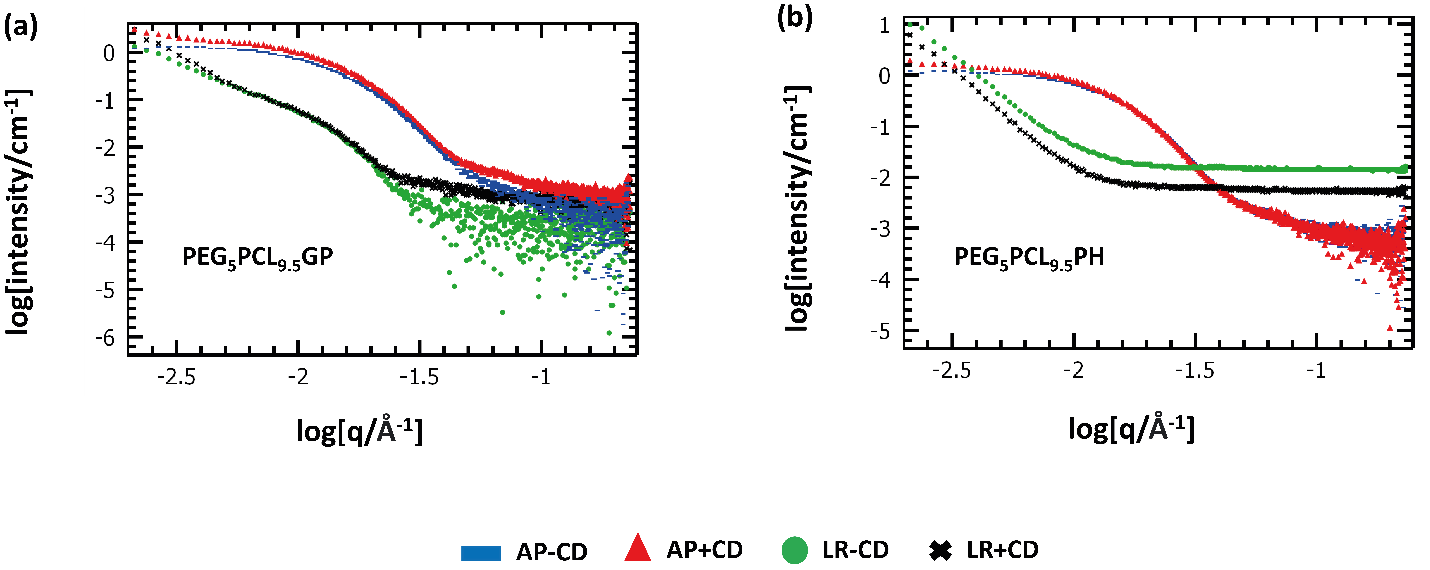


**Figure S18.** SAXS profiles for (a) gossypol and (b) phloretin loaded PEG_5_PCL_9.5_ micelles as prepared (AP) and after lyophilization/reconstitution (LR) in the absence and presence of β-CD (-/+CD) (BMF for PEG_5_PCL_9.5_ PH LR+CD was 0.91).


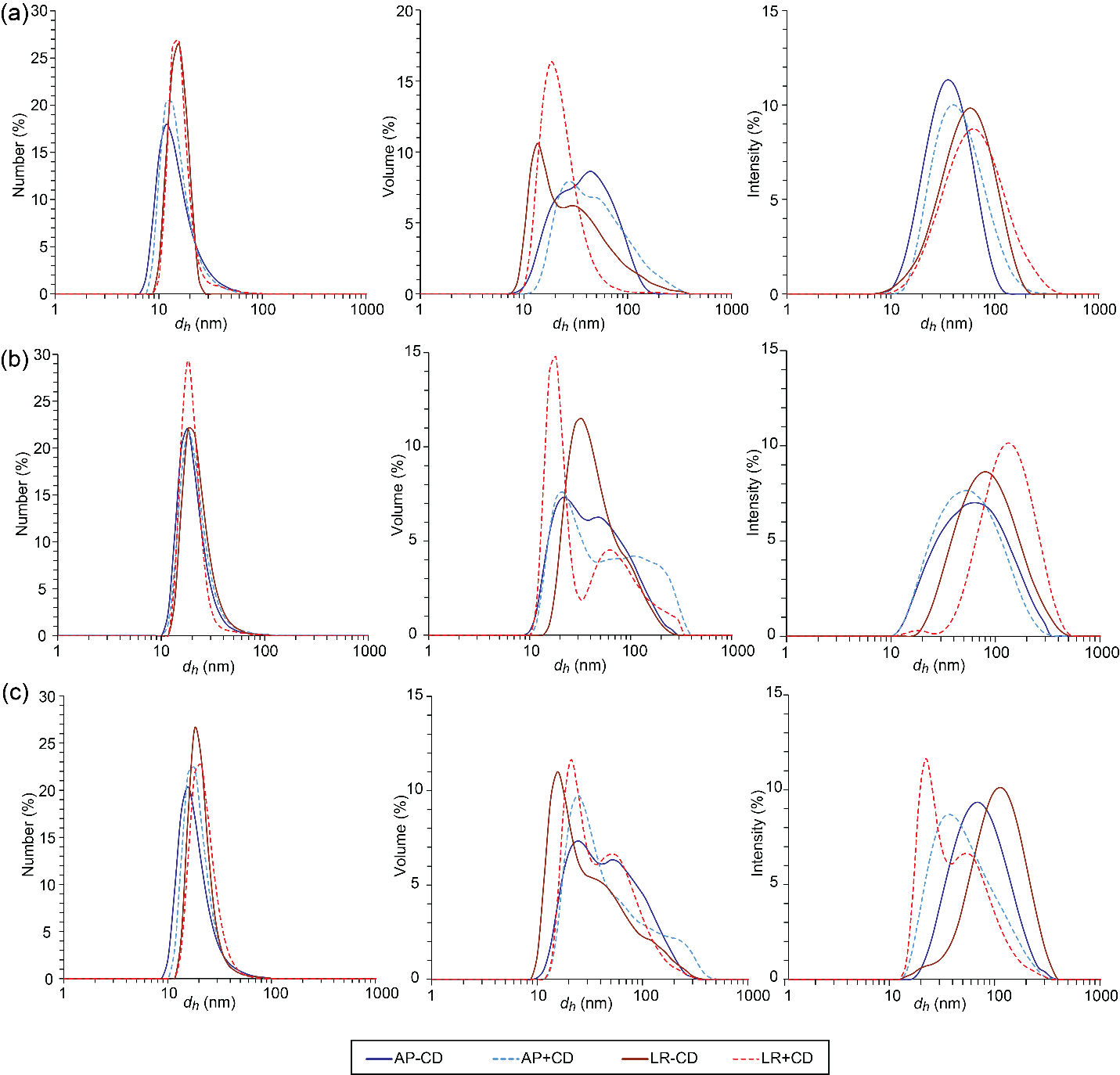


**Figure S19.** Number, volume and intensity particle size distributions for PEG_2_PCL_1.8_ (a) blank micelles and (b) gossypol (GP) and (c) phloretin (PH) loaded micelles as prepared (AP) and after lyophilization/reconstitution (LR) in the absence and presence of β-CD (-/+CD).


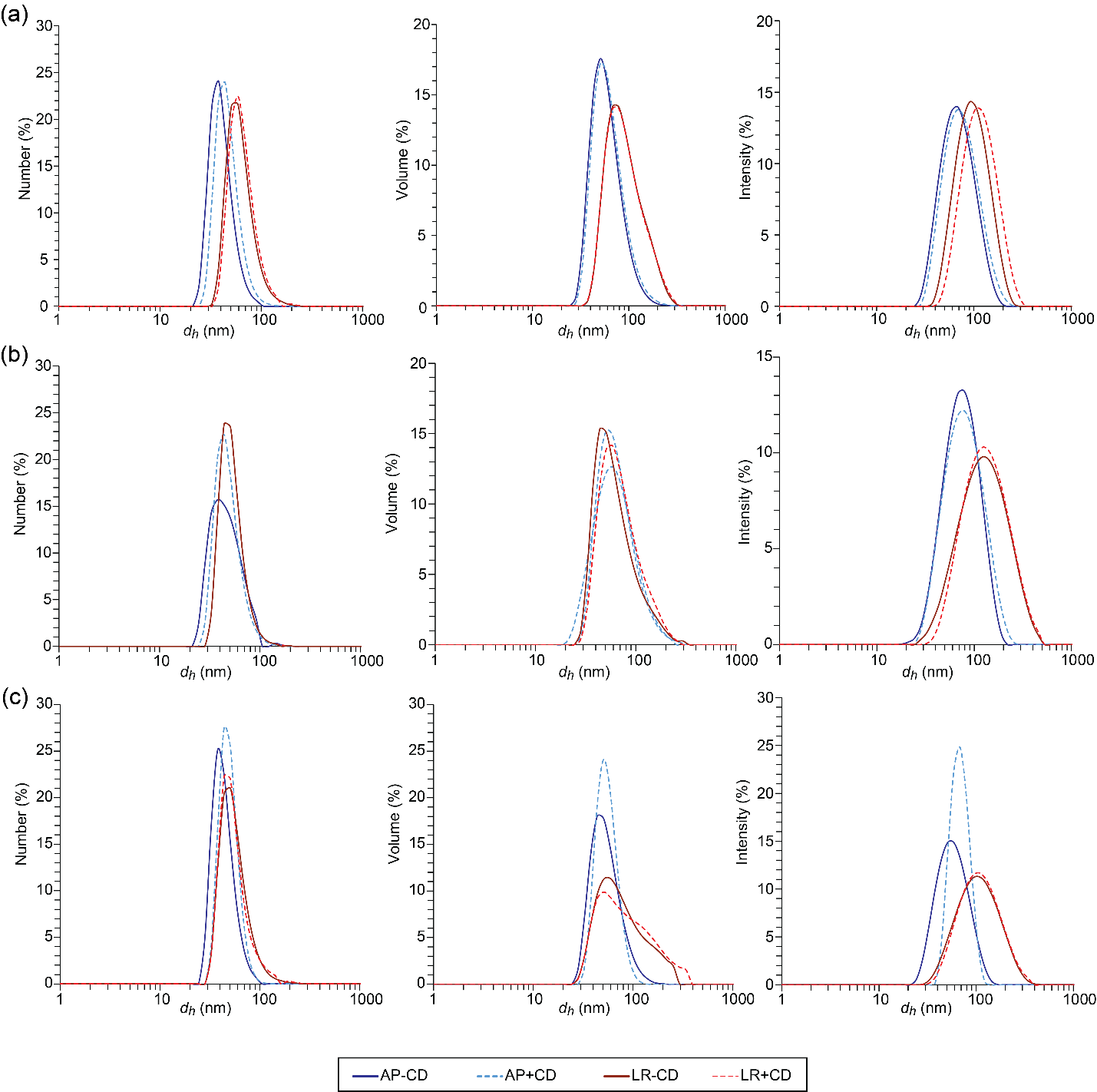


**Figure S20.** Number, volume and intensity particle size distributions for PEG_10_PCL_10.7_ (a) blank micelles and (b) gossypol (GP) and (c) phloretin (PH) loaded micelles as prepared (AP) and after lyophilization/reconstitution (LR) in the absence and presence of β-CD (-/+CD).


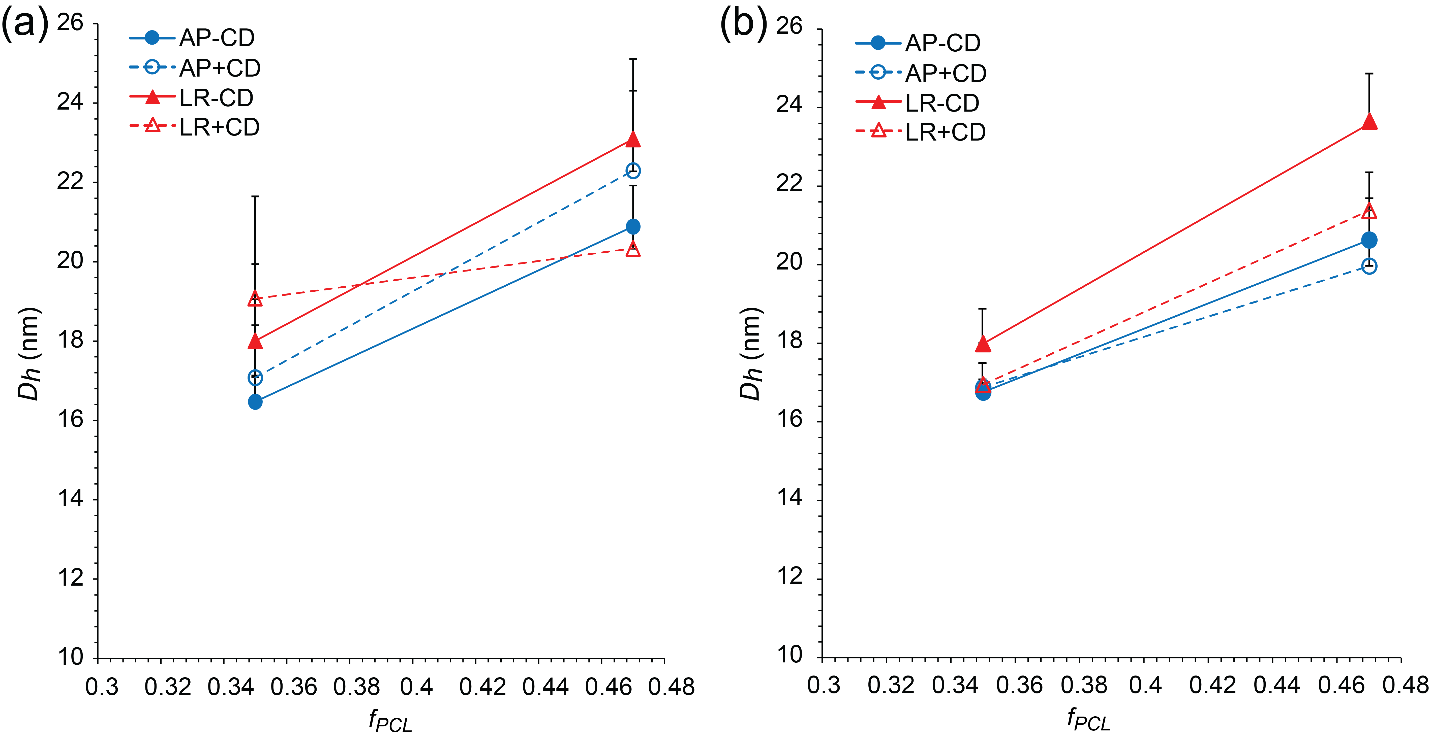


**Figure S21.** Hydrodynamic diameter (*D_h_*) versus weight fraction of PCL (*f_PCL_*) as determined from number particle size distributions (PSDs) for (a) gossypol (GP) and (b) phloretin (PH) loaded PEG_2_PCL_1.8_ micelles as prepared (AP) and after lyophilization/reconstitution (LR) and in the absence and presence of β-CD (-/+CD). All values are reported as the mean + std. dev. (n = 3).


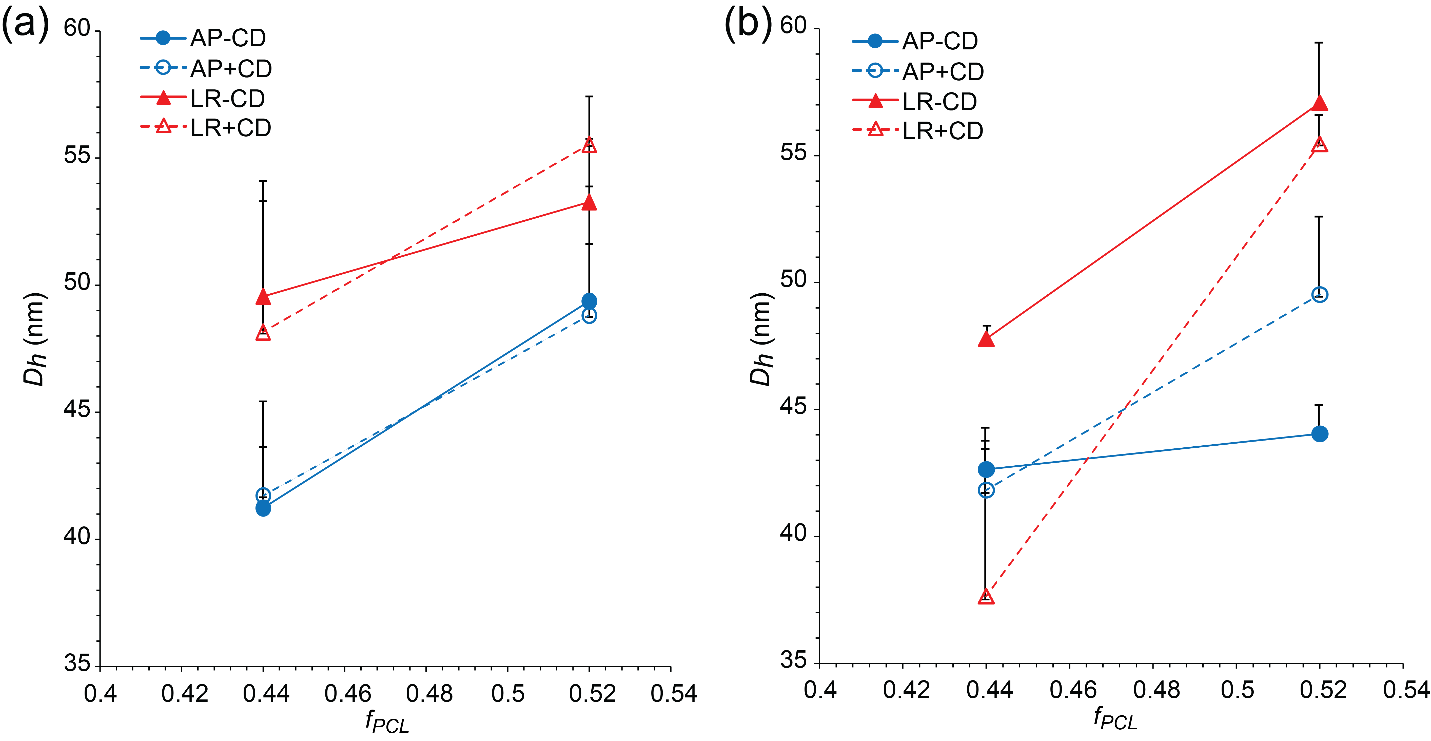


**Figure S22.** *D_h_* versus *f_PCL_* as determined from number PSDs for (a) gossypol (GP) and (b) phloretin (PH) loaded PEG_10_PCL_10.7_ micelles as prepared (AP) and after lyophilization/reconstitution (LR) and in the absence and presence of β-CD (-/+CD). All values are reported as the mean + std. dev. (n = 3).


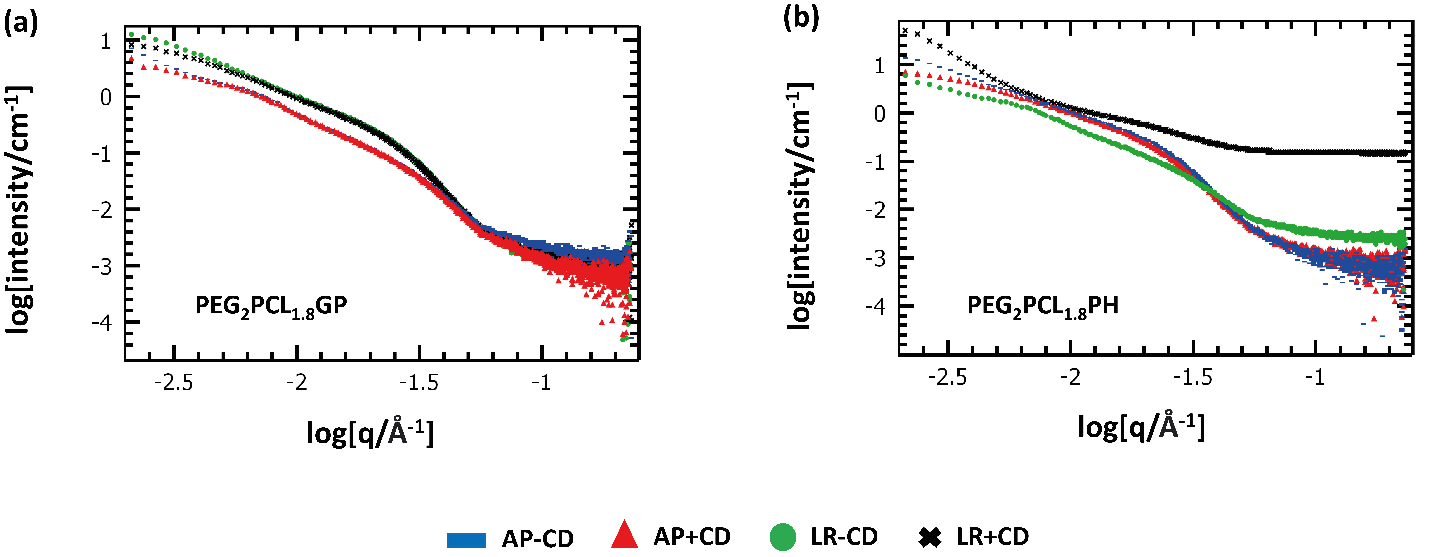


**Figure S23.** SAXS profiles for (a) gossypol and (b) phloretin loaded PEG_2_PCL_1.8_ micelles as prepared (AP) and after lyophilization/reconstitution (LR) in the absence and presence of β-CD (-/+CD).


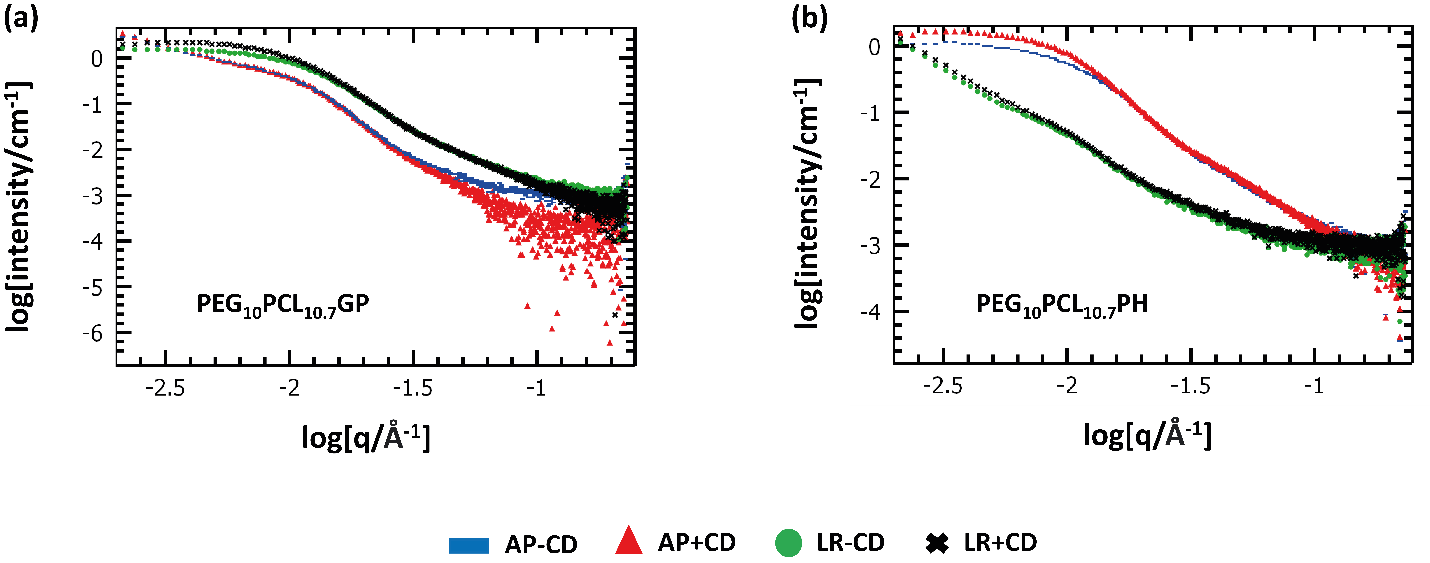


**Figure S24.** SAXS profiles for (a) gossypol and (b) phloretin loaded PEG_10_PCL_10.7_ micelles as prepared (AP) and after lyophilization/reconstitution (LR) in the absence and presence of β-CD (-/+CD).
