# Supplementary material for: Influence of Lyophilization and Cryoprotection on the Stability and Morphology of Drug-Loaded Poly(ethylene glycol-b-ε-caprolactone) Micelles
Source: Polymers (Basel). 2023 Apr 21;15(8):1974. doi: 10.3390/polym15081974 (PMC10146133; doi:10.3390/polym15081974)
Supplement: Supplementary file 1 [file polymers-15-01974-s001.zip › polymers-2306443-supplementary.pdf]

polymers

Supplementary Information

Influence of Lyophilization and Cryoprotection on the Stability and Morphology of Drug-Loaded Poly(Ethylene Glycol-*b*-ε-caprolactone) Micelles

Table S1. Concentration and volume of stock solutions combined to prepare copolymer solutions in ultrapure water for the determination of the CAC of PEG-*b*-PCL block copolymer micelles.

Conc. of copolymer in PBS (mg/L)[a]	Volume of pyrene stock solution (μL)[b]	Conc. of copolymer stock solution (mg/L)[c]	Volume of copolymer stock solution (μL)	Volume of ultrapure water added (μL)
500	20	2000	500	2000
100	20	2000	100	2000
50.0	20	1000	100	2000
10.0	20	1000	20	2000
5.00	20	1000	10	2000
2.50	20	100	50	2000
1.00	20	100	20	2000
0.75	20	100	15	2000
0.50	20	100	10	2000
0.25	20	10	50	2000

[a] Copolymer solutions were prepared in high purity water *via* the solvent evaporation approach with a constant pyrene concentration of 6×10^{-7} M. [b] A 6×10^{-5} M pyrene stock solution in acetone was used to prepare all solutions. [c] All stock solutions were prepared in acetone.

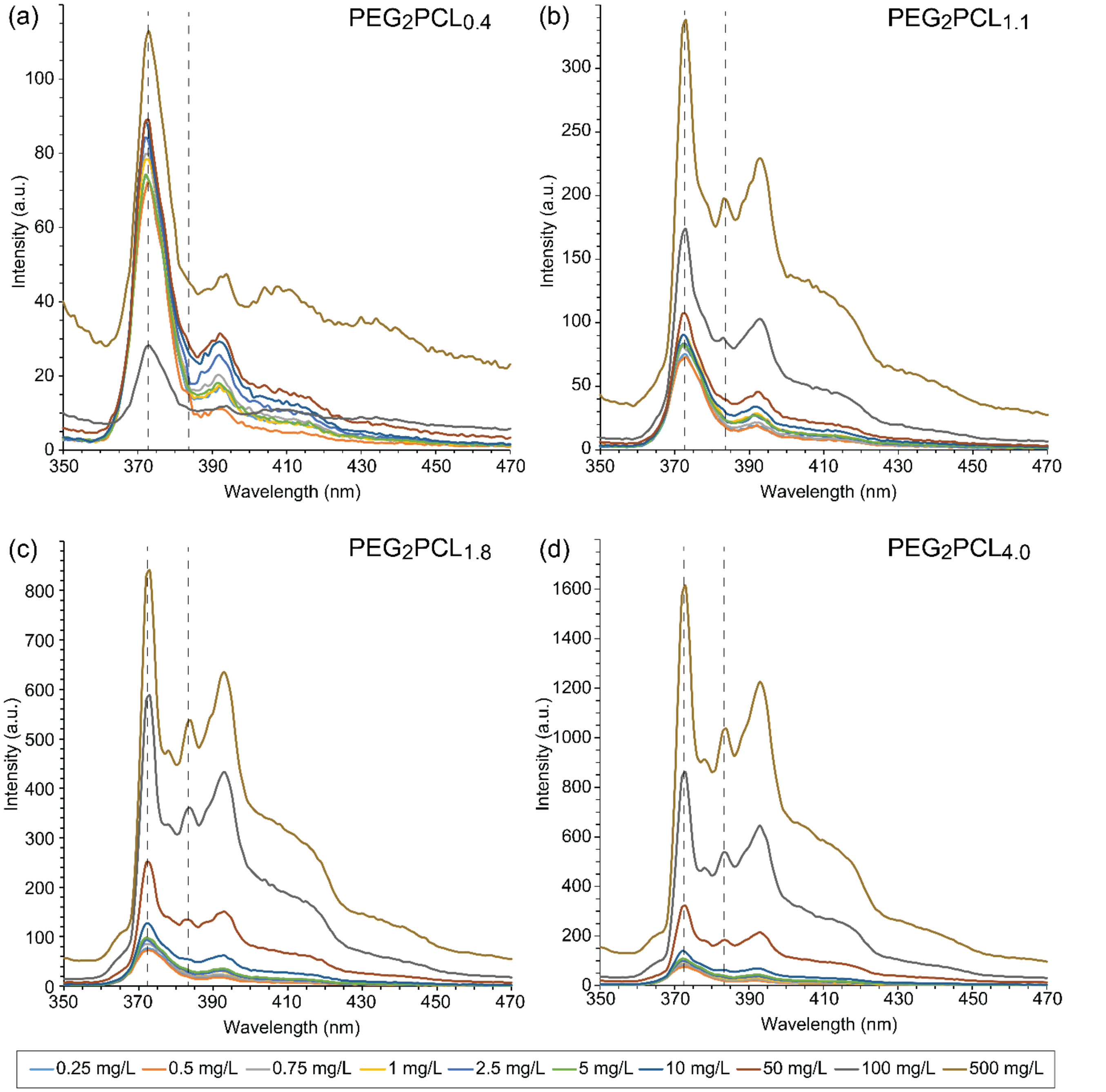

Figure S1. Pyrene (6 x 10^{-7} M) fluorescent emission spectra (λ_{ex} = 334 nm) for (a) $PEG_2PCL_{0.4}$, (b) $PEG_2PCL_{1.1}$, (c) $PEG_2PCL_{1.8}$ and (d) $PEG_2PCL_{4.0}$ copolymers at various copolymer concentrations.

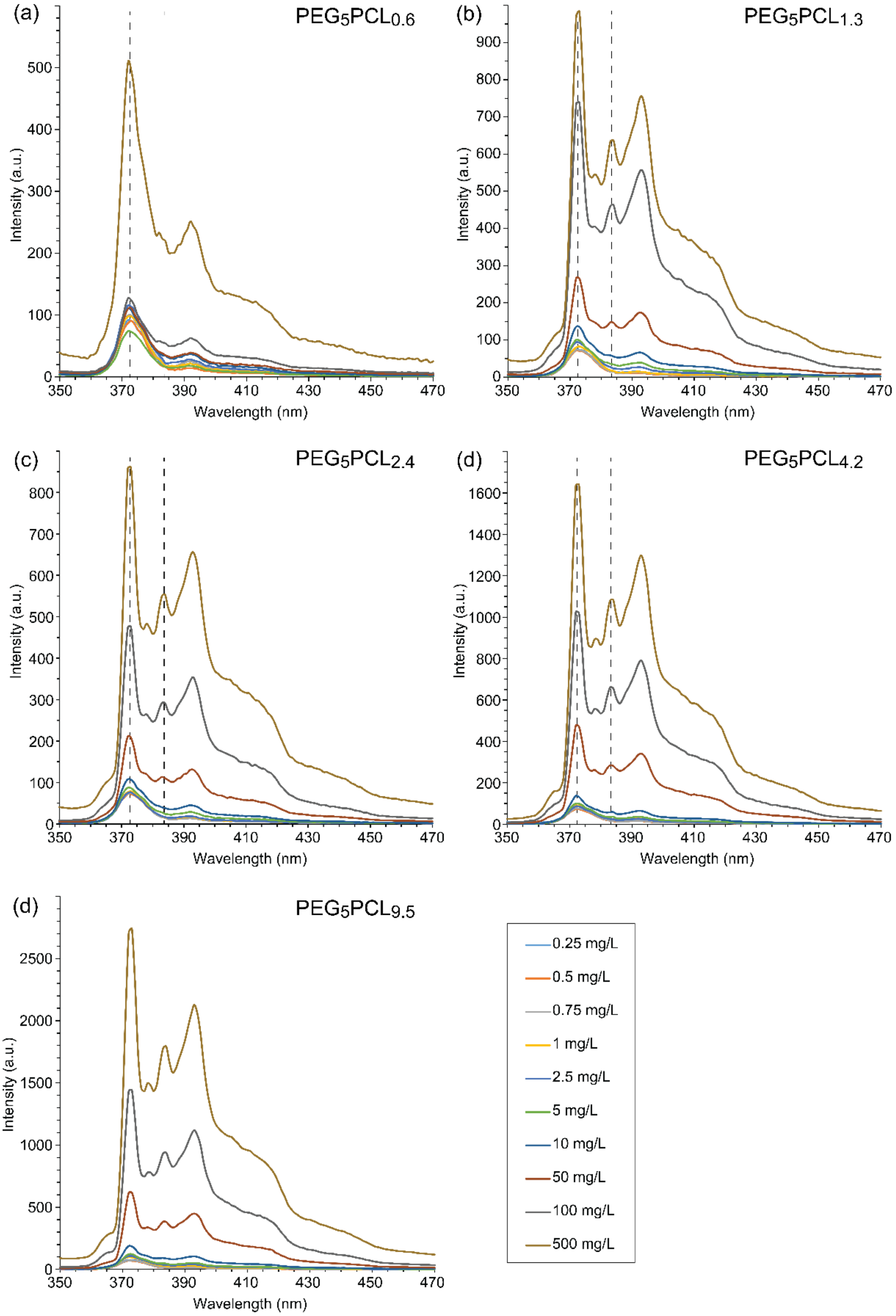

Figure S2. Pyrene (6 x 10^{-7} M) fluorescent emission spectra (λ_{ex} = 334 nm) for (a) $PEG_5PCL_{0.6}$, (b) $PEG_5PCL_{1.3}$, (c) $PEG_5PCL_{2.4}$, (d) $PEG_5PCL_{4.2}$ and (e) $PEG_5PCL_{9.5}$ copolymers at various copolymer concentrations.

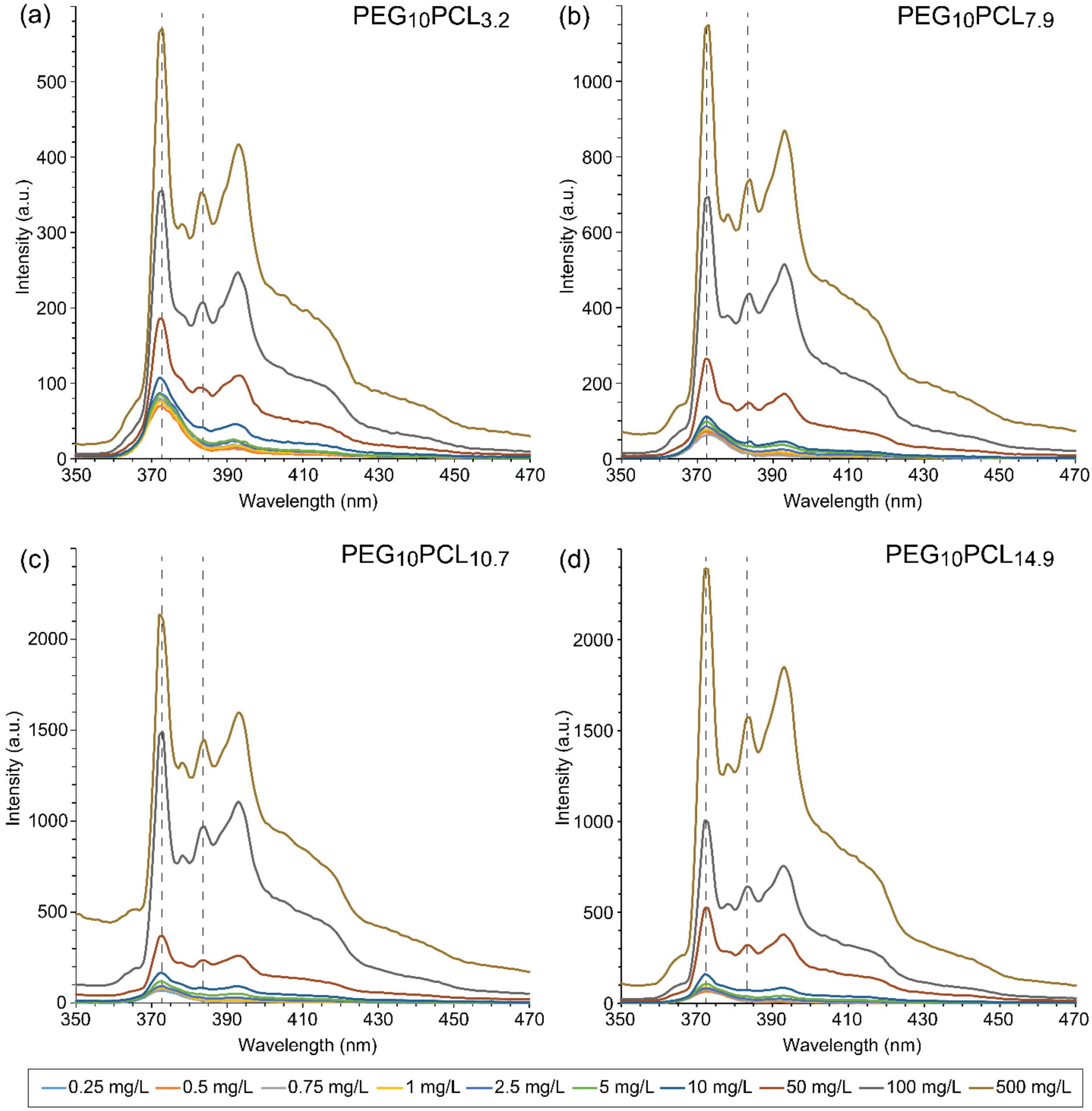

Figure S3. Pyrene (6 x 10^{-7} M) fluorescent emission spectra (λ_{ex} = 334 nm) for (a) $PEG_{10}PCL_{3.2}$, (b) $PEG_{10}PCL_{7.9}$, (c) $PEG_{10}PCL_{10.7}$ and (d) $PEG_{10}PCL_{14.9}$ copolymers at various copolymer concentrations.

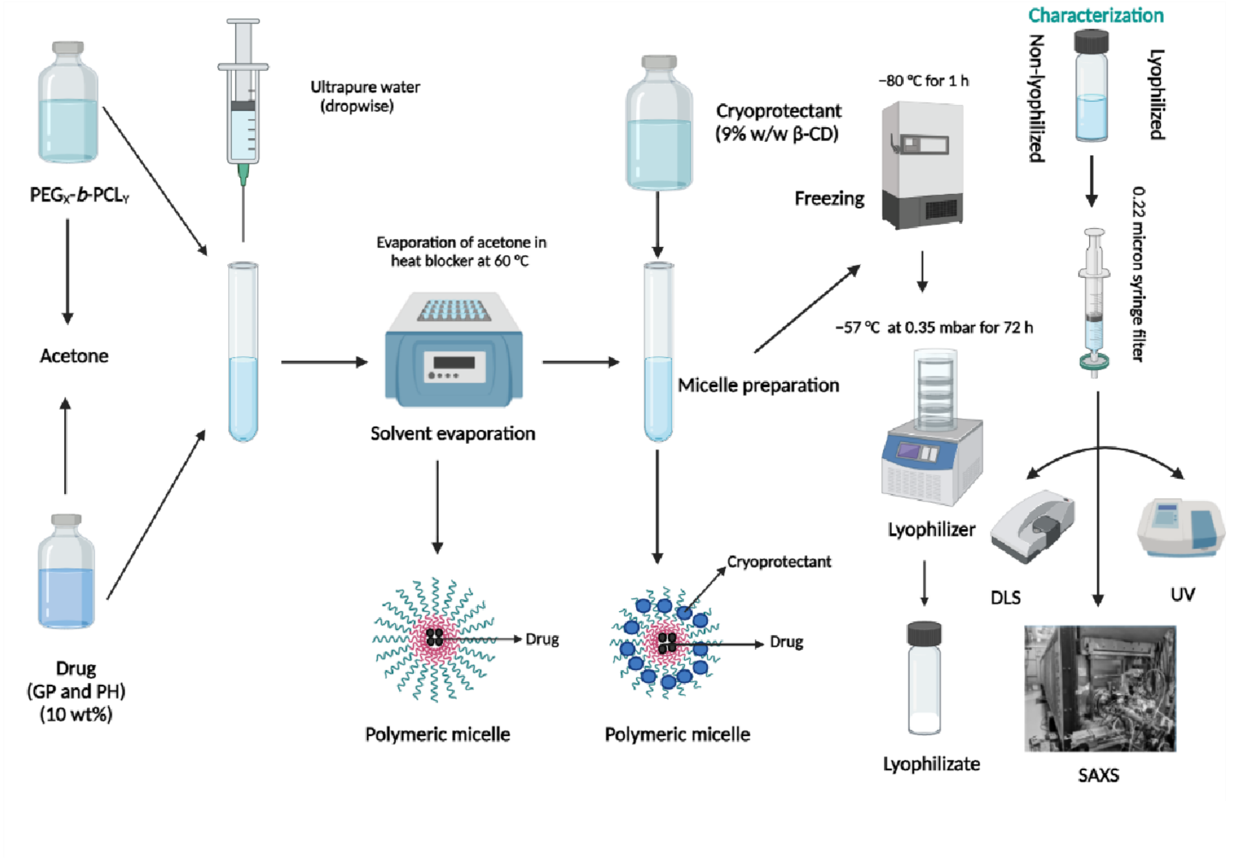

Figure S4. Illustration of preparation, lyophilization and characterisation of PEGX-*b*-PCLY micelles.

Table S2. Polydispersity index (PDI) of micelles before and after lyophilization/reconstitution in the absence and presence of β-CD (-/+CD) as cryoprotectant, as determined by dynamic light scattering DLS; values are expressed as mean ± standard deviation (n=3).

Copolymer	Blank	Gossypol (GP)	Phloretin (PH)	Before freeze-drying (-CD) (PDI ± SD)	Before freeze-drying (+CD) (PDI ± SD)	After freeze-drying (-CD) (PDI ± SD)	After freeze-drying (+CD) (PDI ± SD)
$PEG_2PCL_{0.4}$	√	-	-	0.149 ± 0.010	0.156 ± 0.009	0.196 ± 0.010	0.185 ± 0.013
$PEG_2PCL_{1.1}$	√	-	-	0.189 ± 0.011	0.245 ± 0.014	0.145 ± 0.048	0.149 ± 0.008
$PEG_2PCL_{1.1}$	-	√	-	0.140 ± 0.004	0.179 ± 0.052	0.162 ± 0.025	0.145 ± 0.005
$PEG_2PCL_{1.1}$	-	-	√	0.135 ± 0.023	0.158 ± 0.003	0.157 ± 0.009	0.126 ± 0.002
$PEG_2PCL_{1.8}$	√	-	-	0.225 ± 0.006	0.252 ± 0.007	0.141 ± 0.001	0.228 ± 0.002
$PEG_2PCL_{1.8}$	-	√	-	0.160 ± 0.025	0.187 ± 0.034	0.136 ± 0.008	0.132 ± 0.005
$PEG_2PCL_{1.8}$	-	-	√	0.147 ± 0.011	0.178 ± 0.030	0.158 ± 0.008	0.184 ± 0.005
$PEG_2PCL_{4.0}$	√	-	-	0.245 ± 0.030	0.195 ± 0.003	0.210 ± 0.011	0.186 ± 0.039
$PEG_5PCL_{0.6}$	√	-	-	0.262 ± 0.007	0.242 ± 0.003	0.210 ± 0.016	0.177 ± 0.050
$PEG_5PCL_{1.3}$	√	-	-	0.241 ± 0.008	0.245 ± 0.038	0.185 ± 0.016	0.188 ± 0.006
$PEG_5PCL_{1.3}$	-	√	-	0.126 ± 0.010	0.185 ± 0.016	0.148 ± 0.006	0.147 ± 0.007
$PEG_5PCL_{1.3}$	-	-	√	0.205 ± 0.003	0.257 ± 0.050	0.205 ± 0.003	0.246 ± 0.056
$PEG_5PCL_{2.4}$	√	-	-	0.192 ± 0.008	0.145 ± 0.030	0.176 ± 0.035	0.163 ± 0.011
$PEG_5PCL_{2.4}$	-	√	-	0.220 ± 0.060	0.201 ± 0.004	0.236 ± 00.17	0.158 ± 0.008
$PEG_5PCL_{2.4}$	-	-	√	0.197 ± 0.002	0.139 ± 0.005	0.131 ± 0.002	0.159 ± 0.014
$PEG_5PCL_{4.2}$	√	-	-	0.123 ± 0.006	0.132 ± 0.046	0.176 ± 0.006	0.138 ± 0.005
$PEG_5PCL_{4.2}$	-	√	-	0.192 ± 0.004	0.175 ± 0.003	0.156 ± 0.019	0.158 ± 0.016
$PEG_5PCL_{4.2}$	-	-	√	0.168 ± 0.028	0.085 ± 0.009	0.140 ± 0.040	0.137 ± 0.034
$PEG_5PCL_{9.5}$	√	-	-	0.138 ± 0.004	0.145 ± 0.011	0.128 ± 0.011	0.116 ± 0.021
$PEG_5PCL_{9.5}$	-	√	-	0.193 ± 0.002	0.197 ± 0.005	0.153 ± 0.011	0.156 ± 0.016

$PEG_5PCL_{9.5}$	-	-	√	0.144 ± 0.013	0.127 ± 0.011	0.118 ± 0.019	0.168 ± 0.030
$PEG_{10}PCL_{3.2}$	√	-	-	0.173 ± 0.004	0.186 ± 0.006	0.133 ± 0.002	0.186 ± 0.015
$PEG_{10}PCL_{7.9}$	√	-	-	0.170 ± 0.009	0.176 ± 0.003	0.195 ± 0.020	0.172 ± 0.009
$PEG_{10}PCL_{7.9}$	-	√	-	0.183 ± 0.013	0.172 ± 0.007	0.243 ± 0.011	0.232 ± 0.009
$PEG_{10}PCL_{7.9}$	-	-	√	0.149 ± 0.014	0.164 ± 0.006	0.182 ± 0.006	0.216 ± 0.024
$PEG_{10}PCL_{10.7}$	√	-	-	0.146 ± 0.017	0.149 ± 0.007	0.119 ±0.006	0.125 ± 0.002
$PEG_{10}PCL_{10.7}$	-	√	-	0.138 ± 0.006	0.153 ± 0.008	0.186 ± 0.006	0.187 ± 0.013
$PEG_{10}PCL_{10.7}$	-	-	√	0.116 ± 0.007	0.225 ± 0.042	0.230 ± 0.005	0.191 ± 0.017
$PEG_{10}PCL_{14.9}$	√	-	-	0.160 ± 0.008	0.160 ± 0.011	0.128 ±0.013	0.187 ± 0.025

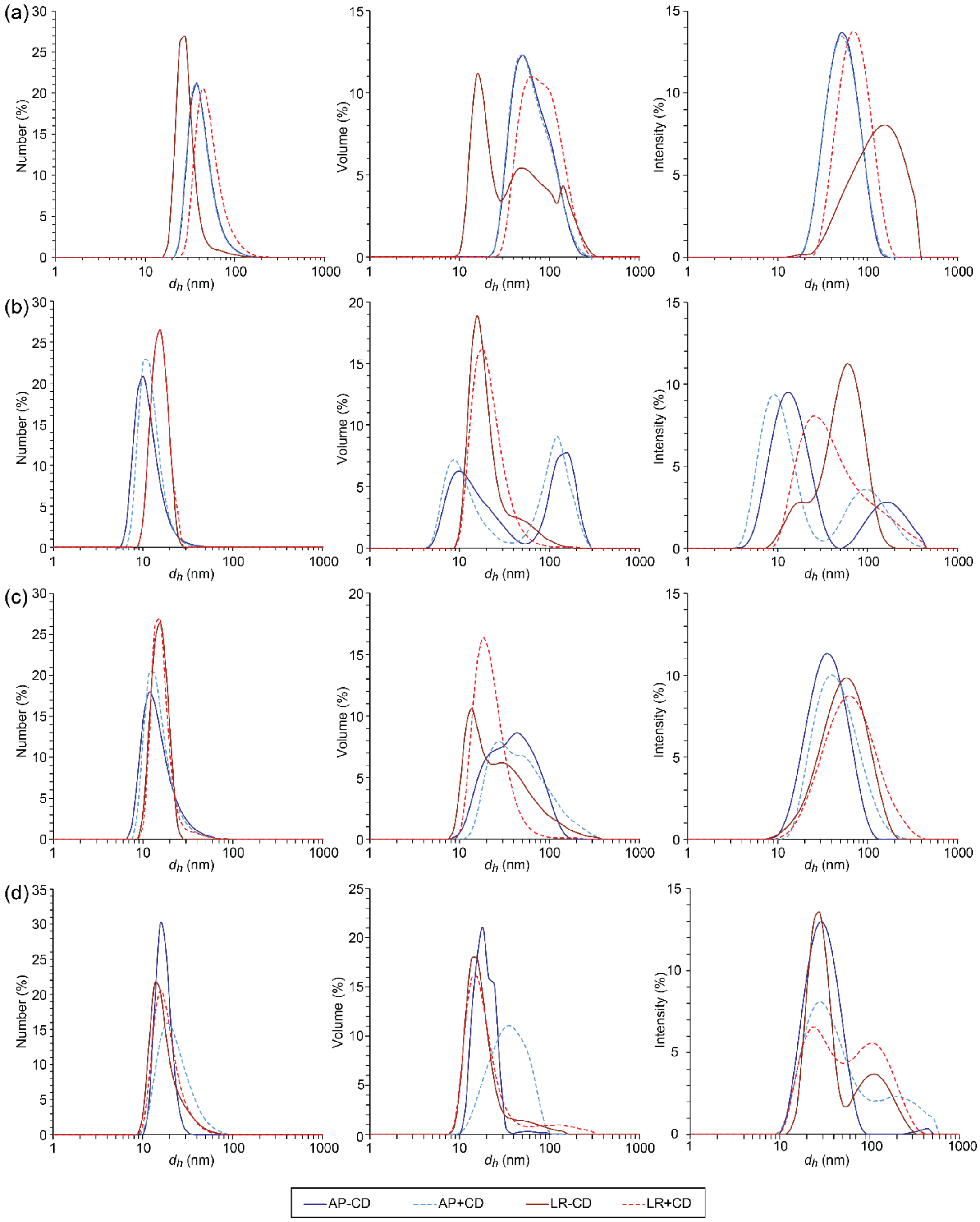

Figure S5. Number, volume and intensity particle size distributions for (a) $PEG_2PCL_{0.4}$, (b) $PEG_2PCL_{1.1}$, (c) $PEG_2PCL_{1.8}$ and (d) $PEG_2PCL_{4.0}$ micelles as prepared (AP) and after lyophilization/reconstitution (LR) in the absence and presence of β-CD (-/+CD).

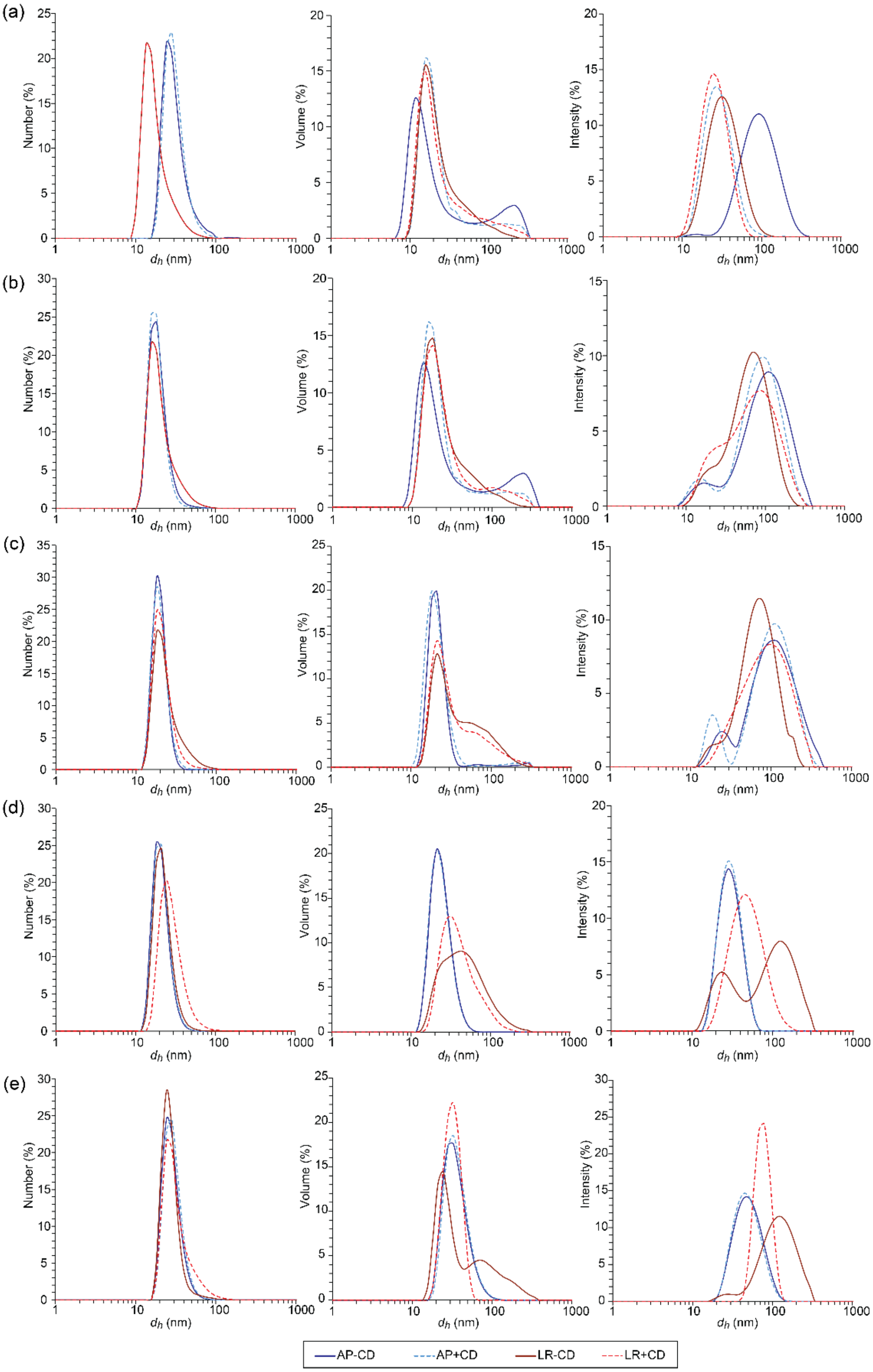

Figure S6. Number, volume and intensity particle size distributions for (a) PEG$_5$PCL$_{0.6}$, (b) PEG$_5$PCL$_{1.3}$, (c) PEG$_5$PCL$_{2.4}$, (d) PEG$_5$PCL$_{4.2}$ and (e) PEG$_5$PCL$_{9.5}$ micelles as prepared (AP) and after lyophilization/reconstitution (LR) in the absence and presence of β-CD (-/+CD).

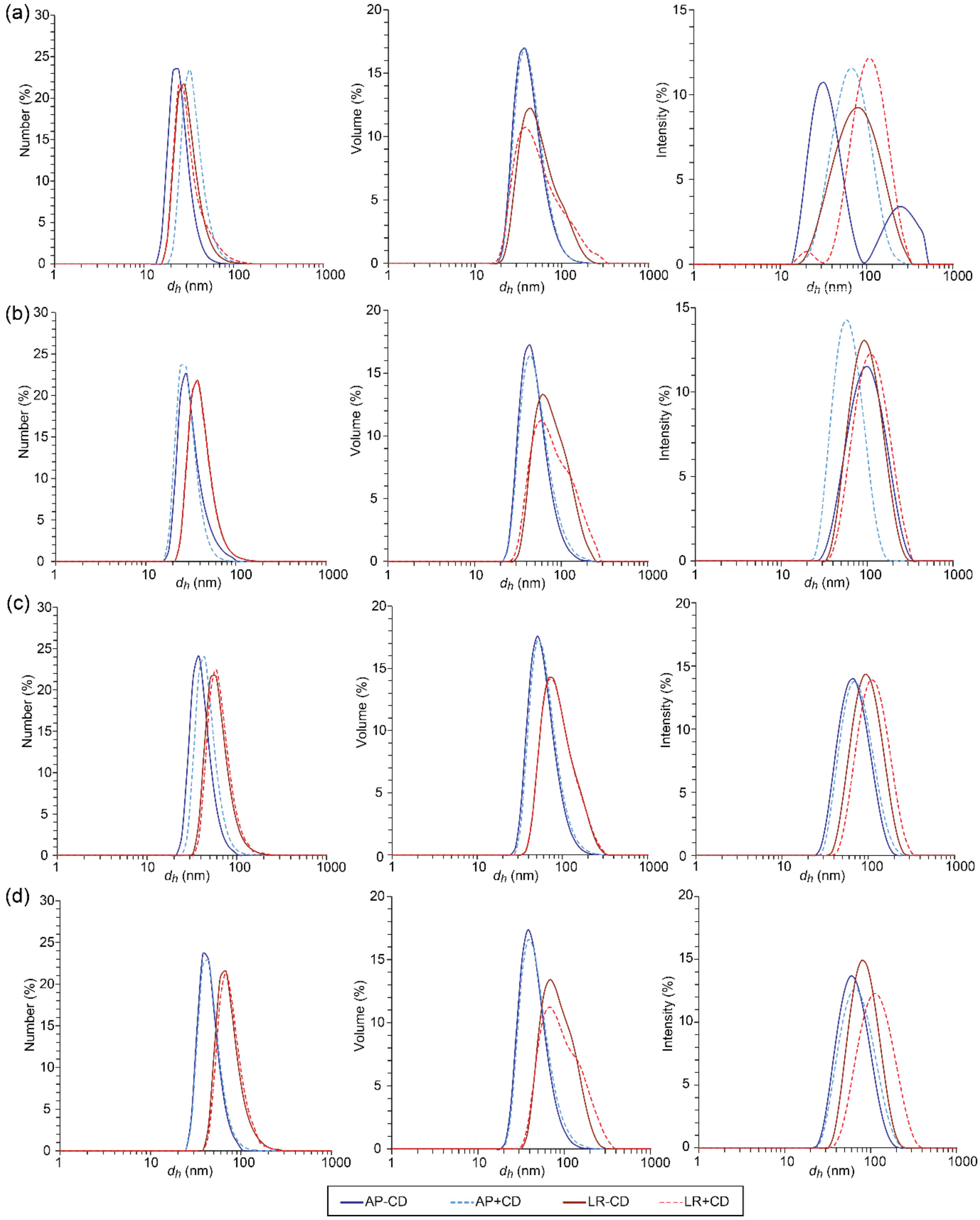

Figure S7. Number, volume and intensity particle size distributions for (a) $PEG_{10}PCL_{3.2}$, (b) $PEG_{10}PCL_{7.9}$, (c) $PEG_{10}PCL_{10.7}$ and (d) $PEG_{10}PCL_{14.9}$ micelles as prepared (AP) and after lyophilization/reconstitution (LR) in the absence and presence of β-CD (-/+CD).

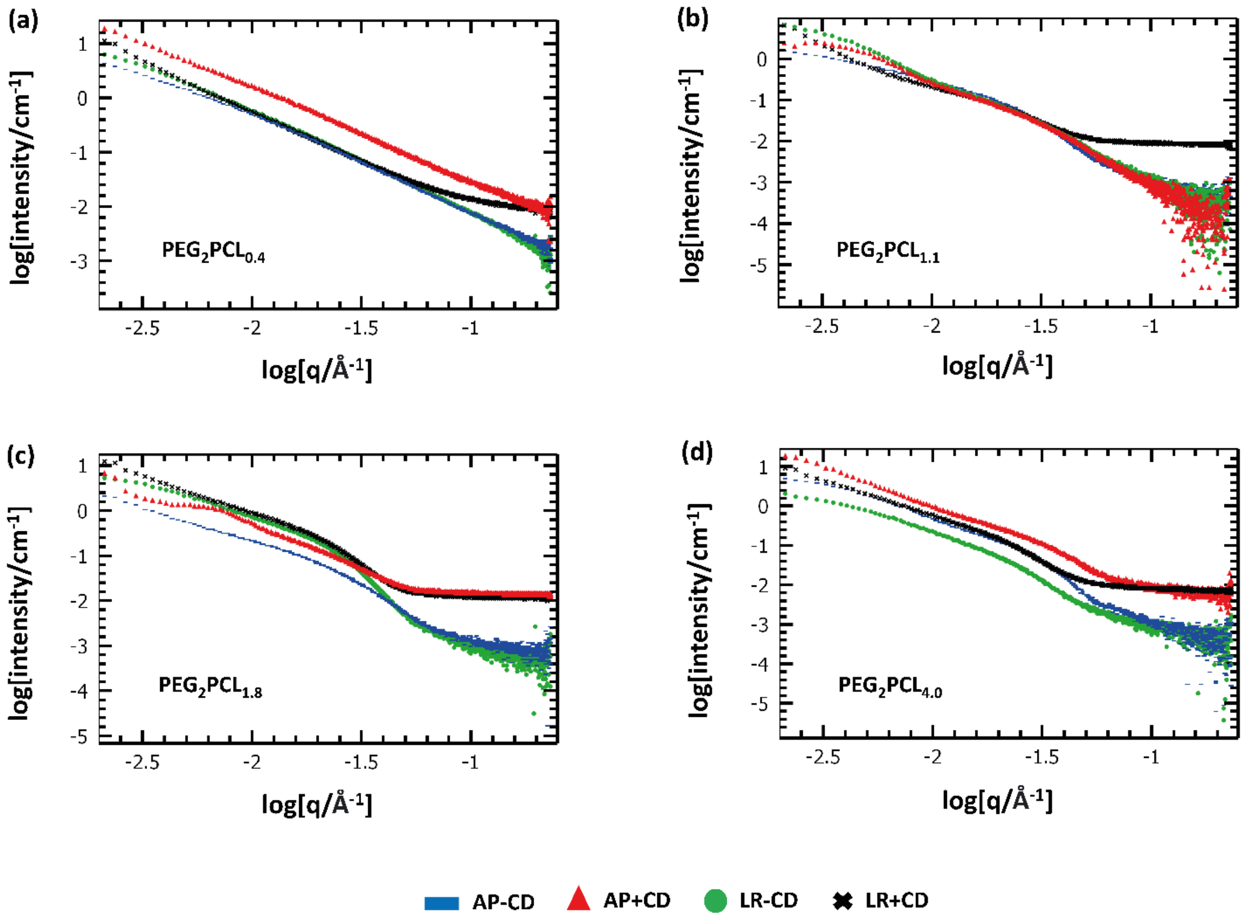

Figure S8. SAXS profiles for (a) $PEG_2PCL_{0.4}$, (b) $PEG_2PCL_{1.1}$, (c) $PEG_2PCL_{1.8}$ and (d) $PEG_2PCL_{4.0}$ micelles/aggregates as prepared (AP) and after lyophilization/reconstitution (LR) in the absence and presence of β-CD (-/+CD).

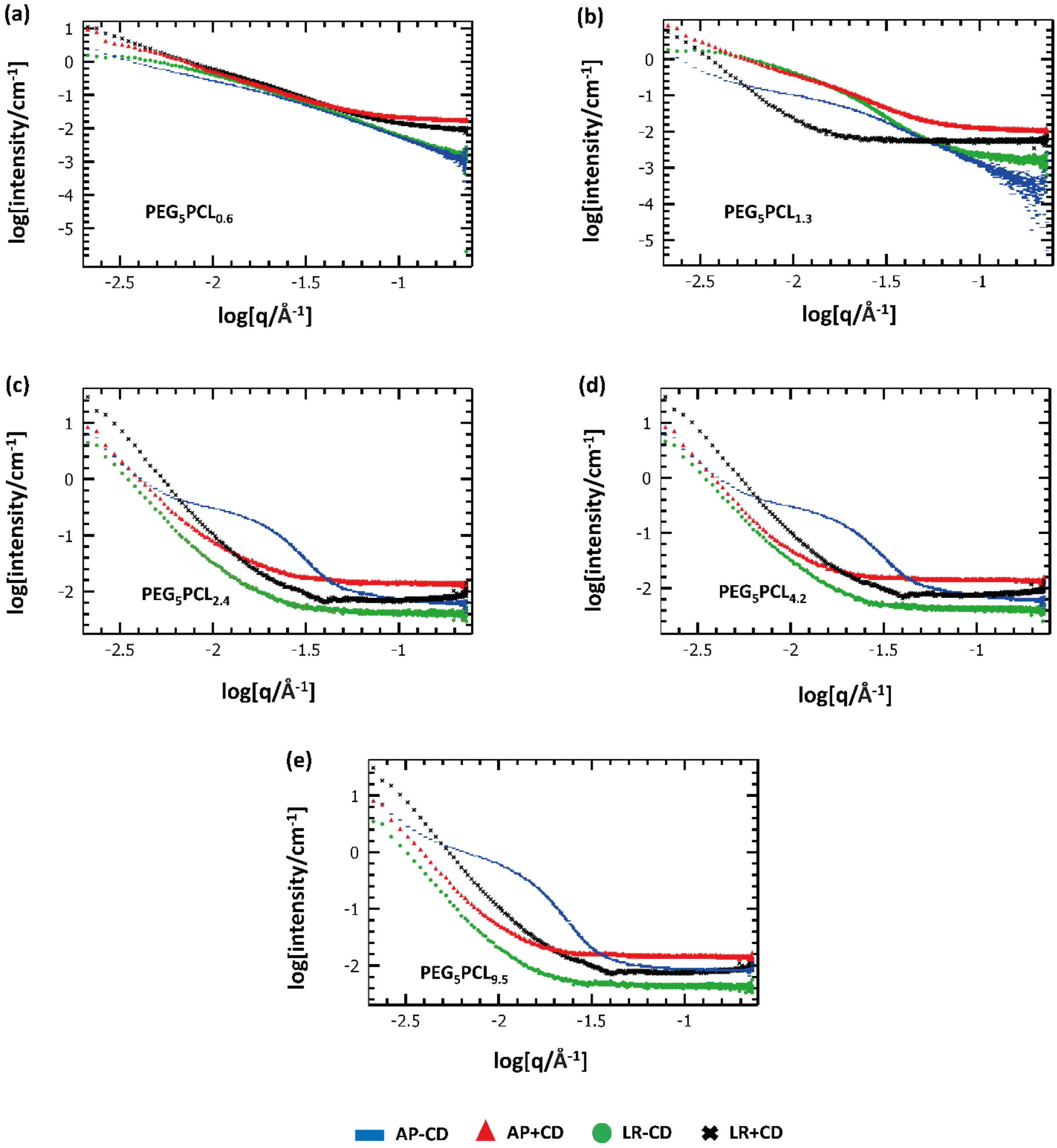

Figure S9. SAXS profiles for (a) PEG$_5$PCL$_{0.6}$, (b) PEG$_5$PCL$_{1.3}$, (c) PEG$_5$PCL$_{2.4}$, (d) PEG$_5$PCL$_{4.2}$ and (e) PEG$_5$PCL$_{9.5}$ micelles/aggregates as prepared (AP) and after lyophilization/reconstitution (LR) in the absence and presence of β-CD (-/+CD). Background multiplication factor (BMF) for PEG$_5$PCL$_{1.3}$ LR+CD, PEG$_5$PCL$_{2.4}$ LR+CD, PEG$_5$PCL$_{4.2}$ LR+CD and PEG$_5$PCL$_{9.5}$ LR+CD was 0.72, 0.55, 0.55 and 0.53, respectively.

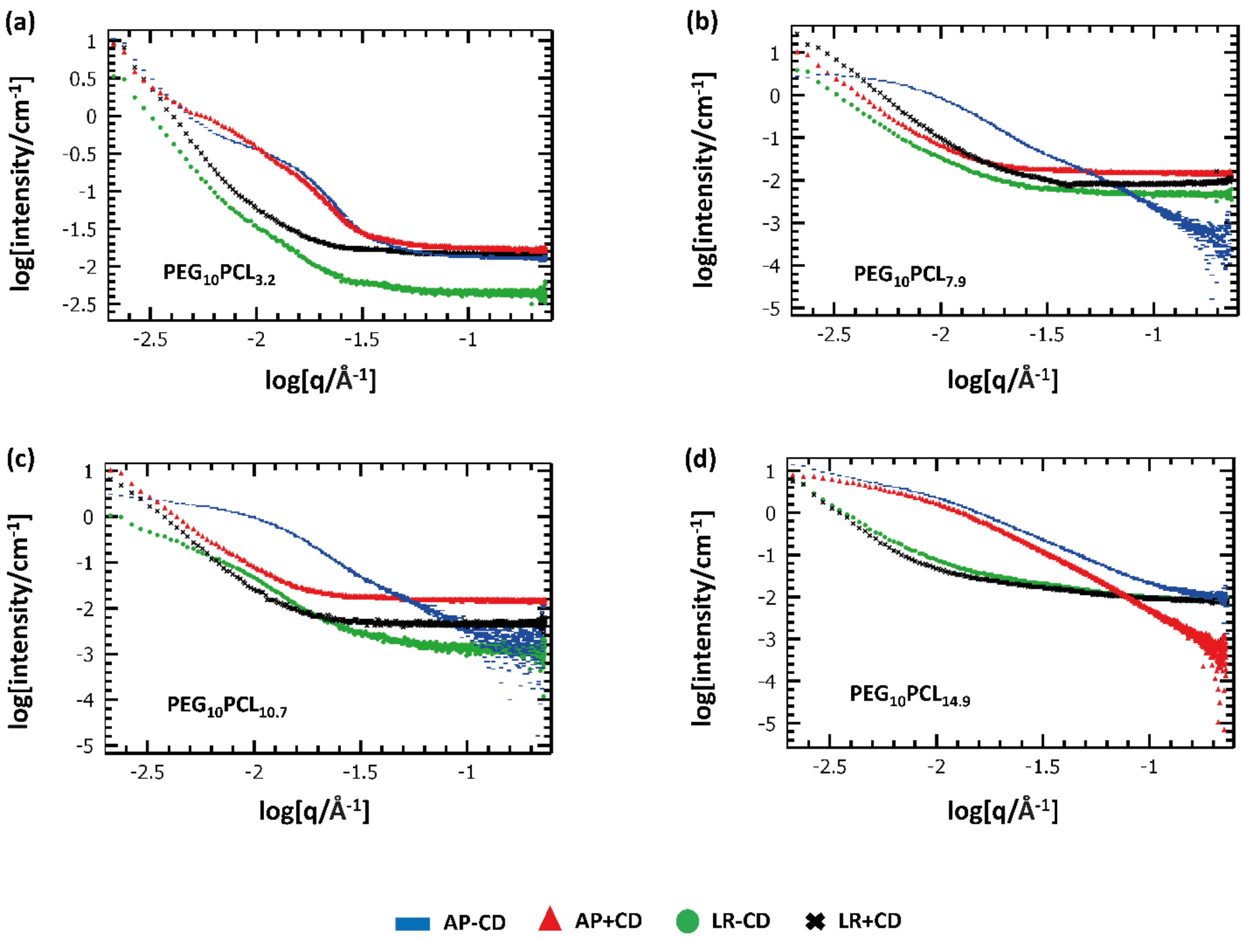

Figure S10. SAXS profiles for (a) $PEG_{10}PCL_{3.2}$, (b) $PEG_{10}PCL_{7.9}$, (c) $PEG_{10}PCL_{10.7}$ and (d) $PEG_{10}PCL_{14.9}$ micelles/aggregates as prepared (AP) and after lyophilization/reconstitution (LR) in the absence and presence of β-CD (-/+CD) (BMF for $PEG_{10}PCL_{3.2}$ LR+CD, $PEG_{10}PCL_{7.9}$ LR+CD and $PEG_{10}PCL_{10.7}$ LR+CD was 0.45, 0.55 and 0.53, respectively).

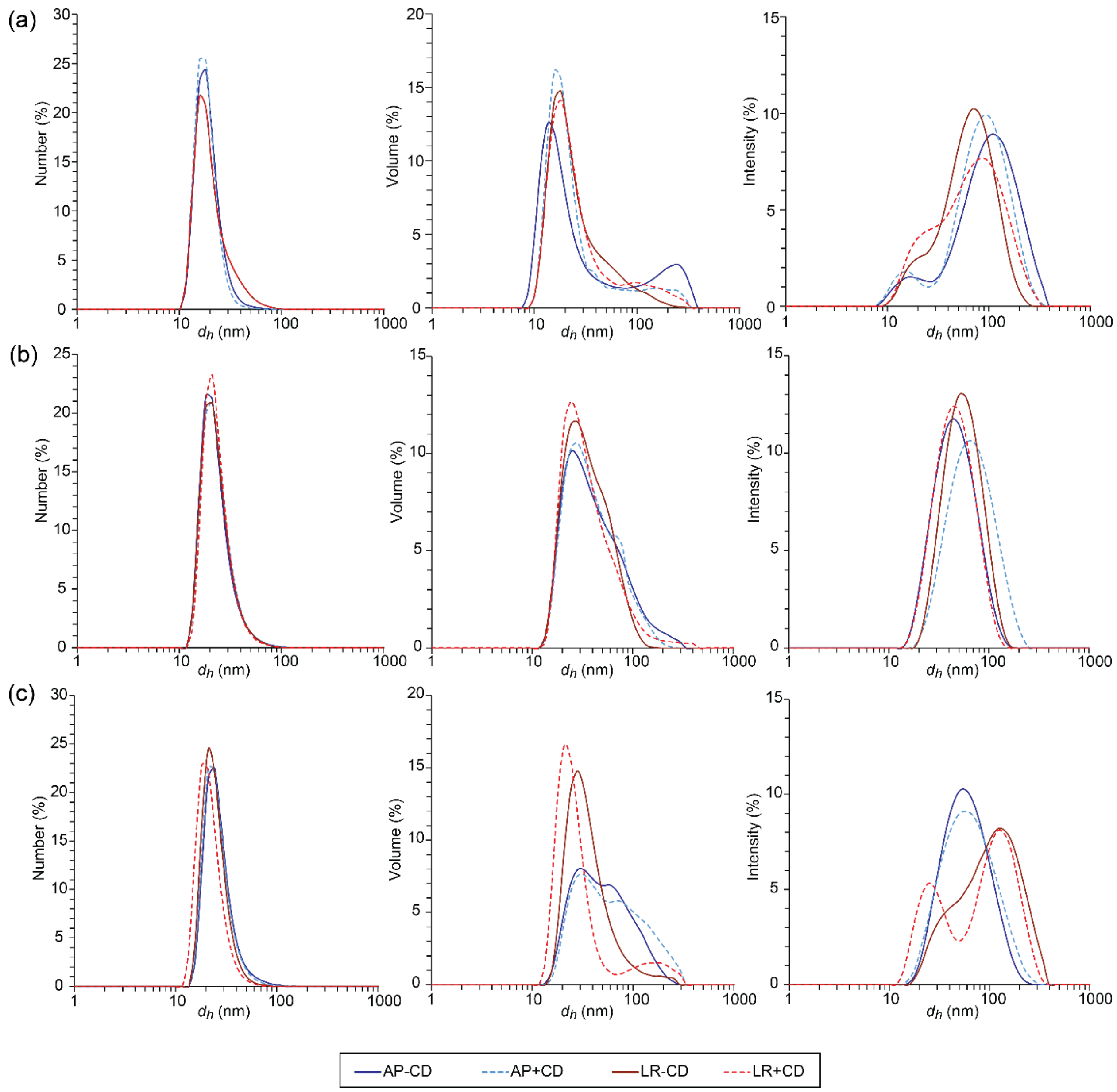

Figure S11. Number, volume and intensity particle size distributions for $PEG_5PCL_{1.3}$ (a) blank micelles and (b) gossypol (GP) and (c) phloretin (PH) loaded micelles as prepared (AP) and after lyophilization/reconstitution (LR) in the absence and presence of β-CD (-/+CD).

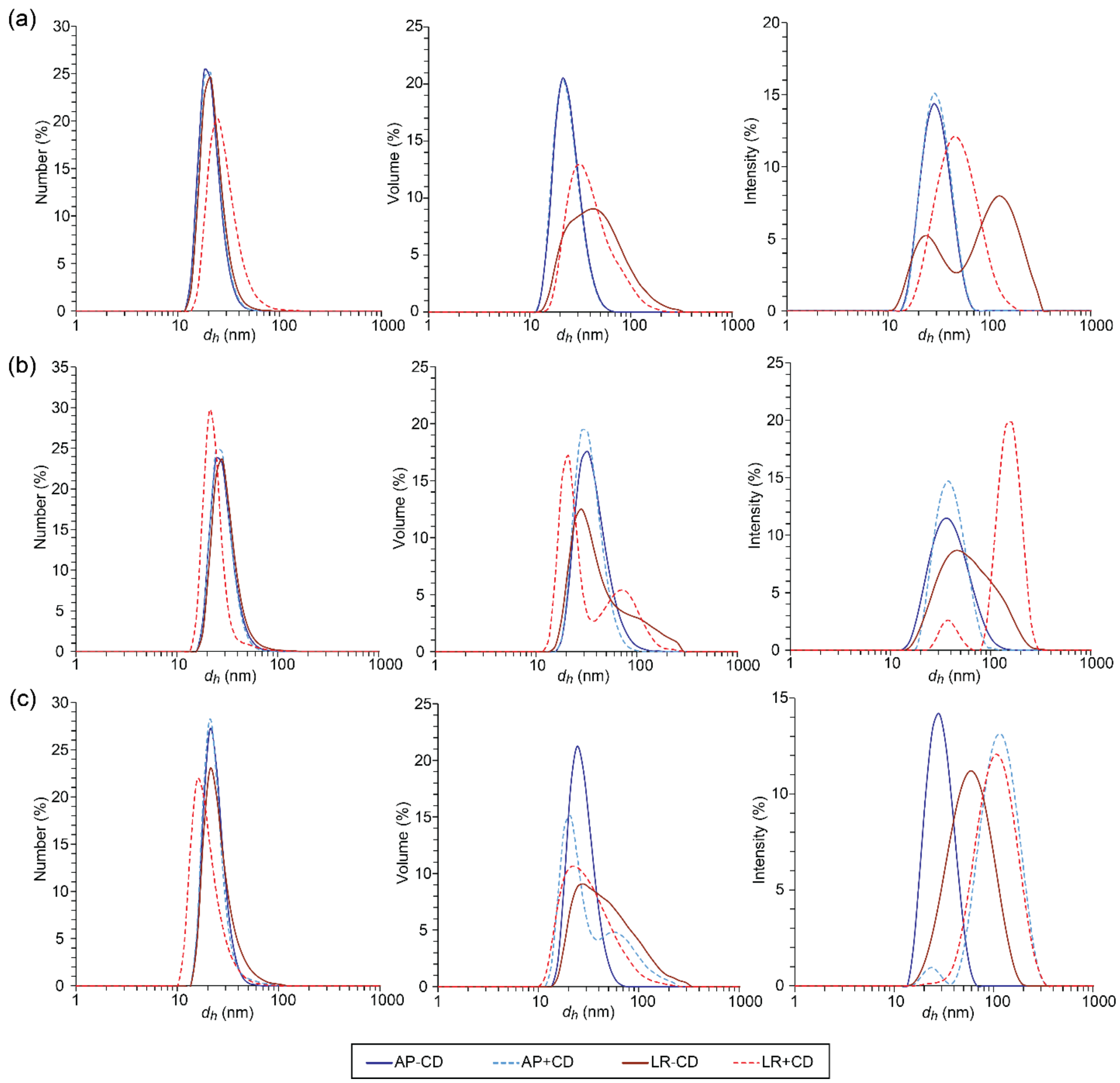

Figure S12. Number, volume and intensity particle size distributions for $PEG_5PCL_{2.4}$ (a) blank micelles and (b) gossypol (GP) and (c) phloretin (PH) loaded micelles as prepared (AP) and after lyophilization/reconstitution (LR) in the absence and presence of β-CD (-/+CD).

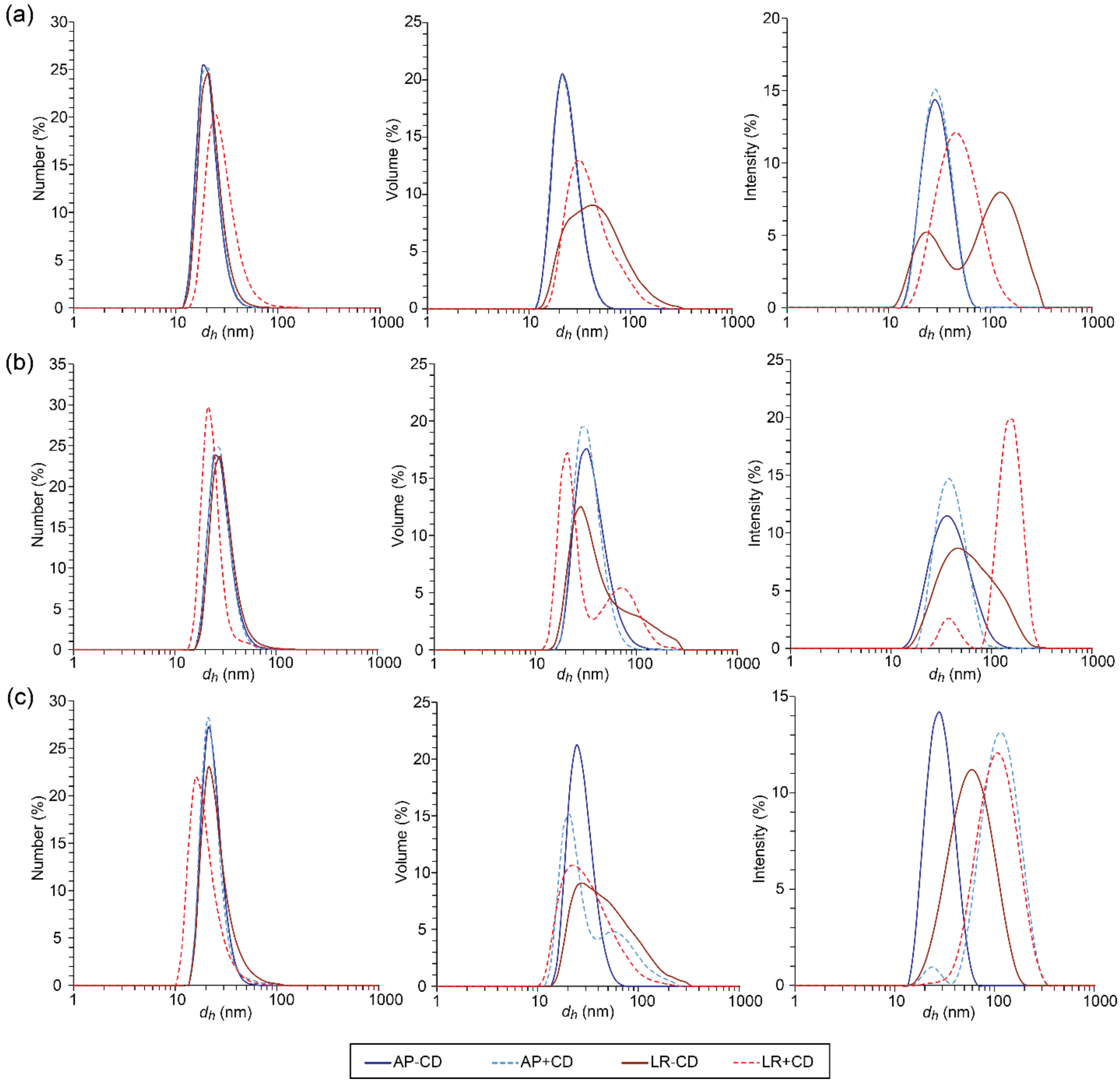

Figure S13. Number, volume and intensity particle size distributions for $PEG_5PCL_{4.2}$ (a) blank micelles and (b) gossypol (GP) and (c) phloretin (PH) loaded micelles as prepared (AP) and after lyophilization/reconstitution (LR) in the absence and presence of β-CD (-/+CD).

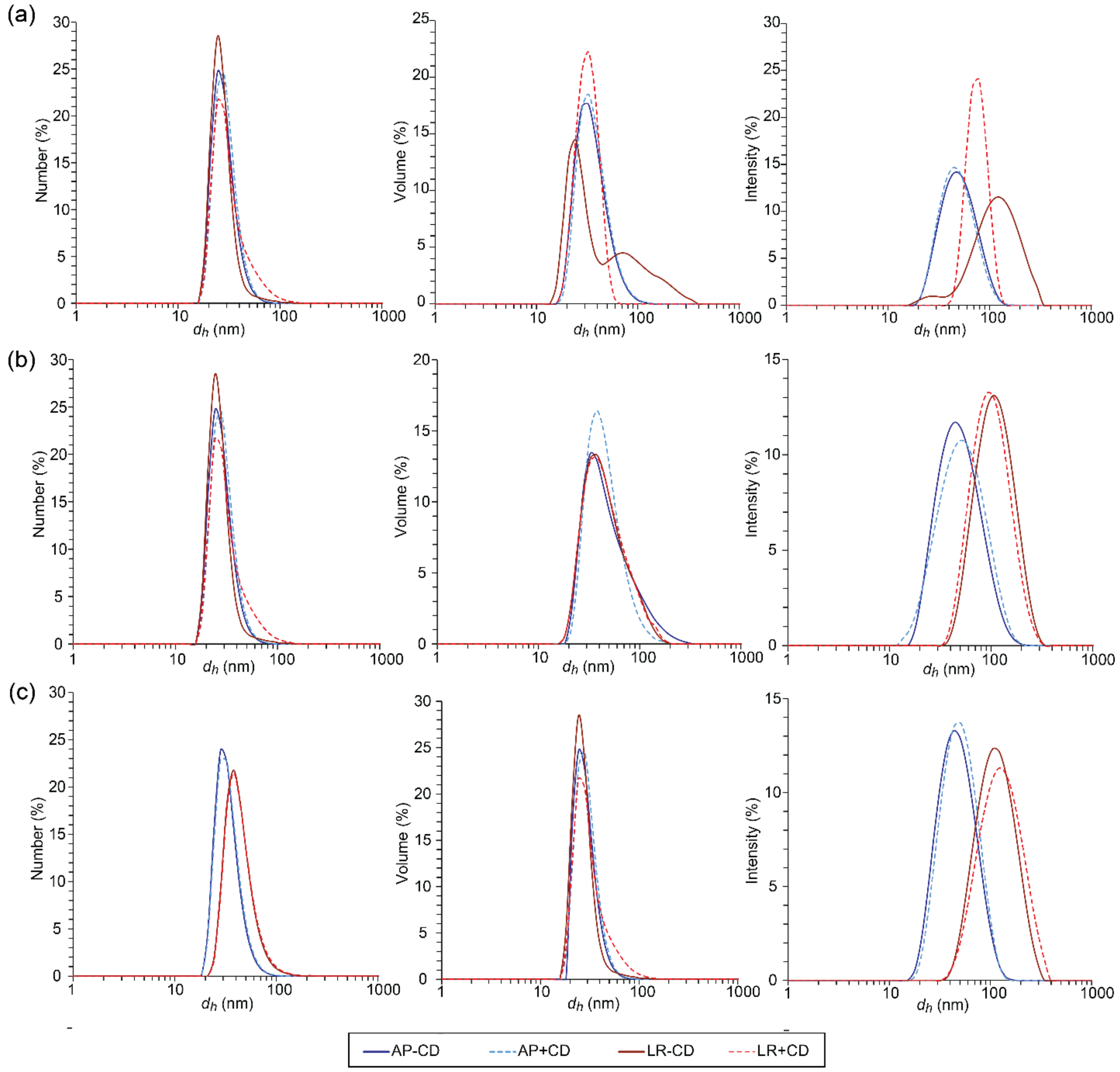

Figure S14. Number, volume and intensity particle size distributions for $PEG_5PCL_{9.5}$ (a) blank micelles and (b) gossypol (GP) and (c) phloretin (PH) loaded micelles as prepared (AP) and after lyophilization/reconstitution (LR) in the absence and presence of β-CD (-/+CD).

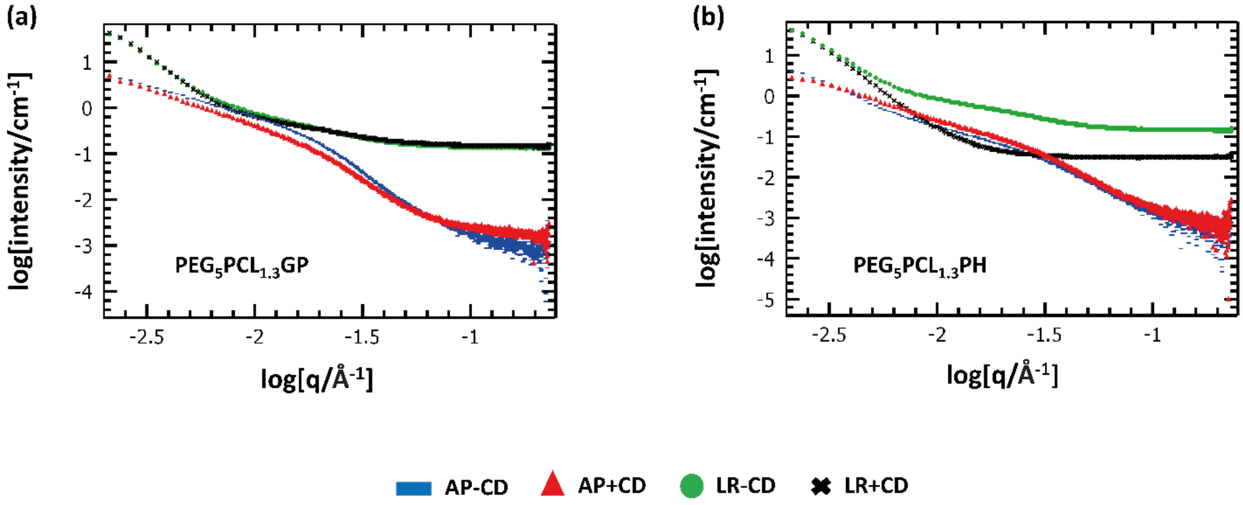

Figure S15. SAXS profiles for (a) gossypol and (b) phloretin loaded $PEG_5PCL_{1.3}$ micelles as prepared (AP) and after lyophilization/reconstitution (LR) in the absence and presence of β-CD (-/+CD).

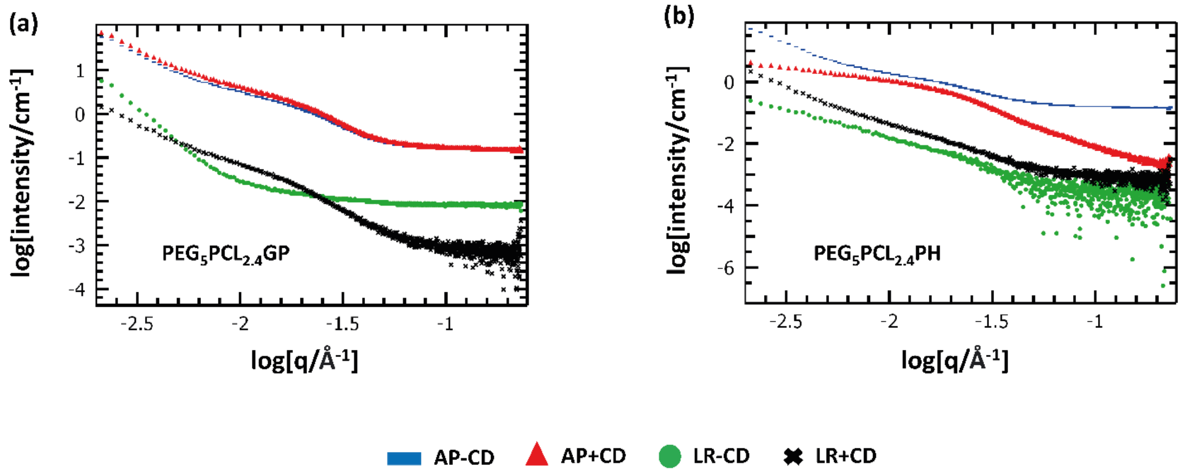

Figure S16. SAXS profiles for (a) gossypol and (b) phloretin loaded $PEG_5PCL_{2.4}$ micelles as prepared (AP) and after lyophilization/reconstitution (LR) in the absence and presence of β-CD (-/+CD).

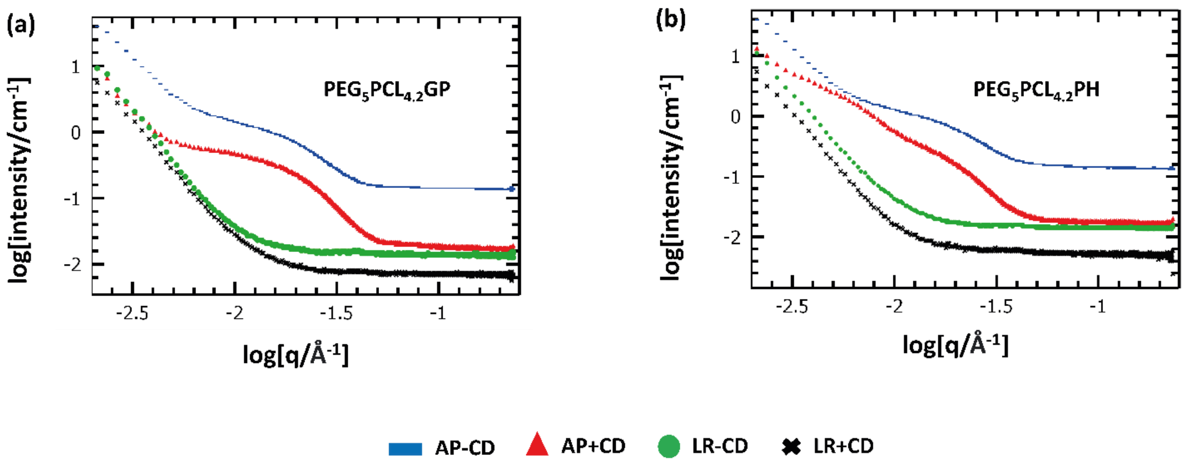

Figure S17. SAXS profiles for (a) gossypol and (b) phloretin loaded $PEG_5PCL_{4.2}$ micelles as prepared (AP) and after lyophilization/reconstitution (LR) in the absence and presence of β-CD (-/+CD) (BMF for $PEG_5PCL_{4.2}$ GP LR+CD and $PEG_5PCL_{4.2}$ PH LR+CD was 0.65 and 0.91, respectively).

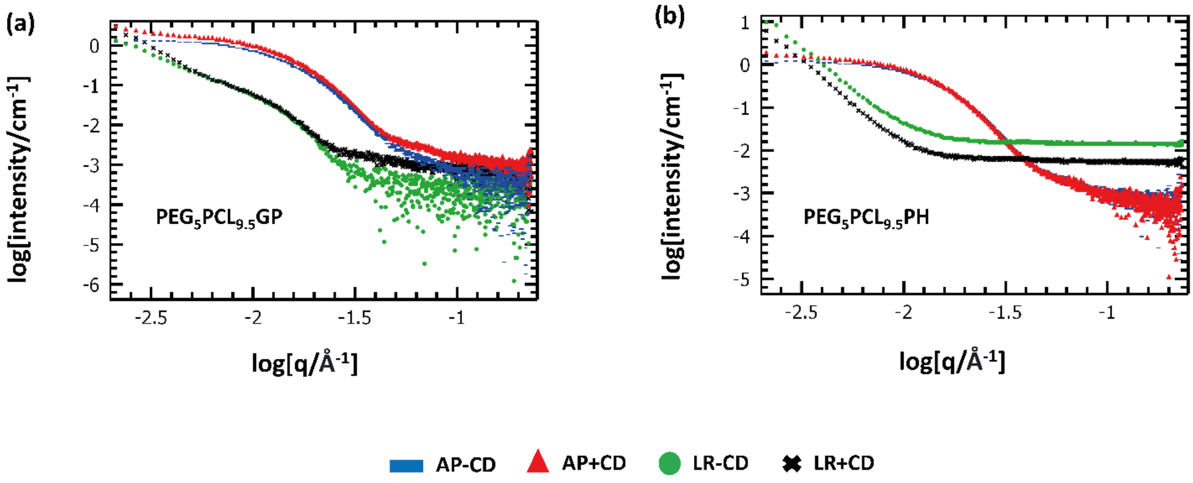

Figure S18. SAXS profiles for (a) gossypol and (b) phloretin loaded $PEG_5PCL_{9.5}$ micelles as prepared (AP) and after lyophilization/reconstitution (LR) in the absence and presence of β-CD (-/+CD) (BMF for $PEG_5PCL_{9.5}$ PH LR+CD was 0.91).

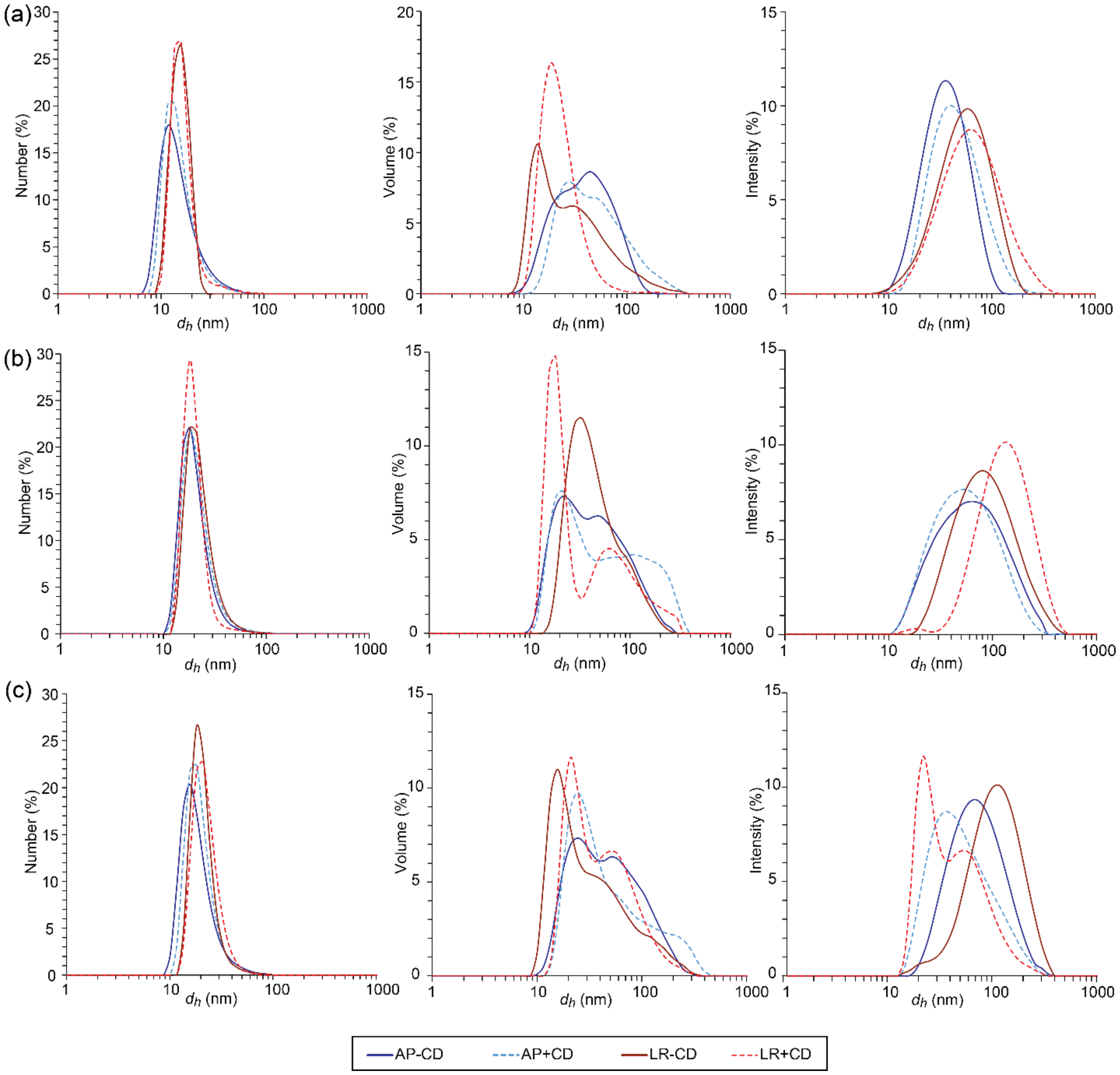

Figure S19. Number, volume and intensity particle size distributions for $PEG_2PCL_{1.8}$ (a) blank micelles and (b) gossypol (GP) and (c) phloretin (PH) loaded micelles as prepared (AP) and after lyophilization/reconstitution (LR) in the absence and presence of β-CD (-/+CD).

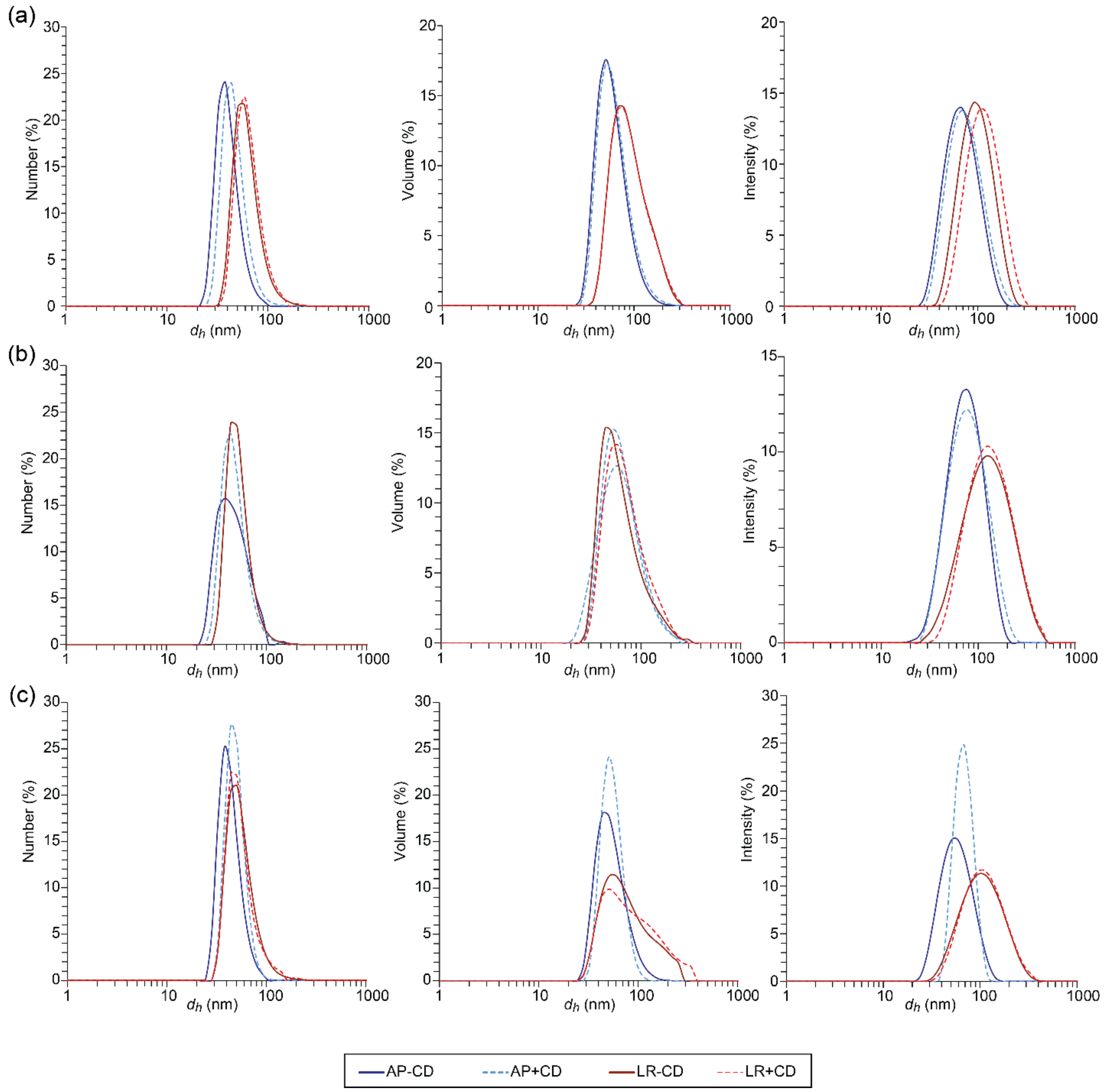

Figure S20. Number, volume and intensity particle size distributions for $PEG_{10}PCL_{10.7}$ (a) blank micelles and (b) gossypol (GP) and (c) phloretin (PH) loaded micelles as prepared (AP) and after lyophilization/reconstitution (LR) in the absence and presence of β-CD (-/+CD).

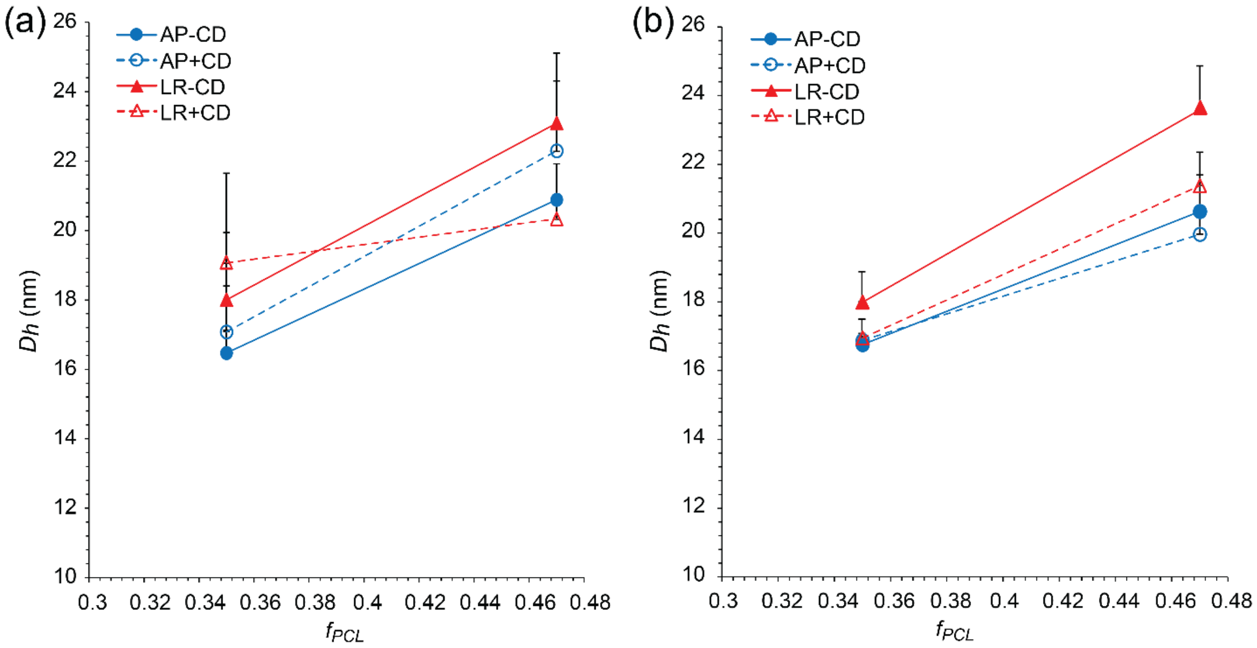

Figure S21. Hydrodynamic diameter (D_h) versus weight fraction of PCL (f_{PCL}) as determined from number particle size distributions (PSDs) for (a) gossypol (GP) and (b) phloretin (PH) loaded $PEG_2PCL_{1.8}$ micelles as prepared (AP) and after lyophilization/reconstitution (LR) and in the absence and presence of β-CD (-/+CD). All values are reported as the mean + std. dev. (n = 3).

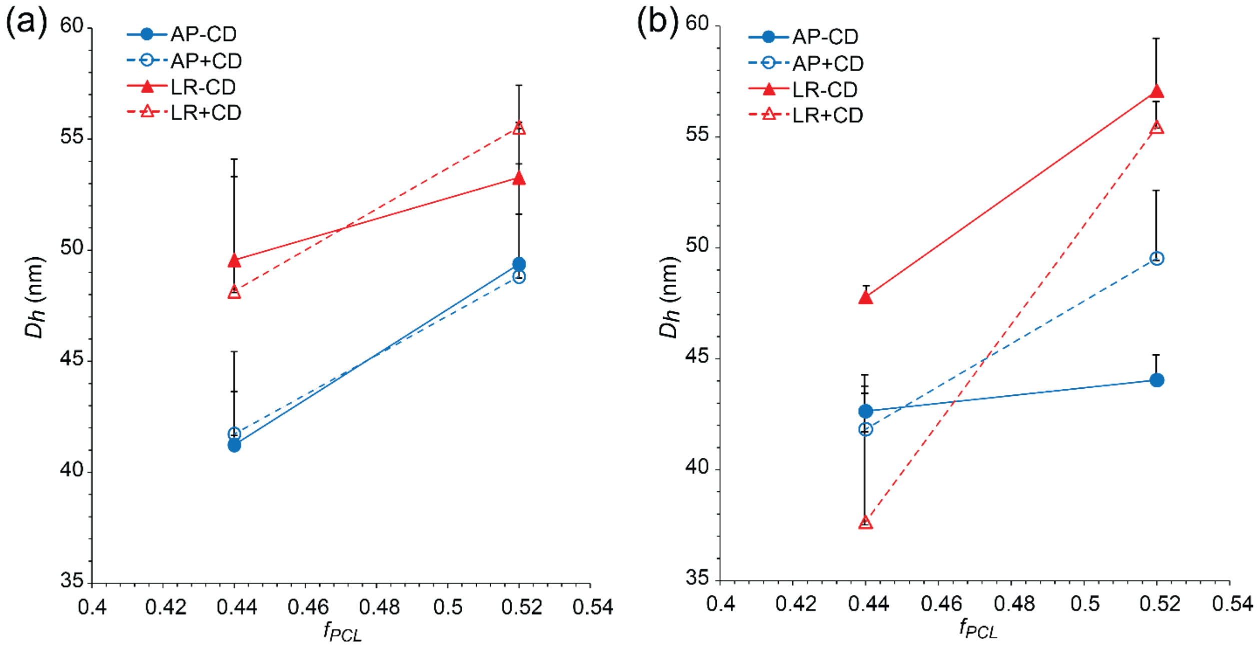

Figure S22. D_h versus f_{PCL} as determined from number PSDs for (a) gossypol (GP) and (b) phloretin (PH) loaded $PEG_{10}PCL_{10.7}$ micelles as prepared (AP) and after lyophilization/reconstitution (LR) and in the absence and presence of β-CD (-/+CD). All values are reported as the mean + std. dev. (n = 3).

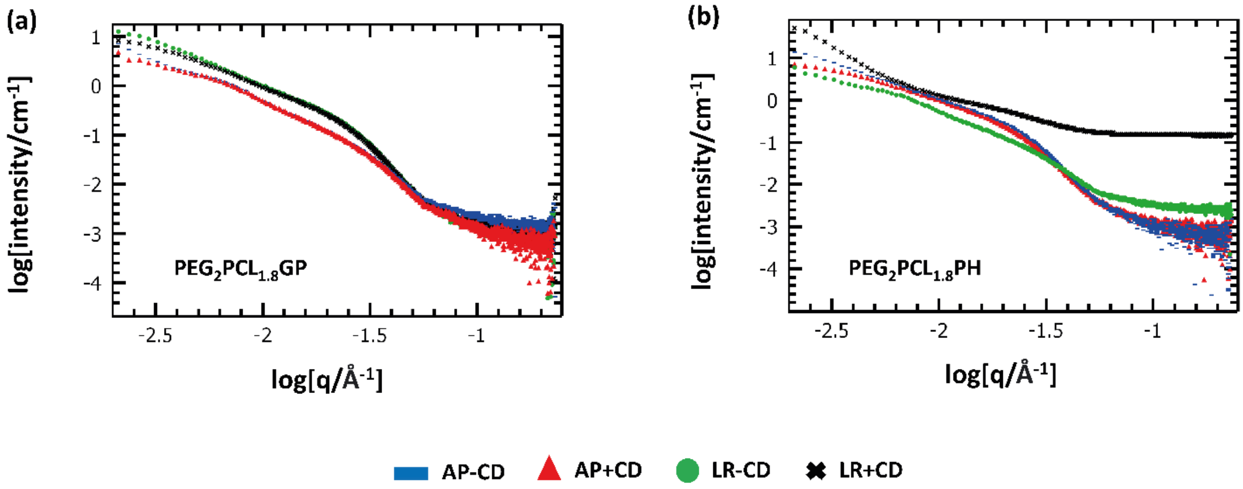

Figure S23. SAXS profiles for (a) gossypol and (b) phloretin loaded $PEG_2PCL_{1.8}$ micelles as prepared (AP) and after lyophilization/reconstitution (LR) in the absence and presence of β-CD (-/+CD).

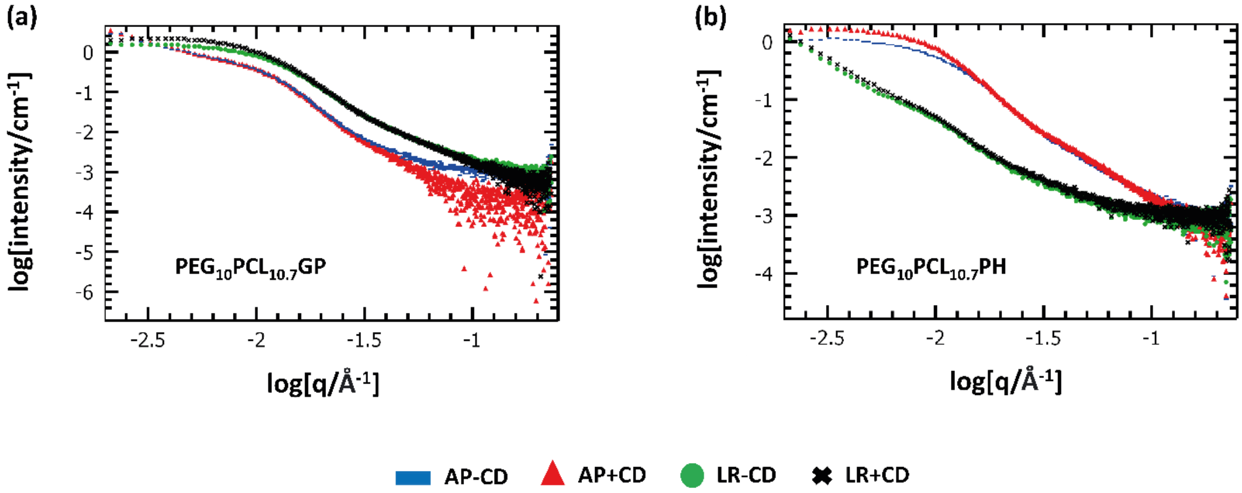

Figure S24. SAXS profiles for (a) gossypol and (b) phloretin loaded $PEG_{10}PCL_{10.7}$ micelles as prepared (AP) and after lyophilization/reconstitution (LR) in the absence and presence of β-CD (-/+CD).
